# Multi-phase field model reveals internal dissipation is crucial for spontaneous hole formation in cell monolayers

**DOI:** 10.1038/s41467-026-74977-y

**Published:** 2026-07-03

**Authors:** Diogo E. P. Pinto, Jan Rozman, Julia M. Yeomans

**Affiliations:** 1 The Rudolf Peierls Centre for Theoretical Physics, Clarendon Laboratory, Oxford, UK; 2 Centro de Física Teórica e Computacional, Faculdade de Ciências, Universidade de Lisboa, Lisboa, Portugal; 3 Departamento de Física, Faculdade de Ciências, Universidade de Lisboa, Lisboa, Portugal; 4 Jožef Stefan Institute, Ljubljana, Slovenia; 5 University of Ljubljana, Faculty of Mathematics and Physics, Ljubljana, Slovenia

**Keywords:** Biological physics, Statistical physics, Condensed-matter physics

## Abstract

Although cell monolayers typically remain confluent, they can spontaneously develop persistent holes as a result of collective cellular motion. Recent studies on MDCK monolayers cultured on soft substrates have revealed that cells can align to create regions of local nematic order, and topological defects that generate localised mechanical stresses which can spontaneously trigger hole formation. To investigate this process, we develop a continuum multi-phase field model that incorporates internal dissipation and active dipolar forces that drive cell shape anisotropy. Our simulations show that reducing substrate friction enhances cell-cell velocity correlations. In the low-friction regime, topological defects generate spiral flow patterns that concentrate stress and can trigger hole formation. By contrast, in the high-friction regime, holes do not nucleate. We further demonstrate that the number and stability of the holes—whether they close or persist—depends on both substrate friction and cellular activity, through a non-dimensional friction number. These findings highlight the importance of internal dissipation in modelling collective cell motion and the critical role of collective dynamics in maintaining tissue integrity.

## Abstract (web summary)

Internal dissipation enables long-range collective cell motion in epithelial monolayers. This allows topological defects to generate localised stresses that trigger spontaneous hole formation and govern tissue integrity.

## Introduction

Epithelial tissues serve as protective barriers for many organisms, covering organs and forming the outer layer of the skin. To maintain their integrity, these tissues must withstand significant mechanical stresses^1^, otherwise they fracture^2^, forming holes, which can lead to associated diseases^3^. A key mechanism by which epithelial tissues preserve their integrity is through coordinated cell motion^4–9^, mediated by intercellular contacts. These contacts form networks that transmit local mechanical forces across the tissue^10^, generating stress patterns that regulate processes such as cell division^11^, extrusion^12^, and migration^13^.

In some cases, the formation of holes in epithelia can be a crucial step in development. For instance, in multicellular organisms like *Trichoplax adhaerens*, collective movements of the epithelial sheet can drive spontaneous ruptures, serving as a unique mechanism of asexual reproduction^14^. However, understanding such phenomena still poses significant challenges, particularly in in vivo settings where imaging and mechanical characterisation are limited^15^. As a result, in vitro systems have become essential tools for investigating collective cell behaviour under controlled mechanical environments^16–19^.

Recent work on MDCK cell monolayers cultured on polyacrylamide gel substrates has revealed that substrate stiffness strongly influences tissue integrity^20^. In particular, tissues cultured on soft substrates can spontaneously form holes, whereas stiffer substrates preserve tissue confluency. The authors of that work highlight how active nematic theories^21,22^ can describe the collective motion of cells and the formation of topological defects (in the cell shape) before hole formation. These defects give rise to local gradients in stresses that can lead to rupture. Other work has similarly highlighted how the rheological properties of the substrate can influence cell-substrate adhesions, which, in turn, affect the collective behaviour of cells^23^. These observations provide compelling evidence for the role of the supporting structure in mediating the collective behaviour in cell monolayers, and in driving spontaneous hole formation.

Here we use an active nematic multi-phase field model that is able to capture the collective motion observed in cell monolayers to investigate spontaneous hole formation. Building on prior work^24^, we represent each cell as a deformable phase field, which evolves under internal passive forces, mechanical interactions with neighbouring cells, and internally generated active stresses arising from actomyosin contractility^25^. Inspired by recent advances^26–31^, we introduce internal dissipation, via cell-cell friction, leading to long-range correlations and collective motion, commonly seen in cell monolayers^32–34^. We demonstrate that, in this regime, spiral patterns in the velocity field emerge as a consequence of antiparallel alignment and motion of two  + 1/2 topological defects in the nematic director field. As the defects move apart, they generate large local strains that can initiate spontaneous hole formation. Our results show in particular that substrate friction modulates this behaviour: high friction suppresses long-range correlations, similar to continuum theories^35–37^, inhibiting hole formation, while reduced friction increases the likelihood of rupture. We also observe, in agreement with experiments, that, as the hole nucleates, local flows align cells along its border, thereby promoting further growth.

We highlight how hole formation, driven by large strain localisation near topological defects, follows similar statistics as observed in other disordered systems^38,39^. Due to activity, holes are highly dynamical and can heal. We identify a transition between regimes dominated by short-lived transient holes and those characterised by long-lived persistent rupture, which is governed by the interplay between activity and substrate friction. We confirm that the system’s behaviour is governed by two dominant length scales, the active and hydrodynamic length scales, which encode the balance of the relevant forces. Moreover, we demonstrate that a non-dimensional friction number, which links these two length scales, plays a central role: when expressed in terms of this parameter, measurements of the characteristic isotropic strain rate for hole nucleation, and of the typical hole lifetimes, collapse onto single curves. Our findings provide additional insights into how the mechanical support, via a substrate, regulates collective cell motion and mediates cell monolayer integrity.

## Results

We use a 2D multi-phase field model where each cell is described by an independent scalar phase field, *ϕ*_*i*_(**x**), that continuously varies from 0 to 1^40,41^. The motion of each phase field is governed by a local velocity field **v**_*i*_(**x**) according to the equation of motion

1 $${\partial }_{t}{\phi }_{i}({{\bf{x}}})+{{{\bf{v}}}}_{i}({{\bf{x}}})\cdot \nabla {\phi }_{i}({{\bf{x}}})=-{J}_{0}\frac{\delta {{\mathscr{F}}}}{\delta {\phi }_{i}({{\bf{x}}})},$$

where ${{\mathscr{F}}}$ is a free energy. The right-hand side of Eq. (1) corresponds to the relaxation dynamics of the cells to a free-energy minimum at a rate *J*_0_. The free energy term defines the dynamics of the individual interfaces and is written as ${{\mathscr{F}}}={{{\mathscr{F}}}}_{CH}+{{{\mathscr{F}}}}_{area}+{{{\mathscr{F}}}}_{rep}$, where

2 $${{{\mathscr{F}}}}_{CH}={\sum}_{i}\frac{\gamma }{\lambda }\int\,d{{\bf{x}}}\left[4{\phi }_{i}^{2}({{\bf{x}}}){(1-{\phi }_{i}({{\bf{x}}}))}^{2}+{\lambda }^{2}{(\nabla {\phi }_{i}({{\bf{x}}}))}^{2}\right],$$

3 $${{{\mathscr{F}}}}_{area}={\sum}_{i}\mu \left[1-\frac{1}{\pi {R}^{2}}\int\,d{{\bf{x}}}{\phi }_{i}^{2}({{\bf{x}}})\right],$$

4 $${{{\mathscr{F}}}}_{rep}={\sum}_{i}{\sum}_{j\ne i}\frac{\kappa }{\lambda }\int\,d{{\bf{x}}}{\phi }_{i}^{2}({{\bf{x}}}){\phi }_{j}^{2}({{\bf{x}}}).$$

Equation (2) is a Cahn-Hilliard free energy that encourages *ϕ*_*i*_(**x**) to be equal to 1 or 0 inside or outside cell *i*, respectively. The cell boundary is located at *ϕ*_*i*_(**x**) = 1/2 and has width  ~ 2*λ*, set by the gradient term. *γλ* sets an energy scale. This term controls the deformability of the individual cells. Cell compressibility is described by Eq. (3), which imposes a soft constraint, of strength *μ*, restricting the area of each cell to *πR*^2^. Equation (4) penalises overlap between cells with an energy scale *κλ*.

We recover the local velocity field by solving the force balance equation:

5 $${\xi }_{s}{{{\bf{v}}}}_{i}({{\bf{x}}})+\frac{{\xi }_{cell}}{\Delta \,{x}^{2}}{\sum}_{{{{\bf{x}}}}^{{\prime} }\in {{\mathcal{N}}}({{\bf{x}}})}{\sum}_{j\in {{{\bf{x}}}}^{{\prime} }}[{{{\bf{v}}}}_{i}({{\bf{x}}})-{{{\bf{v}}}}_{j}({{{\bf{x}}}}^{{\prime} })]={{{\bf{f}}}}_{i}^{p}({{\bf{x}}})+{{{\bf{f}}}}_{i}^{a}({{\bf{x}}}).$$

on a square lattice, with lattice spacing *Δx*. The first term on the left-hand side of Eq. (5) captures the dissipation due to friction from the relative motion of the cell on a substrate with a friction coefficient *ξ*_*s*_. The second term represents the dissipation from the relative motion between neighbouring cells and inside a given cell, with a corresponding friction coefficient *ξ*_*cell*_. ${{\mathcal{N}}}({{\bf{x}}})$ are the eight lattice sites surrounding **x**, and *j* corresponds to all phase fields at the lattice site ${{{\bf{x}}}}^{{\prime} }$. This term resembles a Laplacian of the velocity field at the lattice site **x**, resulting in a viscosity-like term similar to continuum models^21^. Recent work introduced a similar term to a multi-phase field model but with velocities at the level of the individual cell^28,29^. Our implementation allows for spatial variations in velocity within individual cells, thereby enabling the description of nematic activity.

The right-hand side of Eq. (5) includes passive and active force densities. The passive force density,

6 $${{{\bf{f}}}}_{i}^{p}({{\bf{x}}})=-\nabla \left(\frac{\delta {{\mathscr{F}}}}{\delta {\phi }_{i}({{\bf{x}}})}\right),$$

represents the passive drive to the minimum of the free energy ${{\mathscr{F}}}$. This formulation of the passive forces differs from previous approaches^24,40^, which can produce non-zero net forces and induce unidirectional flows under periodic boundary conditions, particularly at low substrate friction. In contrast, our formulation derives passive forces from a gradient term, ensuring a zero net force in periodic domains. Since the role of these forces is to drive the system toward free-energy minima while remaining globally force-balanced, we do not expect these differences to qualitatively affect the results.

The active force density,

7 $${{{\bf{f}}}}_{i}^{a}({{\bf{x}}})=-\zeta {{{\bf{Q}}}}_{i}\cdot \nabla {\phi }_{i}({{\bf{x}}}),$$

represents the active driving of the cell due to actomyosin activity. The strength of the activity is given by *ζ*, and **Q**_*i*_ is the nematic tensor that controls the direction of the active forces for each cell *i*. For simplicity, we assume that the nematic tensor follows the deformation tensor of the cell,

8 $${{{\bf{Q}}}}_{i}=-\int\,d{{\bf{x}}}\left[\nabla {\phi }_{i}\nabla {\phi }_{i}^{T}-\frac{1}{2}{{\rm{Tr}}}(\nabla {\phi }_{i}\nabla {\phi }_{i}^{T})\right],$$

so that the eigenvectors of this tensor, **d**^∥^ and **d**^⊥^, lie along and perpendicular to the elongation of the cell, respectively, and we normalise **Q**_*i*_ by $0.5\sqrt{{Q}_{xx}^{2}+{Q}_{xy}^{2}}$. Previous works have shown that the collective motion of cell monolayers resembles that of an extensile active nematic system^33,42^. Thus, we focus on the extensile regime, *ζ* > 0. See Supplementary Information Sec. A and refs. ^24,43^ for more details of the model.

One can non-dimensionalise the force balance equation, Eq. (5), by introducing the characteristic length and time scales, *L*_0_ and *T*_0_ respectively. This leads to the following dimensionless variables:

9 $$\bar{{{\bf{x}}}}=\frac{{{\bf{x}}}}{{L}_{0}},\bar{t}=\frac{t}{{T}_{0}},\bar{{{\bf{v}}}}=\frac{{{\bf{v}}}}{{V}_{0}},$$

where *V*_0_ = *L*_0_/*T*_0_ is the velocity scale. We can introduce these variables in Eq. (5) and reach the following non-dimensionalised equation:

10 $${\bar{{{\bf{v}}}}}_{i}(\bar{{{\bf{x}}}})+\frac{{l}_{h}^{2}}{{L}_{0}^{2}\Delta {\bar{x}}^{2}}\sum\limits_{{{{\bf{x}}}}^{{\prime} }\in {{\mathcal{N}}}({{\bf{x}}})}\sum\limits_{j\in {{{\bf{x}}}}^{{\prime} }}[{\bar{{{\bf{v}}}}}_{i}(\bar{{{\bf{x}}}})-{\bar{{{\bf{v}}}}}_{j}({\bar{{{\bf{x}}}}}^{{\prime} })]={{{\bf{f}}}}_{i}^{p}(\bar{{{\bf{x}}}})/{\xi }_{s}{V}_{0}-\bar{A}{{{\bf{Q}}}}_{i}\cdot \bar{\nabla }{\phi }_{i}(\bar{{{\bf{x}}}}),$$

where ${l}_{h}=\sqrt{{\xi }_{cell}/{\xi }_{s}}$ and $\bar{A}=\zeta {T}_{0}/{\xi }_{s}{L}_{0}^{2}$. From this equation we can identify one of the relevant length scales, the hydrodynamic length scale, *l*_*h*_, which sets the typical distance over which hydrodynamic-like effects (coming from cell-cell friction) are relevant. For the passive energy term, we consider, to first order, that the main contribution for the passive restoring force density comes from the *γλ*∇^2^*ϕ* term given by the Cahn-Hilliard free energy, representing a penalty for distortions in the shape of the cells. We then introduce an active length scale ${l}_{a}=\sqrt{\gamma \lambda /| \zeta | }$, that denotes the characteristic length scale where active stresses overcome passive restoring forces. Combining these two length scales, we define the non-dimensional friction number, $\bar{F}={l}_{h}/{l}_{a}$^36^. We adopt the characteristic length scale *L*_0_ = *λ*, which sets the interface width where the relevant dynamics occur, and, based on dimensional analysis of (*ξ*_*s*_, *ξ*_*cell*_, *ζ*, *λγ*), the characteristic time scale ${T}_{0}={\xi }_{{cell}}^{2}/({\xi }_{s}\gamma \lambda )\equiv {\tau }_{F}$.

In the following, we consider a system of *N* = 100 deformable cells, with a radius of *R* = 8 in lattice units, which move on an underlying periodic square lattice of side *L* = 140. The lattice spacing is set to *Δx* = 1. These choices lead to packing fractions above 95% meaning that the cells are confluent, tightly packed with no gaps between them. We choose parameters, which are detailed in Sec. A of the Supplementary Information, to target the low hole density regime, where the typical distance between holes in the monolayer is much greater than the holes’ characteristic size, so that it is reasonable to discount hole-hole interactions. The equations of motion (1) and (5) are integrated using a finite difference method in space and a predictor-corrector method in time^40^. We initialise the system by positioning the cells randomly in the simulation box with a starting radius of *R*/2. We then let the system relax without activity and cell-cell friction for 10,000 time-steps, after which we simulate with activity and cell-cell friction for another 100,000 time-steps.

Based on typical values for MDCK cells, an average radius of approximately 10 μm, velocity around 20 μm/h, and pressure on the order of 100 Pa, as measured via Particle Image Velocimetry and Traction Force Microscopy ^33^, we estimate that the simulation units correspond to physical units of *Δx* ~ 1 *μ*m, *Δt* ~ 5 s, and *ΔF* ~ 1.5 nN for length, time, and force, respectively (a more detailed table for all parameters can be found in the Supplementary Information Sec. A).

To focus on the effects of substrate friction, cell-cell friction and activity on the collective behaviour of cell monolayers we use the non-dimensional friction number, $\bar{F}={l}_{h}/{l}_{a}$, as a measure of the ratio between the hydrodynamic screening length and the active length scale^36,44^. This parameter emphasises the competition between the relevant forces in the system. In particular, for the hydrodynamic screening length, we observe that when *l*_*h*_ ≲ *λ*, the dynamics of the system is similar to the pure substrate friction case (*ξ*_*cell*_ = 0), as the characteristic length scale of velocity and nematic correlations is smaller than the typical cell size (see Supplementary Information Sec. B). In this regime, the motion of the cells is random, and no holes form in the monolayer, for any value of the active length scale, *l*_*a*_. When the substrate friction is sufficiently low (*l*_*h*_ ≫ *λ*) that the characteristic length scale of correlations is larger than the cell size (see Supplementary Information Sec. B), and the activity is large enough to compete with the elastic restoring force of the monolayer, the system enters a regime similar to active turbulence^21^, and holes spontaneously form. This observation underscores how cell-cell friction can be a key physical ingredient to accurately realise monolayer collective behaviour.

### Mechanics of hole nucleation

**Fig. 1:**
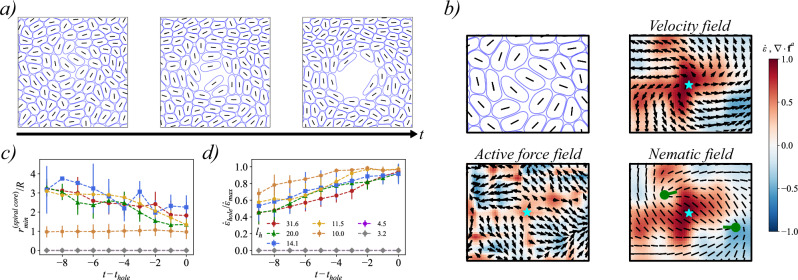
A model cell monolayer during hole formation. **a** Snapshots showing the evolution of the system as a hole nucleates in the monolayer. The boundary of the cells are drawn at *ϕ*_*i*_(**x**) = 0.5 and the black line inside each cell corresponds to the elongation axis. In **b** we zoom in on the area where hole nucleation occurs and image various properties of the monolayer: velocity, active force, and nematic fields. In the velocity field, the cyan star corresponds to the location of a spiral core (also represented in the other fields for reference). In the nematic field, the green dots represent the location of  + 1/2 topological defects with a line pointing towards the tail of the defect. The colours in the velocity and nematic fields represent the local normalised isotropic strain rate calculated from the velocity field. In the active force field, the colours represent the normalised magnitude of the divergence of the active force. The colour bar highlights these normalised quantities for the respective figures. All fields are smoothed using a sliding window of size 2*R* × 2*R*. **c** Distance from the hole’s centroid to the closest spiral core, normalised by the cell radius *R*, as a function of the number of time-frames before hole nucleation, where 0 corresponds to a hole nucleation event. **d** Normalised maximum isotropic strain rate in an area of radius *R*/2 around the location of hole nucleation as a function of the number of time-frames before nucleation. The normalisation is per time-frame, where each time-frame spans 1000 time-steps. The results shown in both **c**, **d** were averaged over 10 simulations with 100 time-frames, and with constant *ξ*_*cell*_ = 1. In **c**, **d**, the points at zero distance and isotropic strain rate, respectively, represent simulations which do not nucleate holes (*l*_*h*_ = [3.2, 4.5]). For *l*_*h*_ = 10.0 the number of holes formed is small, resulting in poor statistics that artificially flatten the curve’s behaviour. For **c**, **d** the data are presented as mean values ± SD. Source data are provided as a Source Data file.

Figure 1a shows a schematic with sequential snapshots of the model cell monolayer during a typical hole nucleation event (see Supplementary Video S1 for the full simulation). Figure 1b represents the velocity, force, and nematic fields just before hole nucleation (with Supplementary Videos S2, S3, and S4 respectively). We observe that close to a nucleation event, the cells are arranged in a specific pattern, which is characterised by two  + 1/2 topological defects in the nematic field moving in the vicinity of each other in an antiparallel relative orientation. This correlated motion leads to a net active force pointing outwards from the axis of the defect rotation and the formation of a spiral in the velocity field (point where the divergence of the velocity field is positive). Since the monolayer is compressible, if the active forcing and flows are large enough to overcome the passive forces that keeps the monolayer confluent, a hole can spontaneously form. We confirm the role of pairs of  + 1/2 topological defects in Sec. C of the Supplementary Information, by looking at the distance from the hole to the closest defects.

We show that this spatial arrangement is crucial by plotting, in Fig. 1c, d, the distance from the hole’s centroid to the closest spiral core in the velocity field and the maximum isotropic strain rate, respectively, at the moment of hole formation, *t* − *t*_hole_ = 0. The *x*-axis of these plots then corresponds to the number of time-frames before the nucleation event (each time-frame corresponds to 1000 time-steps). The results suggest that, just before the hole forms, both the peak isotropic strain rate in the monolayer and the nearest spiral core localise near the nucleation point (within approximately one cell diameter). These observations highlight how collective motion leads to the localisation of strains, driven by active forces, culminating in rupture. In Supplementary Information Sec. G, we show that the deviatoric component of the strain rate tensor also peaks near hole nucleation (see Supplementary Video S5), indicating strong local distortions of the tissue that contribute to the formation of the spiral flow patterns observed in the velocity field.

If the activity is too low, the hole is not able to form. This is because the active forces are not large enough to overcome the energy barrier that keeps the monolayer confluent. To confirm this, we checked that as the surface tension of the cells increases, thus making cells less deformable and increasing the effective elastic response of the monolayer, the probability of forming a hole decreases (see Supplementary Information Sec. D).

**Fig. 2:**
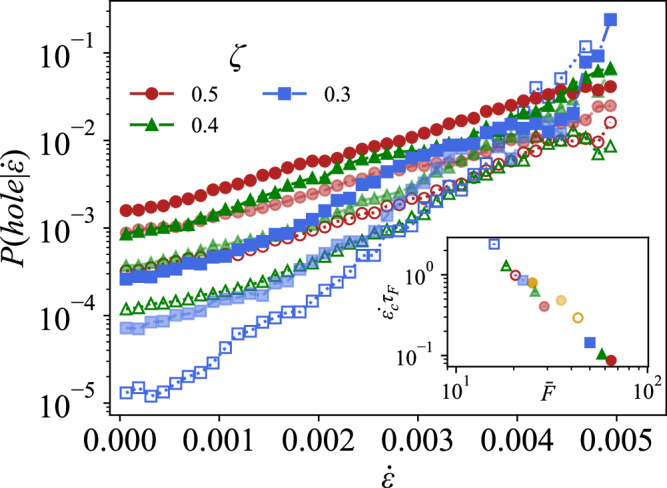
Conditional probability of forming a hole for a given isotropic strain rate as a function of local isotropic strain rate and activity . The main plot shows a log-linear scale to highlight the exponential form. The isotropic strain rate field is calculated from the divergence of the velocity field. Each isotropic strain rate point corresponds to $\dot{\varepsilon }+\Delta \dot{\varepsilon }$, where $\Delta \dot{\varepsilon }=0.000125$. The inset shows the characteristic isotropic strain rate for hole nucleation, ${\dot{\varepsilon }}_{c}$, non-dimensionalised by the timescale ${\tau }_{F}={\xi }_{cell}^{2}/{\xi }_{s}\gamma \lambda$, as a function of $\bar{F}$. The data points collapse onto a single line with slope  − 3 on a log–log scale. (empty symbols correspond to *ξ*_*s*_ = 0.01, partially transparent symbols to *ξ*_*s*_ = 0.05, and full symbols to *ξ*_*s*_ = 0.001) The golden points represent simulations for *ζ* = 0.3, *ξ*_*s*_ = 0.01, and *ξ*_*cell*_ = (0.75, 0.5, 0.25), for empty, partially transparent, and full symbols respectively. Each distributions is averaged over 100 different simulations with 100 different time-frames. Source data are provided as a Source Data file.

Given that the passive response keeps the monolayer confluent, the isotropic strain rate generated by the active forces needs to be large enough to overcome this energy barrier. Therefore, not all spiral patterns lead to holes, only those that localise enough strain. Figure 2 shows the conditional probability of forming a hole given a local isotropic strain rate, $P(hole| \dot{\varepsilon })$. We measure this by first calculating the isotropic strain rate field across the monolayer, for each time-frame. We then count ${N}_{\dot{\varepsilon }}$, the total number of lattice sites across all time frames and all simulations for which the corresponding isotropic strain rate is within a given interval $\dot{\varepsilon }+\Delta \dot{\varepsilon }$. For each isotropic strain rate interval, we also check how many of the counted lattice sites open into a hole in the subsequent time-frame, denoted as ${N}_{hole,\dot{\varepsilon }}$. Thus, $P(hole| \dot{\varepsilon })={N}_{hole,\dot{\varepsilon }}/{N}_{\dot{\varepsilon }}$. Note that if a hole grows above a threshold size, corresponding to half of a single cell area, lattice sites are not counted towards either ${N}_{hole,\dot{\varepsilon }}$ or ${N}_{\dot{\varepsilon }}$ until the hole closes, as the system size cannot accommodate more than one hole at the same time.

The main plot in Fig. 2 shows that $P(hole| \dot{\varepsilon })$, for different activities and substrate frictions, is well fitted by an exponential. This confirms that high isotropic strain rates are exponentially more likely to form a hole, as cells need to generate enough force to overcome the passive response. We remark that the coefficient of the exponential decreases with activity and increases with substrate friction. Higher activity or lower substrate friction generate larger isotropic strain rates, but the fraction of the large isotropic strain rates that nucleate holes decreases. We hypothesise that this is because there are competing regions of high isotropic strain rate for higher activities and lower substrate frictions, so multiple regions of the monolayer may experience large isotropic strain rates. However, since steric constraints mean that only one hole can form at a time, only one of the high isotropic strain rate regions gives rise to a hole. The inset in Fig. 2 plots the coefficient of the different exponentials, the characteristic isotropic strain rate for hole nucleation ${\dot{\varepsilon }}_{c}$ against the non-dimensional friction number $\bar{F}$. We use the time-scale *τ*_*F*_ to non-dimensionalise the isotropic strain rate. We observe that this is enough to collapse all the data points onto a single curve, which has slope  − 3 in a log-log scale, suggesting that ${\dot{\varepsilon }}_{c}{\tau }_{F} \sim {\bar{F}}^{-3}$. A broader parameter range will be needed to confirm this scaling.

Defect-driven fracture is reminiscent of other soft disordered materials like glasses and polymer networks^38,39^, which are highly heterogeneous and whose fracture, when under externally applied stress, is initiated by the accumulation of stresses near defects in the material. However, unlike passive systems, cells are highly motile and active. This means that the monolayer can rupture without any externally applied stresses^45,46^; the collective motion of the cells can spontaneously generate local stresses (and strains) that lead to holes. Moreover, rupture is a more dynamical process, where holes can grow, shrink, annihilate, and nucleate again at a later time.

### Statistics of hole dynamics

**Fig. 3:**
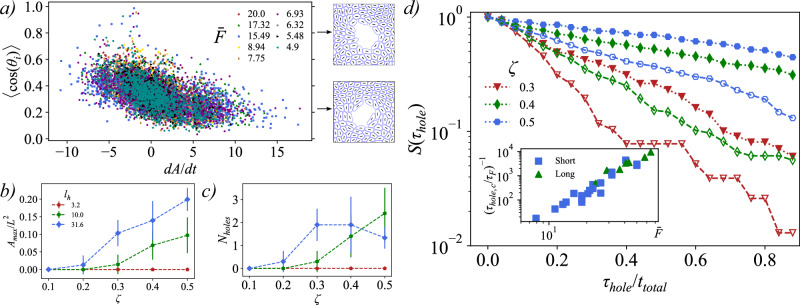
Cell alignment and hole lifetime statistics. **a** Scatter plot where the *y*-axis reports the average cell alignment along the hole interface, quantified by $\langle \cos ({\theta }_{i})\rangle$, where *θ*_*i*_ is the angle between the nematic director of cell *i* and the normal to the hole interface, and the *x*-axis shows the change in hole area over successive time-frames. Two simulation snapshots are shown on the right, one for high $\langle \cos ({\theta }_{i})\rangle \approx 0.8$ (top) and another for low $\langle \cos ({\theta }_{i})\rangle \approx 0.2$ (bottom). The Pearson correlation coefficient for this data set is  − 0.56. **b** Normalised maximum hole area and **c** total number of holes integrated over the whole simulation as a function of activity for different substrate frictions (*l*_*h*_ = [3.2, 10.0, 31.6]). **d** Survival probability of holes, i.e, the probability that a hole survives a fraction, *τ*_hole_/*t*_total_, of the full simulation time, *t*_total_, for different activities (*ζ* = [0.3, 0.4, 0.5]) and substrate frictions (empty symbols correspond to *l*_*h*_ = 14.1 and full symbols to *l*_*h*_ = 31.6), on a log-linear scale. We fit an exponential to each curve to calculate the characteristic hole lifetime, *τ*_hole,*c*_. The inset shows how the data is collapsed for *τ*_hole,*c*_/*τ*_*F*_ as a function of $\bar{F}$, recovering the cubic exponent in a log-log scale. The data points are separated into short and long lived holes, corresponding to *τ*_hole_/*t*_total_ < 0.5 (blue squares) and *τ*_hole_/*t*_total_ > 0.5 (green triangles), respectively. The results in **b**, **c** were each averaged over 10 simulations with 100 time-frames. The results in **d** were each averaged over 100 simulations with 100 time-frames. For **b**, **c** the data are presented as mean values ± SD. Source data are provided as a Source Data file.

Higher activity generates the forces required to overcome the elastic response of the monolayer. Notably, reduced substrate friction enhances spatial correlations, giving rise to coherent velocity fields that nucleate holes and stabilise them by promoting tangential alignment of cells along the tissue-hole interface (see simulation snapshots in Fig. 3a). This alignment is crucial: when cells at the interface are oriented tangentially, the active forces they generate act predominantly in the radial direction, opposing the passive forces that drive hole closure (see Supplementary Video S6). For a hole to shrink, this tangential alignment must be disrupted by neighbouring cells. Consequently, increasing activity or decreasing substrate friction promotes cell alignment and enhances hole stability.

In Fig. 3a, we quantify cell alignment at the hole interface by measuring the angle *θ*_*i*_ between the nematic director of cell *i* (extracted from Eq. (8)) and the normal vector to the hole interface. This angle is averaged over all lattice sites shared between cell *i* and the hole, and then averaged over all cells surrounding the hole to obtain $\langle \cos ({\theta }_{i})\rangle$. Low values of $\langle \cos ({\theta }_{i})\rangle$ indicate cells aligned tangentially to the interface, whereas high values correspond to alignment normal to it.

Figure 3a presents a scatter plot with $\langle \cos ({\theta }_{i})\rangle$ on the *y*-axis and the change in hole area between successive time-frames on the *x*-axis. We observe a clear anti-correlation between cell elongation at the interface and hole dynamics: holes tend to grow when cells are tangentially aligned, whereas they tend to shrink when the tangential alignment is broken. The Pearson correlation coefficient for this data set is  − 0.56, with a p-value *P* < 0.001 (*n*_samples_ = 10^4^, *t*(9998) = 67.59), indicating a statistically significant inverse relationship between the two variables. This behaviour is reminiscent of active anchoring ^47^ and provides a mechanism for both the stabilisation and growth of holes.

Figure 3b, c characterise the maximum area and total number of holes as a function of activity and substrate friction, respectively. As either the activity increases or substrate friction decreases, the maximum area of holes increases as the active forces become more able to counteract the passive forces in the monolayer that promote confluency. Conversely, the total number of holes integrated over the whole simulation varies non-monotonically with activity and substrate friction. This occurs because the typical lifetime of holes increases with increasing activity and decreases with increasing substrate friction. For low activities (or high substrate friction) no holes are able to form. For higher values of activity, holes can nucleate and their lifetime increases as activity increases (or substrate friction decreases), eventually reaching a lifetime comparable to the total simulation time. Since only one hole can form at a time, long lifetimes prevent new ones from forming. This is quantified in Fig. 3d where we plot the survival probability of the holes, i.e., the probability that a hole survives *τ*_*hole*_ units of time after nucleation. Note that, above a given value of activity, there are holes that are able to last the entire duration of the simulation (see Supplementary Video S7).

The survival probabilities in Fig. 3d are well fitted by an exponential distribution, allowing us to extract the characteristic hole-lifetime timescale, *τ*_hole,*c*_. As in Fig. 2, we non-dimensionalise this quantity using the timescale *τ*_*F*_. The inset in Fig. 3d shows that the non-dimensional lifetime, *τ*_hole,*c*_/*τ*_*F*_, collapses when plotted as a function of the non-dimensional friction number, $\bar{F}$. This collapse further underscores the importance of the two governing length scales, *l*_*h*_ and *l*_*a*_, in controlling both hole formation and subsequent dynamics. Once again, we find a cubic scaling, ${\tau }_{hole,c}/{\tau }_{F} \sim {\bar{F}}^{-3}$, reinforcing the scaling already observed in Fig. 2. In experiments, holes are categorised as short or long-lived depending on their lifetime^20^. We consider the probability of forming persistent holes to be non-negligible when the lifetime exceeds a threshold *τ*_hole_ > 0.5, *t*_total_, where *t*_total_ is the full simulation time. In physical units, this threshold corresponds to *τ*_hole_ ~ 7 h, consistent with the onset of long-lived holes observed experimentally^20^.

## Discussion

We develop an active nematic multi-phase field model, with both substrate and internal dissipation, to study hole formation in cell monolayers. We show that holes only form spontaneously when velocity correlations reach beyond the typical size of a single cell. The hole formation is driven by the relative motion of pairs of  + 1/2 topological defects in the nematic field generating spiral velocity patterns, which serve as nucleation sites for the holes. The collective motion leads to local stretching of the monolayer, triggering rupture. Once formed, the resulting flows tend to align cells tangentially along a hole’s edge, promoting radial expansion. However, particularly for lower activities, the alignment is often disrupted by competing cell flows so that holes have a finite lifetime.

We quantify the rupture statistics by measuring the probability of forming holes for a given local isotropic strain rate, which follows an exponential form. This defect-driven fracture process is similar to other soft disordered systems^38,39^ which are characterised by their high heterogeneity and stress localisation near material defects. However, here, because a cell monolayer is active, holes can be highly dynamic with a lifetime that can be increased by reducing substrate friction or increasing activity. We introduce a non-dimensional friction number, $\bar{F}$, which allows us to collapse the data and reveals the relevant scaling relations between both the characteristic isotropic strain rate for hole nucleation and the typical hole lifetime and the friction number. This scaling highlights the key length scales governing hole formation and dynamics, in particular the hydrodynamic and active length scales.

Long-range flows underlie multiple processes in biology, from the formation of the primitive streak in the chicken embryo^48^ to glioblastoma tumour progression^49^. Incorporating internal dissipation into the multi-phase field approach used here has proven essential to generate the flow correlations^26,27^ needed to create holes.

In the friction dominated regime (*l*_*h*_ ≲ *λ*), no holes form spontaneously. Previous work which considered this regime found small gaps in simulations of a multi-phase field model^37^. However, these are typically smaller than the cell size and are driven by a lower effective packing fraction because activity drives elongation of the cells which reduces the effective occupied area^37^. In our simulations this is not the case; when no hole is present the packing fraction is always above 95% and, when a hole is formed, no other gaps appear in the monolayer. This is strong evidence that the hole nucleation process in our model is indeed due to collective motion and localisation of stresses around the nucleation area, and not due to the lowering of the effective packing fraction or de-wetting of the monolayer (as seen in epithelioid tissues^50^). In Sec. D of the Supplementary Information we corroborate this by showing that when we increase the elasticity of the monolayer (through the surface tension of the cells) the probability of nucleating holes decreases and in Sec. E of the Supplementary Information we explore how an explicit cell-cell adhesion term can affect the hole formation.

Recent experiments on MDCK cells have shown a similar hole nucleation process mediated by the substrate, in which holes in the monolayer nucleate on soft substrates but not on stiffer ones^20^. Although ref. ^20^ does not explicitly address substrate friction, since cell-substrate adhesions are not measured, the authors report higher traction forces on stiffer substrates, consistent with the higher friction regime in our model. They show that the cells move collectively, generating flows and forming  + 1/2 and  − 1/2 topological defects, and that hole formation correlates with the location of the defects, where stress gradients are highest. They also suggest that tangential alignment of cells at the hole boundary is crucial to hole formation.

Our results propose further experiments and predictive capabilities for the extracted data. A key experiment would be to modulate tissue viscosity, for example by treatment with cytochalasin D which reduces the relative intensity of E-cadherin at cell-cell junctions^51^, and then assess the resulting effects on hole nucleation. Also, we find that, to facilitate local stretching of the monolayer, two  + 1/2 defects oriented antiparallel to each other are needed to create the spiral flows that drive hole nucleation, which suggests investigating defect pairs and velocity spiral positions prior to hole formation in the experiments. We note that defects leading to spiral patterns in the cell velocity field have also been shown to modulate collective flows in bacterial colonies^52^ and influence developmental and regenerative processes in Hydra^53^. More quantitatively, measuring the characteristic lifetime of holes would provide a direct test of the cubic scaling and data collapse identified here (${\bar{F}}^{-3}$). In addition, measuring the correlations between cell alignment around the holes and the rate of hole growth will provide a further point of comparison between simulations and experiments.

In contrast to the experiments we do not see hole formation at  − 1/2 defects in the simulations. This may be due to not taking anisotropic effects into account. Hole formation at  − 1/2 defects in bacterial colonies^54^ has been connected to anisotropic friction effects and introducing this into the multi-phase field model will be an interesting topic for future research. Although cell division may also contribute to anisotropic effects, experiments on mitomycin-C treated monolayers^20^ indicate that division is not a necessary condition for hole formation. The persistence of this behaviour in the absence of cell divisions suggests that cell stretching constitutes a primary driving mechanism. Another outstanding question is whether modelling the activity using random polar^55,56^, rather than nematic, forces leads to any changes in behaviour.

It would also be of interest to compare the results presented here to other modelling approaches. For continuum models, the use of a similar non-dimensional friction number has been proposed before^36^, but in the context of incompressible flows, so no holes could form. Introducing compressibility would allow for a confirmation of the scaling relations found here, and of the role of active anchoring in controlling the hole dynamics in an approach able to access longer length scales, though at a loss of cell-scale resolution.

We further comment that cell monolayers can serve as a useful biological model for applying ideas from the statistical physics of fracture to biological systems. Previous studies have examined how these systems respond to external stresses and how their elasticity changes with the rate of deformation^57–59^, sometimes leading to effects like strain stiffening^60^. Our results indicate that active systems can spontaneously form defects and generate stresses that affect their own integrity. This dynamic behaviour suggests the need to extend the definition of failure in such materials and highlights the importance of healing^7,8,61,62^ as part of their mechanical response.

### Reporting summary

Further information on research design is available in the Nature Portfolio Reporting Summary linked to this article.

## Supplementary information

Supplementary Movie 1

Supplementary Movie 2

Supplementary Movie 3

Supplementary Movie 4

Supplementary Movie 5

Supplementary Movie 6

Supplementary Movie 7

Supplementary Information

Description of Additional Supplementary Files

Reporting Summary

Transparent Peer Review file

## Source data

Source Data

## Data Availability

The initial configuration files, topology files, and input parameters used in this study have been deposited in the Zenodo database under the 10.5281/zenodo.20591771. Source data are provided with this paper.

## Code availability

Data were generated using custom simulations of a two-dimensional active nematic multi-phase field model implemented in custom numerical code. Data analysis and visualisation were performed using custom analysis scripts and numerical post-processing routines. The equations of motion were integrated using finite-difference spatial discretization and a predictor-corrector time-stepping scheme, as described in the manuscript. All custom code and scripts used in this study can be found in the following Github repository: https://github.com/deppinto/RheoCell.

## References

[1] Ladoux, B. & Mège, R.-M. Mechanobiology of collective cell behaviours. *Nat. Rev. Mol. Cell Biol.* **18**, 743 (2017). 29115298 10.1038/nrm.2017.98

[2] Casares, L. et al. Hydraulic fracture during epithelial stretching. *Nat. Mater.* **14**, 343 (2015). 25664452 10.1038/nmat4206 PMC4374166

[3] Vlahakis, N. E. & Hubmayr, R. D. Response of alveolar cells to mechanical stress. *Curr. Opin. Crit. Care* **9**, 2 (2003). 12548022 10.1097/00075198-200302000-00002

[4] Boocock, D., Hino, N., Ruzickova, N., Hirashima, T. & Hannezo, E. Theory of mechanochemical patterning and optimal migration in cell monolayers. *Nat. Phys.* **17**, 267 (2021).

[5] Friedl, P. & Gilmour, D. Collective cell migration in morphogenesis, regeneration and cancer. *Nat. Rev. Mol. Cell Biol.* **10**, 445 (2009). 19546857 10.1038/nrm2720

[6] Rørth, P. Collective cell migration. *Annu. Rev. Cell Dev. Biol.* **25**, 407 (2009). 19575657 10.1146/annurev.cellbio.042308.113231

[7] Sarate, R. M. et al. Dynamic regulation of tissue fluidity controls skin repair during wound healing. *Cell* **187**, 5298 (2024). 39168124 10.1016/j.cell.2024.07.031

[8] Kreeger, P. K., Masters, K. S., Pomerenke, S., Mitchell, I. & Wickert, L. E. Collective migration of an epithelial monolayer in response to a model wound. *Proc. Natl. Acad. Sci. USA*. **104**, 15988 (2007). 17905871 10.1073/pnas.0705062104 PMC2042149

[9] Di Meglio, I. et al. Pressure and curvature control of the cell cycle in epithelia growing under spherical confinement. *Cell Rep.* **40**, 111227 (2022). 36001958 10.1016/j.celrep.2022.111227 PMC9433880

[10] Saraswathibhatla, A., Henkes, S., Galles, E. E., Sknepnek, R. & Notbohm, J. Coordinated tractions increase the size of a collectively moving pack in a cell monolayer. *Extrem. Mech. Lett.* **48**, 101438 (2021).

[11] Campinho, P. et al. Tension-oriented cell divisions limit anisotropic tissue tension in epithelial spreading during zebrafish epiboly. *Nat. Cell Biol.* **15**, 1405 (2013). 24212092 10.1038/ncb2869

[12] Eisenhoffer, G. T. et al. Crowding induces live cell extrusion to maintain homeostatic cell numbers in epithelia. *Nature* **484**, 546 (2012). 22504183 10.1038/nature10999 PMC4593481

[13] Collinet, C. & Lecuit, T. Programmed and self-organized flow of information during morphogenesis. *Nat. Rev. Mol. Cell Biol.* **22**, 245 (2021). 33483696 10.1038/s41580-020-00318-6

[14] Prakash, V. N., Bull, M. S. & Prakash, M. Motility-induced fracture reveals a ductile-to-brittle crossover in a simple animal’s epithelia. *Nat. Phys.* **17**, 504 (2021).

[15] Roca-Cusachs, P., Conte, V. & Trepat, X. Quantifying forces in cell biology. *Nat. Cell Biol.* **19**, 742 (2017). 28628082 10.1038/ncb3564

[16] Gómez-González, M., Latorre, E., Arroyo, M. & Trepat, X. Measuring mechanical stress in living tissues. *Nat. Rev. Phys.* **2**, 300 (2020). 39867749 10.1038/s42254-020-0184-6 PMC7617344

[17] Angelini, T. E., Hannezo, E., Trepat, X., Fredberg, J. J. & Weitz, D. A. Cell migration driven by cooperative substrate deformation patterns. *Phys. Rev. Lett.* **104**, 168104 (2010). 20482085 10.1103/PhysRevLett.104.168104 PMC3947506

[18] Murrell, M. P., Kamm, R. D. & Janmey, P. A. Substrate viscosity enhances correlation in epithelial sheet movement. *Biophys. J.* **101**, 297 (2011). 21767481 10.1016/j.bpj.2011.05.048 PMC3136779

[19] Vazquez, K., Saraswathibhatla, A. & Notbohm, J. Effect of substrate stiffness on friction in collective cell migration. *Sci. Rep.* **12**, 2474 (2022). 35169196 10.1038/s41598-022-06504-0 PMC8847350

[20] Sonam, S. et al. Mechanical stress driven by rigidity sensing governs epithelial stability. *Nat. Phys.* **19**, 132 (2023). 36686215 10.1038/s41567-022-01826-2 PMC7614076

[21] Doostmohammadi, A., Ignés-Mullol, J., Yeomans, J. M. & Sagués, F. Active nematics. *Nat. Commun.* **9**, 3246 (2018). 30131558 10.1038/s41467-018-05666-8 PMC6104062

[22] Simha, R. A. & Ramaswamy, S. Hydrodynamic fluctuations and instabilities in ordered suspensions of self-propelled particles. *Phys. Rev. Lett.* **89**, 058101 (2002). 12144468 10.1103/PhysRevLett.89.058101

[23] Zheng, J. Y. et al. Epithelial monolayers coalesce on a viscoelastic substrate through redistribution of vinculin. *Biophys. J.* **113**, 1585 (2017). 28844472 10.1016/j.bpj.2017.07.027 PMC5627150

[24] Zhang, G. & Yeomans, J. M. Active forces in confluent cell monolayers. *Phys. Rev. Lett.* **130**, 038202 (2023). 36763395 10.1103/PhysRevLett.130.038202

[25] Prost, J., Jülicher, F. & Joanny, J. Active gel physics. *Nat. Phys.* **11**, 111 (2015).

[26] Fu, C. et al. Regulation of intercellular viscosity by E-cadherin-dependent phosphorylation of EGFR in collective cell migration. *Proc. Natl. Acad. Sci. USA*. **121**, e2405560121 (2024). 39231206 10.1073/pnas.2405560121 PMC11406304

[27] Rozman, J., Chaithanya, K. V. S., Yeomans, J. M. & Sknepnek, R. Vertex model with internal dissipation enables sustained flows. *Nat. Commun.* **16**, 530 (2025). 39789022 10.1038/s41467-025-55820-2 PMC11718050

[28] Chiang, M., Hopkins, A., Loewe, B., Marenduzzo, D. & Marchetti, M. C. Multiphase field model of cells on a substrate: from three dimensional to two dimensional. *Phys. Rev. E* **110**, 044403 (2024). 39562868 10.1103/PhysRevE.110.044403

[29] Chiang, M., Hopkins, A., Loewe, B., Marchetti, M. C. & Marenduzzo, D. Intercellular friction and motility drive orientational order in cell monolayers. *Proc. Natl. Acad. Sci. USA*. **121**, e2319310121 (2024). 39302997 10.1073/pnas.2319310121 PMC11459176

[30] Barrett, K. et al. Epithelial-mesenchymal interface guides cell shapes and axis elongation in embryonic explants. *Development* **152**, dev204344 (2025). 40605737 10.1242/dev.204344

[31] Kammeraat, S. C. et al. Correlated cell movements drive epithelial finger formation. Preprint at 10.48550/arXiv.2508.01046 (2025).

[32] Andersen, B. H. et al. Evidence of universal conformal invariance in living biological matter. *Nat. Phys.* **21**, 618 (2025). 40248570 10.1038/s41567-025-02791-2 PMC11999873

[33] Saw, T. B. et al. Topological defects in epithelia govern cell death and extrusion. *Nature* **544**, 212 (2017). 28406198 10.1038/nature21718 PMC5439518

[34] Nejad, M. R. et al. Stress-shape misalignment in confluent cell layers. *Nat. Commun.* **15**, 3628 (2024). 38684651 10.1038/s41467-024-47702-w PMC11059169

[35] Krajnc, M., Stern, T. & Zankoc, C. Active instability and nonlinear dynamics of cell-cell junctions. *Phys. Rev. Lett.* **127**, 198103 (2021). 34797151 10.1103/PhysRevLett.127.198103

[36] Thijssen, K., Nejad, M. R. & Yeomans, J. M. Role of friction in multidefect ordering. *Phys. Rev. Lett.* **125**, 218004 (2020). 33275020 10.1103/PhysRevLett.125.218004

[37] Ardaševa, A., Mueller, R. & Doostmohammadi, A. Bridging microscopic cell dynamics to nematohydrodynamics of cell monolayers. *Soft Matter* **18**, 4737 (2022). 35703313 10.1039/d2sm00537a

[38] Morankar, S. et al. Tensile and fracture behavior of silica fibers from the venus flower basket (euplectella aspergillum). *Int. J. Solids Struct.* **253**, 111622 (2022).

[39] Cipelletti, L., Martens, K. & Ramos, L. Microscopic precursors of failure in soft matter. *Soft Matter* **16**, 82 (2020). 31720666 10.1039/c9sm01730e

[40] Mueller, R., Yeomans, J. M. & Doostmohammadi, A. Emergence of active nematic behavior in monolayers of isotropic cells. *Phys. Rev. Lett.* **122**, 048004 (2019). 30768306 10.1103/PhysRevLett.122.048004

[41] Basan, M., Elgeti, J., Hannezo, E., Rappel, W. J. & Levine, H. Alignment of cellular motility forces with tissue flow as a mechanism for efficient wound healing. *Proc. Natl. Acad. Sci. USA*. **110**, 2452 (2013). 23345440 10.1073/pnas.1219937110 PMC3574962

[42] Balasubramaniam, L. et al. Investigating the nature of active forces in tissues reveals how contractile cells can form extensile monolayers. *Nat. Mater.* **20**, 1156 (2021). 33603188 10.1038/s41563-021-00919-2 PMC7611436

[43] Zhang, G., Mueller, R., Doostmohammadi, A. & Yeomans, J. M. Active inter-cellular forces in collective cell motility. *J. R. Soc. Interface* **17**, 20200312 (2020). 32781933 10.1098/rsif.2020.0312 PMC7482557

[44] Thampi, S. P., Golestanian, R. & Yeomans, J. M. Active nematic materials with substrate friction. *Phys. Rev. E* **90**, 062307 (2014). 10.1103/PhysRevE.90.062307 25615093

[45] Ray, P. Statistical physics perspective of fracture in brittle and quasi-brittle materials. *Philos. Trans. R. Soc. A: Math., Phys. Eng. Sci.* **377**, 20170396 (2018). 10.1098/rsta.2017.0396 30478208

[46] Herrmann, H. J. & Roux, S. *Statistical models of fracture in disordered media* (North Holland, Amsterdam, 1990).

[47] Blow, M. L., Thampi, S. P. & Yeomans, J. M. Biphasic, lyotropic, active nematics. *Phys. Rev. Lett.* **113**, 248303 (2014). 25541809 10.1103/PhysRevLett.113.248303

[48] Serrano Nájera, G. & Weijer, C. J. Cellular processes driving gastrulation in the avian embryo. *Mech. Dev.* **163**, 103624 (2020). 32562871 10.1016/j.mod.2020.103624 PMC7511600

[49] Comba, A. et al. Spatiotemporal analysis of glioma heterogeneity reveals Col1a1 as an actionable target to disrupt tumor progression. *Nat. Commun.* **13**, 3606 (2022). 35750880 10.1038/s41467-022-31340-1 PMC9232499

[50] Lv, J.-Q. et al. Active hole formation in epithelioid tissues. *Nat. Phys.* **20**, 1313 (2024).

[51] McCord, M. & Notbohm, J. Measurement of tissue viscosity to relate force and motion in collective cell migration. *Soft Matter* **22**, 458 (2026). 41392955 10.1039/d5sm01139f PMC12771545

[52] Meacock, O. J., Doostmohammadi, A., Foster, K. R., Yeomans, J. M. & Durham, W. M. Bacteria solve the problem of crowding by moving slowly. *Nat. Phys.* **17**, 205 (2021).

[53] Maroudas-Sacks, Y. et al. Mechanical strain focusing at topological defect sites in regeneratingHydra. *Development* **152**, DEV204514 (2025). 40026208 10.1242/dev.204514 PMC11925399

[54] Copenhagen, K., Alert, R., Wingreen, N. S. & Shaevitz, J. W. Topological defects promote layer formation in myxococcus xanthus colonies. *Nat. Phys.* **17**, 211 (2021). 40893480 10.1038/s41567-020-01056-4 PMC12395370

[55] Vafa, F., Bowick, M. J., Shraiman, B. I. & Marchetti, M. C. Fluctuations can induce local nematic order and extensile stress in monolayers of motile cells. *Soft Matter* **17**, 3068 (2021). 33596291 10.1039/d0sm02027c

[56] Killeen, A., Bertrand, T. & Lee, C. F. Polar fluctuations lead to extensile nematic behavior in confluent tissues. *Phys. Rev. Lett.* **128**, 078001 (2022). 35244433 10.1103/PhysRevLett.128.078001

[57] Chen, Y. et al. Activation of topological defects induces a brittle-to-ductile transition in epithelial monolayers. *Phys. Rev. Lett.* **128**, 018101 (2022). 35061486 10.1103/PhysRevLett.128.018101

[58] Bonfanti, A., Duque, J., Kabla, A. & Charras, G. Fracture in living tissues. *Trends Cell Biol.* **32**, 537 (2022). 35190218 10.1016/j.tcb.2022.01.005

[59] Bidhendi, A. J., Lampron, O., Gosselin, F. P. & Geitmann, A. Cell geometry regulates tissue fracture. *Nat. Commun.* **14**, 8275 (2023). 38092784 10.1038/s41467-023-44075-4 PMC10719271

[60] Duque, J. et al. Rupture strength of living cell monolayers. *Nat. Mater.* **23**, 1563 (2024). 39468334 10.1038/s41563-024-02027-3 PMC11525174

[61] Papafilippou, E., Baldauf, L., Charras, G., Kabla, A. J. & Bonfanti, A. Interplay of damage and repair in the control of epithelial tissue integrity in response to cyclic loading. *Curr. Opin. Cell Biol.* **94**, 102511 (2025). 40233605 10.1016/j.ceb.2025.102511

[62] Martin, M. & Wood, M. J. Wound repair at a glance. *J. Cell Sci.* **122**, 3209 (2009). 19726630 10.1242/jcs.031187 PMC2736861

