# Supplementary material for: Multi-phase field model reveals internal dissipation is crucial for spontaneous hole formation in cell monolayers
Source: Nat Commun. 2026 Jul 3;17:8288. doi: 10.1038/s41467-026-74977-y (PMC13469578; doi:10.1038/s41467-026-74977-y)
Supplement: Supplementary file 1 [file 41467_2026_74977_MOESM10_ESM.pdf]

nature portfolio

Corresponding author(s): Julia M. Yeomans
Diogo E. P. Pinto

Last updated by author(s): Jun 11, 2026

Reporting Summary

Nature Portfolio wishes to improve the reproducibility of the work that we publish. This form provides structure for consistency and transparency in reporting. For further information on Nature Portfolio policies, see our Editorial Policies and the Editorial Policy Checklist.

Statistics

For all statistical analyses, confirm that the following items are present in the figure legend, table legend, main text, or Methods section.

n/a	Confirmed	
☐	☒	The exact sample size (n) for each experimental group/condition, given as a discrete number and unit of measurement
☐	☒	A statement on whether measurements were taken from distinct samples or whether the same sample was measured repeatedly
☐	☒	The statistical test(s) used AND whether they are one- or two-sided<br>*Only common tests should be described solely by name; describe more complex techniques in the Methods section.*
☐	☒	A description of all covariates tested
☐	☒	A description of any assumptions or corrections, such as tests of normality and adjustment for multiple comparisons
☐	☒	A full description of the statistical parameters including central tendency (e.g. means) or other basic estimates (e.g. regression coefficient) AND variation (e.g. standard deviation) or associated estimates of uncertainty (e.g. confidence intervals)
☐	☒	For null hypothesis testing, the test statistic (e.g. F, t, r) with confidence intervals, effect sizes, degrees of freedom and P value noted<br>*Give P values as exact values whenever suitable.*
☒	☐	For Bayesian analysis, information on the choice of priors and Markov chain Monte Carlo settings
☒	☐	For hierarchical and complex designs, identification of the appropriate level for tests and full reporting of outcomes
☐	☒	Estimates of effect sizes (e.g. Cohen's d, Pearson's r), indicating how they were calculated

Our web collection on statistics for biologists contains articles on many of the points above.

Software and code

Policy information about availability of computer code

Data collection	Data were generated using custom simulations of a two-dimensional active nematic multi-phase field model implemented in custom numerical code. Github repository: https://github.com/deppinto/RheoCell.
Data analysis	Data analysis and visualization were performed using custom analysis scripts and numerical post-processing routines. The equations of motion were integrated using finite-difference spatial discretization and a predictor–corrector time-stepping scheme, as described in the manuscript. Github repository: https://github.com/deppinto/RheoCell.

For manuscripts utilizing custom algorithms or software that are central to the research but not yet described in published literature, software must be made available to editors and reviewers. We strongly encourage code deposition in a community repository (e.g. GitHub). See the Nature Portfolio guidelines for submitting code & software for further information.

Data

Policy information about availability of data

All manuscripts must include a data availability statement. This statement should provide the following information, where applicable:

- Accession codes, unique identifiers, or web links for publicly available datasets
- A description of any restrictions on data availability
- For clinical datasets or third party data, please ensure that the statement adheres to our policy

Source data are provided with this paper. The initial configuration files, topology files, and input parameters used in this study have been deposited in the Zenodo database under the DOI 10.5281/zenodo.20591771.

Research involving human participants, their data, or biological material

Policy information about studies with human participants or human data. See also policy information about sex, gender (identity/presentation), and sexual orientation and race, ethnicity and racism.

Reporting on sex and gender	No human participants or human data was used for this work.
Reporting on race, ethnicity, or other socially relevant groupings	No human participants or human data was used for this work.
Population characteristics	No human participants or human data was used for this work.
Recruitment	No human participants or human data was used for this work.
Ethics oversight	No human participants or human data was used for this work.

Note that full information on the approval of the study protocol must also be provided in the manuscript.

Field-specific reporting

Please select the one below that is the best fit for your research. If you are not sure, read the appropriate sections before making your selection.

[x] Life sciences [ ] Behavioural & social sciences [ ] Ecological, evolutionary & environmental sciences

For a reference copy of the document with all sections, see nature.com/documents/nr-reporting-summary-flat.pdf

Life sciences study design

All studies must disclose on these points even when the disclosure is negative.

Sample size	No statistical methods were used to predetermine sample sizes. Sample sizes were chosen to ensure robust statistics across independent simulations and are consistent with previous computational studies of active nematic cell monolayers. Typical datasets consisted of averages over 10–100 independent simulations and 100 time frames, as specified in the figure captions.
Data exclusions	No simulations or data points were excluded unless explicitly stated in the manuscript. For hole statistics, lattice sites belonging to an already existing hole above the specified threshold size were excluded from further nucleation counting, as described in the main text.
Replication	All simulations were repeated across multiple independent initial conditions. Independent simulation runs reproduced the qualitative behavior reported in the manuscript, including spontaneous hole nucleation, velocity spirals, topological defects and the scaling relations.
Randomization	Cells were initialized at random positions in the simulation box before relaxation and production runs.
Blinding	No blinding was used because this was a computational modeling study.

Reporting for specific materials, systems and methods

We require information from authors about some types of materials, experimental systems and methods used in many studies. Here, indicate whether each material, system or method listed is relevant to your study. If you are not sure if a list item applies to your research, read the appropriate section before selecting a response.

Materials & experimental systems

n/a	Involved in the study	
[x]	[ ]	Antibodies
[x]	[ ]	Eukaryotic cell lines
[x]	[ ]	Palaeontology and archaeology
[x]	[ ]	Animals and other organisms
[x]	[ ]	Clinical data
[x]	[ ]	Dual use research of concern
[x]	[ ]	Plants

Methods

n/a	Involved in the study	
[x]	[ ]	ChIP-seq
[x]	[ ]	Flow cytometry
[x]	[ ]	MRI-based neuroimaging

Plants

Seed stocks	No plant material was used for this study.
Novel plant genotypes	No plant material was used for this study.
Authentication	No plant material was used for this study.
