# Supplementary material for: Multi-phase field model reveals internal dissipation is crucial for spontaneous hole formation in cell monolayers
Source: Nat Commun. 2026 Jul 3;17:8288. doi: 10.1038/s41467-026-74977-y (PMC13469578; doi:10.1038/s41467-026-74977-y)
Supplement: Supplementary file 2 [file 41467_2026_74977_MOESM11_ESM.pdf]

nature portfolio

# Peer Review File

# Multi-phase field model reveals internal dissipation is crucial for spontaneous hole formation in cell monolayers

Corresponding Author: Professor Julia Yeomans

**This file contains all reviewer reports in order by version, followed by all author rebuttals in order by version.**

Version 0:

Reviewer comments:

Reviewer #1

(Remarks to the Author)
This work studies computationally the generation of gaps in cell monolayers, using a previously developed many-body phase field model. Numerical results show that hole generation depends strongly on the magnitude of the cell-substrate friction coefficient, where low friction favors hole formation. The model includes a cell-shape-based nematic field: in the low friction regime, topological defects concentrate strains and stresses that may lead to tissue fracture.

Although the numerical results seem solid, the paper is poorly written (see below).
A thorough rewriting of the manuscript is necessary at this stage.

My first major comment concerns the model formulation.
This question is central to the interpretation of numerical results, and should be addressed appropriately.

While the manuscript claims to discuss the "effect of substrate friction" (parameter \xi_s), the text and figure captions present different values of the dimensionless parameter \xhi = \xi_s/\xi_cell, ratio of external over internal friction. Small \chi (as studied here) corresponds to cases where substrate friction is small _compared_ to internal dissipation!

To clarify the scope of this work, I would strongly recommend to first express of the governing equations ((1), (2), etc.) in dimensionless form, then define dimensionless parameters consistently, and finally vary these parameters!
If \xi_cell is taken as the unit for friction, the active parameter will certainly also be impacted!

In fact the time, length and force scales relevant for this problem are given by the authors (l. 105). Quick inspection shows that these order of magnitudes lead to a typical magnitude of the external friction \xi_s coefficient of 1, converting values typically estimated in experiments to the natural units of the problem.
Since \xi_cell is set to 1 in this work, I conclude that lower values of \chi (0.1 0.01 and 0.001) studied numerically are also lower values of \xi_s, which are therefore unphysical!

Surprisingly, the Authors write that the regime where \chi ~ 1 corresponds to substrate friction _dominating_ dissipation (e.g. l.110). This is contrary to common practice: substrate friction dominates dissipation when \chi \ll 1, a condition _not_ studied in this work.
When \chi ~ 1, internal and external friction contribute equally to dissipation.

Second, the Authors repeatedly mention the "elasticity" of the cell layer. However I could not find an elastic term in the equations that define the model. Perhaps the Authors really mean instead of "elasticity" the "surface tension" appearing in the Cahn-Hilliard free energy? How does this surface tension relate to an elastic behaviour of the material? Perhaps the Authors refer to previous work on this question? If so please give an explicit reference.
Overall, I am not sure what is meant by "elasticity" throughout.
For instance, l.126-128, I read: "Since the monolayer is compressible, if the active forcing and flows are large enough to overcome the elastic (passive) response that keeps the monolayer confluent, a hole can spontaneously form." I would rather expect that active forces need to be stronger than adhesive forces that maintain cell-cell cohesion in order to form a hole! (Holes usually form at cell-cell junctions in cell monolayers.)
However the computational model does not seem to include cell-cell adhesion (instead Eq. (S3) is a repulsion term between

cells), and indeed "cell-cell adhesion" is nowhere mentioned in the manuscript.

To be honest, I do not quite understand how hole formation in cohesive tissues can be studied without considering cell-cell adhesion explicitly in the context of a cell-based computation model.

Finally, the title should reflect the scientific content of the manuscript.
Like me, most colleagues in the field would expect hole formation in cell monolayers to result from collective organisation of the cells (as opposed to single cell behaviour). As a reader, I expect the title of an article to reflect its main novel result(s) rather than common expectations.
In addition, please mention explicitly in the title that the results are computational!

More comments:

- is the viscous term _necessary_ to observe hole formation?
If so this should be stated clearly from the beginning.
(In discussion I read: "Incorporating internal dissipation
into the multi-phase field approach used here has proven _essential_ to
generate the flow correlations [26, 27] needed to create holes.)

- previous work corresponds to \xi_cell = 0, hence \chi \to \infty.
Is this a singular limit of the current model?

- parameter choice should be justified

- l.145-156: is strain relevant? or is strain-rate relevant? or perhaps both?
Is there a well-defined reference state from which strains could be computed?

- most figures display ensemble averages (N = 100 typically) without showing error bars.
Please display error bars.

Minor comments:

- some figure labels are hard to read (e.g. Fig. 1cd, S2, S3)
Please use larger fonts.

- l. 223: "Previous work which considered this regime found small gaps in simulations of a multi-phase field model." Please include a reference.

- Ref. [26] and [31] are identical.

- SI A: please also give parameter values in standard units.

(Remarks on code availability)

Reviewer #2

(Remarks to the Author)
This paper presents a numerical study of how cell–substrate friction, cell–cell friction, and internal active dipolar forces combine to drive hole formation in a cell monolayer. The key finding is that decreasing substrate friction (relative to cell–cell friction) and increasing extensile dipolar activity enhance the likelihood of tissue rupture and hole emergence.

The modeling provides an insightful mechanistic framework for understanding ruptures and fractures in tissues, processes which are relevant to both development and disease. A particular strength of this study is the systematic exploration of the friction and activity parameter space, which is lacking in earlier works (e.g. Refs. 20 and 47). Nevertheless, the current presentation appears aimed mainly at the modeling community. The study does not include quantitative comparison with experiments, nor does it articulate clear, testable predictions for future validation.

Overall, the study is technically solid and makes a useful contribution to the modeling of active tissues. However, in its present form, the lack of experimental connection and predictive framing makes it less appealing to the broad readership of Nature Communications. I therefore do not recommend publication here, though the paper would be a strong fit for a more specialized journal in active matter or biophysical modeling. Below are some comments that may help the authors strengthen their work.

Major comments:
1. In Eq. (3), the passive force density is written as f_p = -\grad(\delta F/\delta\phi). I wonder if a factor of \phi is missing here before the gradient. In standard derivations of the thermodynamic force density from a free energy functional under a displacement field [see, e.g., Cates et al. J. Fluid Mech. 836, P1 (2018)], one obtains f_p = -\phi\grad( \delta F/\delta). Without this \phi prefactor the expression seems inconsistent with the variational derivation. Perhaps this is a typo, but if not, the authors should clarify the reasoning. While I expect the overall results are unlikely to change significantly, it would be useful to confirm and to validate that the model behavior is robust to this correction.

2. One of the main parameters varied in this paper is the hydrodynamic screening "length" \chi = \xi_s/\xi_{cell}. This definition is unfortunate, as it differs from the usual definition adopted in the continuum active matter literature [see, e.g., Thampi et al. Phys Rev. E 90, 062307 (2014) and Refs. 20 and 29], making direct comparison more difficult. In continuum models, the screening length is typically defined as \xi = \sqrt{\eta/\Gamma}, where \Gamma is the substrate friction and \eta is the viscosity (or the intercellular friction). Instead, the present definition corresponds more closely to 1/(screening length^2). [Here, \xi_{cell} carries the same dimensions as \xi_s because the discretized Laplacian omits the lattice spacing factor dx, effectively absorbing a unit dx^2 = 1.]

I strongly recommend redefining the screening length as \chi = \sqrt{\xi_{cell}/\xi_s}. This would align the notation with the existing literature, facilitate comparison across different models, and present more natural numerical values of \chi in Figs. 2 and 3.

3. In Fig. 2, the fact that P(hole|strain rate) follows an exponential function suggests that there is a critical strain rate required for holes to form. I think the authors can make this message more explicit, rather than just labelling this characteristic rate "the coefficient of the exponential fit" or the "slope" in the inset.

The authors suggest that this critical strain rate strongly depends on both friction \chi and activity \zeta by collapsing the fitted coefficients from different parameter sets, showing that they follow a master curve that scales with \chi^0.1. As it stands, the exponent 0.1 seems purely empirical, and it is difficult to interpret this result when \zeta still has dimensions that are different from those of the strain rate (1/[T]). I would suggest the authors conduct a proper dimensional analysis here – e.g., either rescale the strain rate and \zeta so they are dimensionless or construct a quantity from \chi, \zeta (and most likely the surface tension \gamma) so that it has the same dimensions as the strain rate. Understanding the relation between the critical strain rate and the model parameters properly would be a good prediction that can be tested in experiments and in other simulation models.

4. The paper's main outcome, whether a hole nucleates or not, is naturally controlled by two competing length scales: the hydrodynamic screening length l_h = \sqrt{\xi_{cell}/\xi_s} (flows screened by substrate friction) and the active length scale l_a = \sqrt{\gamma/(|\zeta|\lambda)} (activity vs elasticity; nondimensionalized by dividing by \lambda). It would be informative to test whether the 2D phase diagram in Fig. 3d collapses onto a single curve when plotted against the dimensionless ratio l_h/l_a. If hole formation is controlled by the competition between screening and activity, one expects a threshold value of l_h/l_a separating the no-hole and hole regimes. Collapsing the data in this way would greatly clarify the physics and make the results directly comparable to existing active-matter theory. Also, in Figs. 3a and 3b, do the curves for the hole area and number of holes collapse (or obey some kind of scaling) when plotted against the ratio l_h/l_a?

5. The manuscript mostly reports whether holes form or not (e.g., Fig. 3d), but it would be valuable to also quantify how they form and evolve. In particular, for individual holes, what is the typical growth or closure law of the hole area? Including such statistics, along with representative simulation movies that show nucleation, growth, and closure (with overlays of velocity or nematic fields) would make the results much clearer and easier to connect with experiments.

6. The relatively small system size used by the authors seems to only permit one or two holes to form (if I read Fig. 3b correctly). Given this, can the author provide some evidence that their results are not strongly affected by boundary effects? I understand that multiphase field simulations are computationally expensive, but I believe it is worth validating the results with a larger system size.

Minor comments:
7. In line 110, Refs. 35-37 are cited for their relevance to the concept of the hydrodynamic screening length \chi. Yet, upon looking at these references, I am unable to find Refs. 35 and 37 discussing this metric. I suggest that the authors should cite other papers that explicitly mention this metric (see those in point 2 above).

8. The correlation functions for v and Q in the Supplementary Information Section B should be labelled as Eqs. S5 and S6 to avoid reusing the same labels for the model equations given in Section A.

(Remarks on code availability)
The code repository still appears to be in a work-in-progress state, with no clear instructions on how to compile and run the code (the README file just says that the repository is under construction). As such, I was unable to install the program to test it.

Version 1:

Reviewer comments:

Reviewer #1

(Remarks to the Author)
Unfortunately, I find that the resubmitted manuscript is still lacking on two major issues:
- relevance to experiments
- dimensional analysis
As a consequence, my opinion is that this work is not fit for publication in Nature Communications.

1. Relevance to experiments:

This work was motivated by ref. [20].
In [20], I read:
"A closer examination revealed two types of cellular event prior to hole initiation: ~10 ± 3.2% of holes were associated with breakage of junctions following stretching of a cell (Extended Data Fig. 1c and Supplementary Video 4), and ~90 ± 4.1% were formed at cell-division sites during abscission"

Since the model does not include cell division among its ingredients, its predictions pertain to only one in ten hole formation events observed experimentally in [20].

I read, l.325-326:
"The cell monolayer experiments suggest that cell divisions also play a relevant role in hole formation [20]."
Please correct this statement.
According to the experiment, cell division plays the dominant role in hole formation.

In addition, the experimental average velocity field close to hole formation events is given in Extended Fig. 4b of Ref. [20}.
The pattern differs markedly from the spiral shown in Fig. 1b of this work.

Finally, there is no mention in Ref. [20] of defect pairing prior to hole formation.

I conclude that the Authors' predictions disagree with current experimental observations.
This fact should be stated explicitly in the manuscript.

2. Elementary dimensional analysis:

It remains unclear throughout the manuscript whether parameters and fields are dimensionful or dimensionless.

- According to Eq. (2), \xi_cell and \xi_s do not have the same unit. Indeed their ratio allows to define the square of the hydrodynamic length.
I read p. 13, l. 288:
"In the friction dominated regime \xi_cell |le \xi_s..."
Since units differ, this inequality does not make sense.

- Since \xi_cell and \xi_s do not have the same unit, I would use different symbols for each. Since \xi_cell is, in fact, a viscosity, not a friction coefficient, why not simply call it \eta instead of \xi_cell? This is the standard practice in the field.

- In Eq. (7), the factor l_h^2 L^2 / \delta \bar{x}^2 cannot be dimensionless.

- l. 139 and 143, inequalities such as l_h \gg 1 do not make sense, since l_h (without a \bar{}) is a length.

- The reference timescale T has not been defined.
For consistency, I would suggest to use \tau_F as a reference timescale.

I strongly recommend that the Authors proofread their manuscript and check that their results are everywhere consistent with dimensional analysis. For clarity, dimensionless quantities should always carry a \bar{}.

Other comments:

1. The new title emphasizes the role plaid by internal dissipation in hole formation in this model. This point should also be mentionned in the abstract.
Since this is the main result of this work, this result should be illustrated explicitly, and perhaps Section B of the supplementary Material should be moved to the main text. I would also suggest adding a figure similar to Figs. S3b and S4b, where the hole density would be plotted as a function of the viscosity.

2. I would move Sec. A from SI to main text.

In the present manuscript, important parameters such as \gamma (l. 110), or the surface tension (l.175) appear in the text without having been defined first.

3. Wherever possible, "strain rate" should be replaced by "isotropic strain rate" for clarity.
One naturally wonders whether large values of the shear strain rate also lead to hole formation in this model.

4. It would be interesting to plot the strain _and_ (isotropic) strain rate fields close to hole formation events. Since the model comes with a well-defined reference area (Eq. S2), the strain can also be evaluated without ambiguity.

5. l. 281: strain -> strain rate
(unless a characteristic strain can be defined)

6. Since the function S is fitted by an exponential, Fig 4d should be plotted in lin-log scale, instead of lin-lin scale.

Minor comments:

- in Eq, (5) the integration variable is d \vec{x} not dx

- l. 297: epithiloid -> epithelioid

(Remarks on code availability)

Reviewer #2

(Remarks to the Author)
The authors have done substantial revisions to the manuscript, and I appreciate that they have taken the time to address the concerns I had raised. Overall, I find the revised manuscript much strengthened scientifically: the new presentation of the non-dimensional friction parameter F = l_h/l_a, as suggested in the first round of review, captures well the physics of hole formation in this system. It yields new scaling relations and exponents (e.g., the characteristic strain rate scales with F^{-3}) that can be tested in other active soft matter systems. The new result that cell alignment along the hole interface depends on hole growth or shrinkage provides additional tangible predictions that can be validated in experiments.

I still have a few minor comments that I would like the authors to address before recommending publication:

1. Regarding my original concern about the passive force term (major point 1), I would recommend the authors stating clearly in the manuscript (either in the main text or in the SI) the reason for choosing to simulate this force term as f^p = -\grad (\delta F/\delta\phi), as it differs from what they have done previously and from variational derivation.

2. In the non-dimensionalized force balance equation (Eq. 7), I believe there is a typo: L^2 should be in the denominator in the intercellular friction term.

3. The authors should state the p-value for the Pearson correlation between the cell alignment along the hole interface and the rate of change of the hole area, as shown in Fig. 3a.

4. Please also state the parameter values used for the simulation shown in the supplemental videos. In Video S4, which shows the active force density field, it seems that horizontal and vertical bands appear throughout the video. Can the authors confirm that these are not artifacts due to simulating the phase fields in subdomains?

5. There are some typos in the SI:
- In the caption of Fig. S3, the old parameter \chi is still reported; the author should convert that to the new parameter l_h.
- In Section F, Figure S6 should be Figure S5.

(Remarks on code availability)
While I was able to compile the code following the instructions in the README, I was unable to run the program, getting errors saying that the configuration file cannot be read. The authors should provide more instructions on how the configuration and parameter files should be specified, as well as a minimal working example.

**Open Access** This Peer Review File is licensed under a Creative Commons Attribution 4.0 International License, which permits use, sharing, adaptation, distribution and reproduction in any medium or format, as long as you give appropriate credit to the original author(s) and the source, provide a link to the Creative Commons license, and indicate if changes were made.
In cases where reviewers are anonymous, credit should be given to 'Anonymous Referee' and the source.
The images or other third party material in this Peer Review File are included in the article's Creative Commons license, unless indicated otherwise in a credit line to the material. If material is not included in the article's Creative Commons license and your intended use is not permitted by statutory regulation or exceeds the permitted use, you will need to obtain permission directly from the copyright holder.
To view a copy of this license, visit https://creativecommons.org/licenses/by/4.0/

## Response to Referee 1

We would like to thank the Referee for carefully reading our manuscript and helping us improve our work.

**This work studies computationally the generation of gaps in cell monolayers, using a previously developed many-body phase field model. Numerical results show that hole generation depends strongly on the magnitude of the cell-substrate friction coefficient, where low friction favors hole formation. The model includes a cell-shape-based nematic field: in the low friction regime, topological defects concentrate strains and stresses that may lead to tissue fracture.**
**Although the numerical results seem solid, the paper is poorly written (see below). A thorough rewriting of the manuscript is necessary at this stage.**

We thank the Referee for the constructive comments and appreciate the suggestions provided. Below, we offer detailed responses to each point.

**My first major comment concerns the model formulation. This question is central to the interpretation of numerical results, and should be addressed appropriately.**
**While the manuscript claims to discuss the "effect of substrate friction" (parameter $\xi_s$), the text and figure captions present different values of the dimensionless parameter $\chi = \xi_s/\xi_{cell}$, ratio of external over internal friction. Small $\chi$ (as studied here) corresponds to cases where substrate friction is small compared to internal dissipation!**
**To clarify the scope of this work, I would strongly recommend to first express of the governing equations ((1), (2), etc.) in dimensionless form, then define dimensionless parameters consistently, and finally vary these parameters! If $\xi_{cell}$ is taken as the unit for friction, the active parameter will certainly also be impacted!**

We agree with the Referee's suggestions and have added further details to the Model section to include the non-dimensionalization of the model and introduce the non-dimensional parameters now used to present the results.
In particular, we introduce two relevant parameters, in line with active nematic theories (Doostmohammadi, 2018): the hydrodynamic length scale, $l_h = \sqrt{\xi_{cell}/\xi_s}$, which quantifies how hydrodynamic-like effects arising from cell–cell friction are screened by substrate friction, and an active length scale, $l_a = \sqrt{\gamma\lambda/|\zeta|}$, which relates active forces to elasticity. Their ratio, $F = l_h/l_a = \sqrt{\xi_{cell}\zeta/\gamma\lambda\xi_s}$, defines the key non-dimensional parameter. Expressing the measured quantities as a function of this non-dimensional friction number allowed us to collapse data for different parameters and extract the corresponding scaling relations.

We have made substantial revisions to both the text and the figures to emphasize the role of this parameter. In particular, we have added the following details to the Model section:

*One can non-dimensionalise the force balance equation, Eq. (2), by introducing the characteristic length and time scales, L and T respectively. This leads to the following dimensionless variables:*

$$\tilde{\boldsymbol{x}} = \frac{\boldsymbol{x}}{L}, \tilde{t} = \frac{t}{T}, \tilde{\boldsymbol{v}} = \frac{\boldsymbol{v}}{V}, \tag{1}$$

*where $V = L/T$ is the velocity scale. We can introduce these variables in Eq. (2) and reach the following non-dimensionalised equation:*

$$\tilde{\boldsymbol{v}}_i(\tilde{\boldsymbol{x}}) + \frac{l_h^2 L^2}{\Delta\tilde{x}^2} \sum_{\boldsymbol{x}' \in \mathcal{N}(\boldsymbol{x})} \sum_{j \in \boldsymbol{x}'} [\tilde{\boldsymbol{v}}_i(\tilde{\boldsymbol{x}}) - \tilde{\boldsymbol{v}}_j(\tilde{\boldsymbol{x}}')] = \boldsymbol{f}_i^p(\tilde{\boldsymbol{x}})/\xi_s V - A\boldsymbol{Q}_i \cdot \tilde{\nabla}\phi_i(\tilde{\boldsymbol{x}}), \tag{2}$$

*where $l_h = \sqrt{\xi_{cell}/\xi_s}$ and $A = \zeta T/\xi_s L^2$. From this equation we can identify one of the relevant length scales, the hydrodynamic length scale, $l_h$, which sets the typical distance over which hydrodynamic-like effects (coming from cell-cell friction) are relevant. For the passive energy term, we consider, to first order, that the main contribution for the passive restoring force comes from the $\gamma\lambda\nabla^2\phi$ term in the Cahn-Hilliard free energy, representing a penalty for distortions in the shape of the cells. We then introduce an active length scale $l_a = \sqrt{\gamma\lambda/|\zeta|}$, that denotes the characteristic length scale where active stresses overcome passive restoring forces. Combining these two length scales, we define the non-dimensional friction number, $F = l_h/l_a$ [36].*

and the following to the Discussion section:

*We introduce a non-dimensional friction number, F, which allows us to collapse the data and reveals the relevant scaling relations between both the characteristic strain for hole nucleation and the typical hole lifetime and the friction number. This scaling highlights the key length scales governing hole formation and dynamics, in particular the hydrodynamic and active length scales.*

**In fact the time, length and force scales relevant for this problem are given by the authors (l. 105). Quick inspection shows that these order of magnitudes lead to a typical magnitude of the external friction $\xi_s$ coefficient of 1, converting values typically estimated in experiments to the natural units of the problem. Since $\xi_{cell}$ is set to 1 in this work, I conclude that lower values of $\chi$ (0.1 0.01 and 0.001) studied numerically are also lower values of $\xi_s$, which are therefore unphysical!**

There are instances in living systems where cell-cell friction is dominant over cell-substrate friction, and the latter may even be negligible. For example, during avian gastrulation, the tissue is suspended on a fluid rather than in contact with a substrate (so $\xi_s \sim 0$), but cells still move on scales much larger than a single cell (Serrano 2020). Moreover, the case where friction dominates over viscosity corresponds to very short-ranged correlations due to the absence of hydrodynamic effects. However, multiple experiments on MDCK monolayers

(and other cell types) show how correlations can extend to distances much larger than the individual cell size (Petitjean, 2010). Moreover, different modes of dissipation play a role during this collective motion. In particular, viscosity, which we describe with the cell-cell friction term, has been highlighted as a key component to explain multiple biological functions related to collective motion (McCord, 2025). Therefore, we consider it physically reasonable to explore regimes in which substrate friction is much smaller relative to tissue viscosity, which we include as an intercellular friction term.

**Surprisingly, the Authors write that the regime where $\chi \sim 1$ corresponds to substrate friction dominating dissipation (e.g. l.110). This is contrary to common practice: substrate friction dominates dissipation when $\chi \ll 1$, a condition not studied in this work. When $\chi \sim 1$, internal and external friction contribute equally to dissipation.**

We acknowledge that when substrate and cell–cell friction are of the same order of magnitude, $\xi_s \sim \xi_{cell}$, they contribute with comparable weight to the equations. Nonetheless, within the framework of the *dry* and *wet* dynamics presented in the Supplementary Information, the regimes $\xi_s \sim \xi_{cell}$ and $\xi_s \ll \xi_{cell}$ remain distinct. We observe that in the former ($\xi_s \sim \xi_{cell}$), the correlations between cells are short-range, in the sense that they are smaller or comparable to the typical size of the cell. Thus, the dynamics will be comparable to a *dry* system. In contrast, in the latter case ($\xi_s \ll \xi_{cell}$), correlations extend to length scales larger than the typical cell size and the dynamics are described by a more coherent motion among cells. This is similar to a *wet* regime.

In this work, we introduce internal dissipation into the multiphase-field model to show that it is a key ingredient for hole formation. Thus, in the context of cell collective motion, the tissue must be in the *wet* regime for holes to nucleate.

To clarify this point, we have rewritten the first paragraph of the Results section as follows:

*To focus on the effects of substrate friction, cell-cell friction and activity on the collective behaviour of cell monolayers we use the non-dimensional friction number, $F = l_h/l_a$, as a measure of the ratio between the hydrodynamic screening length and the active length scale [36, 44]. This parameter emphasises the competition between the relevant forces in the system. In particular, for the hydrodynamic screening length, we observe that when $l_h \lesssim 1$, the dynamics of the system is similar to the pure substrate friction case ($\xi_{cell} = 0$), as the characteristic length scale of velocity and nematic correlations is smaller than the typical cell size (see Supplementary Information Sec. B). In this regime, the motion of the cells is random, and no holes form in the monolayer, for any value of the active length scale, $l_a$. When the substrate friction is sufficiently low ($l_h \gg 1$) that the characteristic length scale of correlations is larger than the cell size (see Supplementary Material Sec. B), and the activity is large enough to compete with the elastic restoring force of the monolayer, the system enters a regime similar to active turbulence [21], and holes spontaneously form. This observation underscores how cell-cell friction can be a key physical ingredient to accurately realize monolayer collective behaviour.*

**Second, the Authors repeatedly mention the "elasticity" of the cell layer.**

**However I could not find an elastic term in the equations that define the model. Perhaps the Authors really mean instead of "elasticity" the "surface tension" appearing in the Cahn-Hilliard free energy? How does this surface tension relate to an elastic behaviour of the material? Perhaps the Authors refer to previous work on this question? If so please give an explicit reference. Overall, I am not sure what is meant by "elasticity" throughout. For instance, l.126-128, I read: "Since the monolayer is compressible, if the active forcing and flows are large enough to overcome the elastic (passive) response that keeps the monolayer confluent, a hole can spontaneously form." I would rather expect that active forces need to be stronger than adhesive forces that maintain cell-cell cohesion in order to form a hole! (Holes usually form at cell-cell junctions in cell monolayers.)**

In our case, we start from the confluent regime, in which cells are tightly packed. This implies that when the system is deformed, it responds elastically in the small-deformation limit. This behaviour arises because the phase-field model includes a repulsion term between cells and an area constraint/surface tension that maintains a preferred shape. A similar effect can be observed in dry granular systems or dense colloidal suspensions which, despite having no adhesion between particles, still display an elastic response under small deformations (Bonn, 2017).

We note, however, that this is not elasticity in the sense of standard continuum elastic models (e.g. Maxwell-type models), as particle-based systems can yield and deform plastically once sufficient strain is applied. At the single-cell level, elasticity emerges directly from the free energy: the soft area constraint essentially acts as a spring, keeping the cell area finite and close to a preferred value.

Our intention was to highlight how the passive forces oppose hole formation, effectively acting as an elastic restoring force. Creating a hole is penalized by the free energy, since cell areas must be reduced and cell shapes must be distorted to accommodate a void in the tissue. The degree to which cells can deform is therefore one of the relevant physical scales influencing hole formation. We emphasize that deriving a general continuum theory of elasticity from the individual free-energy parameters is extremely challenging and lies beyond the scope of this work. Accordingly, we restrict our analysis to the effects of activity and friction (substrate and intercellular), and incorporate a simplified representation of elasticity into our non-dimensional friction number, $F = \sqrt{\xi_{cell}|\zeta|/\gamma\lambda\xi_s}$, through the term $\gamma\lambda$, which sets the energy scale for shape deformation. To make this reasoning explicit, we have added the following discussion to the Model section:

*For the passive energy term, we consider, to first order, that the main contribution for the passive restoring force comes from the $\gamma\lambda\nabla^2\phi$ term in the Cahn-Hilliard free energy, representing a penalty for distortions in the shape of the cells. We then introduce an active length scale $l_a = \sqrt{\gamma\lambda/|\zeta|}$, that denotes the characteristic length scale where active stresses overcome passive restoring forces.*

**However the computational model does not seem to include cell-cell adhesion (instead Eq. (S3) is a repulsion term between cells), and indeed "cell-cell**

**adhesion" is nowhere mentioned in the manuscript.**
**To be honest, I do not quite understand how hole formation in cohesive tissues can be studied without considering cell-cell adhesion explicitly in the context of a cell-based computation model.**

To clarify this point, we have added a new section to the Supplementary Information in which we describe how to include a cell–cell adhesion term to the model. We also added new simulation results showing how adhesion influences hole formation. We observe that increasing adhesion enhances the average hole area, as cells tend to stick to each other more, producing an effect similar to that of strengthening correlations through the viscous term. Given its relatively minor impact, and in order to keep an already complex modelling approach as clear as possible, we present this analysis only in the Supplementary Information and include a reference in the main text.

This behaviour goes in line to what is seen in similar studies (Chiang, 2024), where adhesion has a minimal to moderate effect on the dynamics up to a certain point. It effectively increases correlations between cells, a role that in our case is already captured via the viscous term. Only at high adhesion does the behaviour change substantially: the tissue becomes jammed, and in this state no motion occurs and holes cannot form.

We note that in our model confluency arises from the absence of gaps between cells, enforced by the constraint of the simulation box size. This confers elastic properties to the tissue, effectively keeping cells close together. We also remark that the cell–cell friction introduced here acts as a viscous term, or as a mechanism for momentum transport between neighbouring cells. Such interactions may arise from various processes at cell junctions, including cell–cell adhesion. Recent work has shown that tuning adhesion, for example, by cytochalasin D treatment, which reduces the relative intensity of E-cadherin at cell–cell junctions, can modulate tissue viscosity (McCord, 2025). In this work, however, we simplify this interaction by considering only its viscous contribution.

We have added the following line to the Discussion:

*In Sec. D of the Supplementary Information we corroborate this by showing that when we increase the elasticity of the monolayer (through the surface tension of the cells) the probability of nucleating holes decreases and in Sec. E of the Supplementary Information we explore how an explicit cell-cell adhesion term can affect the hole formation.*

and the following text to the Supplementary Information:

*Effects of an adhesion term in the free energy. As in previous work [2], we introduce a free-energy term that mimics cell–cell adhesion:*

$$\mathscr{F}_{adh} = \sum_i \sum_{j \neq i} \omega\lambda \int d\mathbf{x} \nabla\phi_i^2(\mathbf{x}) \nabla\phi_j^2(\mathbf{x}). \tag{3}$$

*In Fig. S4, we show the average maximum area and average number of holes as a function of cell–cell adhesion. We observe that the results mirror the ones shown in Fig. 3b and*

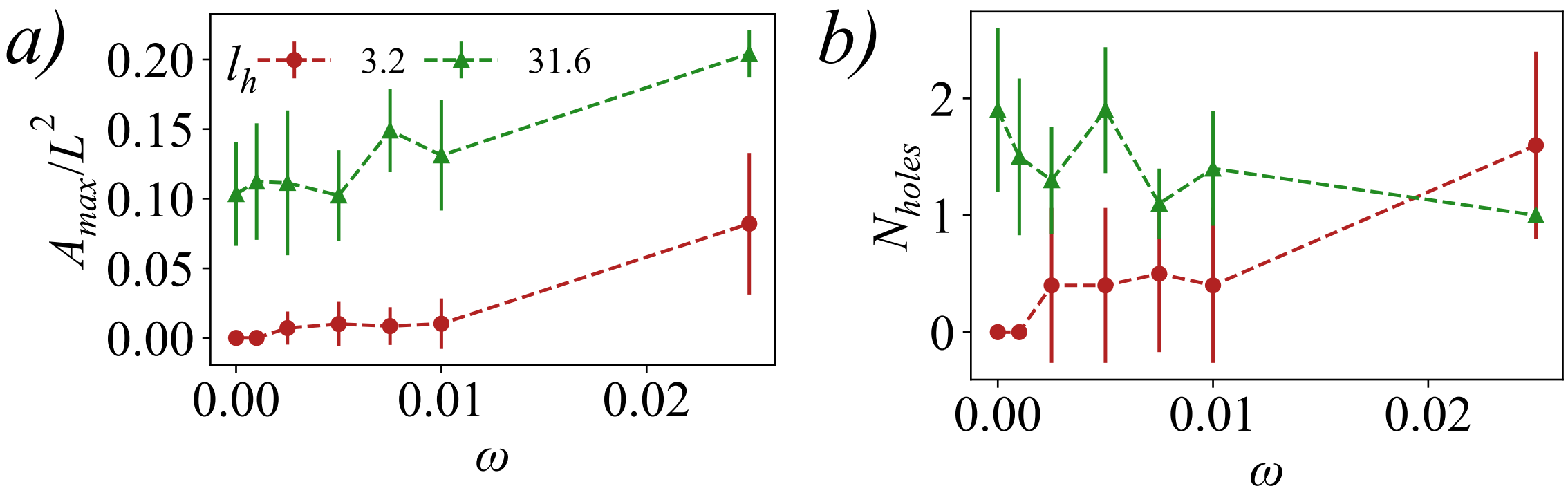


Fig. S4: *Maximum area and total number of holes (a and b respectively), as a function of adhesion, for* $l_h = [3.2, 31.6]$ *and* $\zeta = 0.3$. *These results were averaged over* 10 *trajectories with* 100 *time-frames each.*

*c of the main text. This occurs because cells adhere more strongly with increasing* $\omega$, *thereby increasing correlations [6]. In this sense, adhesion has an effect similar to reducing substrate friction (from the perspective of correlation length), which enhances the likelihood of hole formation. If adhesion becomes sufficiently large, the monolayer enters a jammed state. In this regime, small gaps may appear, as reported in previous work [11], but these arise from packing constraints rather than from cell dynamics. Moreover, the typical size of these gaps is on the order of, or smaller than, the cell size, suggesting that no nucleation process underlies these events.*

**Finally, the title should reflect the scientific content of the manuscript. Like me, most colleagues in the field would expect hole formation in cell monolayers to result from collective organisation of the cells (as opposed to single cell behaviour). As a reader, I expect the title of an article to reflect its main novel result(s) rather than common expectations. In addition, please mention explicitly in the title that the results are computational!**

Following the Referee's advice, we have changed the tittle of the manuscript to: "Multiphase field model reveals internal dissipation is crucial for hole formation in cell monolayers"

**More comments:**
**- is the viscous term necessary to observe hole formation? If so this should be stated clearly from the beginning. (In discussion I read: "Incorporating internal dissipation into the multi-phase field approach used here has proven essential to generate the flow correlations [26, 27] needed to create holes.)**

The viscous term is a necessary component for hole formation in the tissue. Without it (or in the substrate-friction–dominated regime), holes do not form. To make this point explicit, we have added the following clarification to the first paragraph of the Results section:

*In particular, for the hydrodynamic screening length, we observe that when* $l_h \lesssim 1$, *the*

*dynamics of the system is similar to the pure substrate friction case ($\xi_{cell} = 0$), as the characteristic length scale of velocity and nematic correlations is smaller than the typical cell size (see Supplementary Information Sec. B). In this regime, the motion of the cells is random, and no holes form in the monolayer, for any value of the active length scale, $l_a$.*

**- previous work corresponds to $\xi_{cell} = 0$, hence $\chi \to \infty$. Is this a singular limit of the current model?**

This is indeed one of the limits of the model, but it is not a singular one; rather, it corresponds to a well-defined limit where the internal dissipation term does not contribute to the dynamics. A model where $\xi_{cell} = 0$ always exhibits dynamics corresponding to the *dry* regime. In fact, in Supplementary Information Sec. B, we compare this limit to the case with $\xi_{cell} > 0$ by examining the velocity and nematic director correlation functions.

**- parameter choice should be justified**

Our parameter choices follow those used in previous work (Zhang, 2023), which were selected based on two relevant criteria: First, when converted to physical units, they fall under acceptable ranges for the systems we are trying to describe (as seen in Table 1 in the Supplementary Information); Second, they optimize the stability of the model, ensuring that the dynamics remain within physically relevant regimes, namely, that cells are neither too stiff nor too soft. We have added the following text to the Supplementary Information Sec. A:

*Similar to previous work [2] and to keep the physical values within acceptable ranges (see Table S1), we chose the following parameter values: $\lambda = 2.0$, $\gamma = 0.06$, $\kappa = 0.5$, $\mu = 20$, and $J_0 = 1$.*

**- l.145-156: is strain relevant? or is strain-rate relevant? or perhaps both? Is there a well-defined reference state from which strains could be computed?**

We argue that both quantities are important; however, the strain rate is more readily accessible, as it can be computed directly from the velocity field. In contrast, determining the strain would require modelling deformations relative to a well-defined reference state, which is extremely challenging

**- most figures display ensemble averages (N = 100 typically) without showing error bars. Please display error bars.**

We have added error bars to the plots; in some cases, the error bars are smaller than the marker size and, therefore, not visible.

**Minor comments:**

**- some figure labels are hard to read (e.g. Fig. 1cd, S2, S3) Please use larger fonts.**

We once again thank the Referee for this suggestion. The figures have been updated to improve their readability.

**- l. 223: "Previous work which considered this regime found small gaps in simulations of a multi-phase field model." Please include a reference.**

We have added the relevant reference:

[37] A. Ardaševa, R. Mueller, and A. Doostmohammadi, Bridging microscopic cell dynamics to nematohydrodynamics of cell monolayers, Soft Matter 18, 4737 (2022).

It now reads:

*Previous work which considered this regime found small gaps in simulations of a multi-phase field model [37].*

**- Ref. [26] and [31] are identical.**

We have corrected the references. They now read:

[26] C. Fu, F. Dilasser, S.-Z. Lin, M. Karnat, A. Arora, H. Rajendiran, H. T. Ong, N. M. H. Brenda, S. W. Phow, T. Hirashima, M. Sheetz, J.-F. Rupprecht, S. Tlili, and V. Viasnoff, Regulation of intercellular viscosity by E-cadherin-dependent phosphorylation of EGFR in collective cell migration, Proceedings of the National Academy of Sciences of the United States of America **121**, e2405560121 (2024).

[31] S. C. Kammeraat, Y.-E. Keta, P. Appleton, I. P. Newton, T. B. Liverpool, R. Sknepnek, I. Näthke, and S. Henkes, Correlated cell movements drive epithelial finger formation (2025), arXiv:2508.01046 [cond-mat.soft].

**- SI A: please also give parameter values in standard units.**

We have added a table to the Supplementary Information Sec. A that lists the relevant parameter ranges in standard units. Specifically, we have included the following:

*Based on typical values for MDCK cells, such as an average radius of approximately* $10\mu m$*, velocity around* $20\mu m/h$*, and pressure on the order of* $100\ Pa$*, as measured via Particle Image Velocimetry and Traction Force Microscopy [3], we estimate that the simulation units correspond to physical units of* $\Delta x \sim 1\ \mu m$*,* $\Delta t \sim 5\ s$*, and* $\Delta F \sim 1.5\ nN$ *for length, time, and force, respectively [2]. The rest of the parameters are summarized in Table S1.*

| Parameter | Interpretation | Dimensions | Value(s) in simulation units | Value(s) in physical units |
|---|---|---|---|---|
| $\Delta x$ | Size of each lattice site | $[L]$ | 1 | 1 $\mu$m |
| $\Delta t$ | Size of each timestep | $[T]$ | 1 | 5 s |
| $\Delta F$ | Force scale | $[F]$ | 1 | 1.5 nN |
| $\gamma$ | Surface tension | $[F]$ | 0.06 | 0.09 nN |
| $\lambda$ | Interfacial thickness | $[L]$ | 2 | 2 $\mu$m |
| $\mu$ | Area constraint | $[F][L]$ | 20 | 30 nN·$\mu$m |
| $\kappa$ | Cell–cell repulsion | $[F]$ | 0.5 | 0.75 nN |
| $R$ | Ideal cell radius | $[L]$ | 8 | 8 $\mu$m |
| $\xi_s$ | Cell–substrate friction | $[F][T][L]^{-3}$ | 0.001 - 1.0 | 0.0075 - 7.5 kPa·s/$\mu$m |
| $\xi_{cell}$ | Cell–cell friction | $[F][T][L]^{-1}$ | 0.1 - 1.0 | 0.75 - 7.5 MPa·s·$\mu$m |
| $\zeta$ | Active stress | $[F][L]^{-1}$ | 0.1 - 0.5 | 0.15 - 0.75 nN/$\mu$m |

**Table S1.** A full list of the model parameters, their dimensions, and their values both in simulation and physical units.

## Response to Referee 2

We would like to thank the Referee for their careful reading of our manuscript, and for helping us to improve our work.

**This paper presents a numerical study of how cell–substrate friction, cell–cell friction, and internal active dipolar forces combine to drive hole formation in a cell monolayer. The key finding is that decreasing substrate friction (relative to cell–cell friction) and increasing extensile dipolar activity enhance the likelihood of tissue rupture and hole emergence.**
**The modeling provides an insightful mechanistic framework for understanding ruptures and fractures in tissues, processes which are relevant to both development and disease. A particular strength of this study is the systematic exploration of the friction and activity parameter space, which is lacking in earlier works (e.g. Refs. 20 and 47). Nevertheless, the current presentation appears aimed mainly at the modeling community. The study does not include quantitative comparison with experiments, nor does it articulate clear, testable predictions for future validation.**
**Overall, the study is technically solid and makes a useful contribution to the modeling of active tissues. However, in its present form, the lack of experimental connection and predictive framing makes it less appealing to the broad readership of Nature Communications. I therefore do not recommend publication here, though the paper would be a strong fit for a more specialized journal in active matter or biophysical modeling. Below are some comments that may help the authors strengthen their work.**

We thank the Referee for the thoughtful review and the positive assessment of our results. We have carefully considered all suggestions and substantially revised the manuscript to improve clarity and broaden its appeal. In particular, we have introduced a non-dimensional friction number, which enables a clear data collapse and the extraction of scaling behavior.

We have also added new results concerning the dynamics of hole formation and evolution. Specifically, when cells align tangentially to the hole boundary, the hole is stabilized or grows, whereas alignment perpendicular to the boundary promotes hole shrinkage. We believe that these new insights, together with our previous findings, significantly strengthen the manuscript and enhance its relevance to a broader audience.

From an experimental perspective, our results suggest that the presence of two $+1/2$ defects is necessary for hole formation, with nucleation occurring near a spiral in the velocity field. Furthermore, sustained hole growth requires cells to be aligned along the tangential direction of the hole. Both predictions are directly testable in experiments.

On the theoretical side, the observed data collapse and scaling behavior provide concrete benchmarks for continuum theories that incorporate compressible flows. We therefore

believe that the revised manuscript will engage a wider readership and help stimulate further experimental and theoretical studies.

**Major comments: 1. In Eq. (3), the passive force density is written as $f_p = -\nabla(\delta F/\delta\phi)$. I wonder if a factor of $\phi$ is missing here before the gradient. In standard derivations of the thermodynamic force density from a free energy functional under a displacement field [see, e.g., Cates et al. J. Fluid Mech. 836, P1 (2018)], one obtains $f_p = -\phi\nabla(\delta F/\delta)$. Without this $\phi$ prefactor the expression seems inconsistent with the variational derivation. Perhaps this is a typo, but if not, the authors should clarify the reasoning. While I expect the overall results are unlikely to change significantly, it would be useful to confirm and to validate that the model behavior is robust to this correction.**

In previous versions of our model, the passive force density was defined as $f_p = -(\delta F/\delta\phi)\nabla\phi$ (see (Mueller, 2019) and (Zhang, 2020)). Under periodic boundary conditions, however, we observed that the total passive force, when summed over all cells and lattice points, did not vanish. In the friction-dominated regime, this issue has little effect because correlations are short-ranged and the contribution from the residual net force is negligible. In contrast, in the viscosity-dominated regime, where substrate friction is very small, this non-zero passive force leads to an unphysical unidirectional flow of the entire system. To resolve this, we have modified the definition of the passive force to $f_p = -\nabla(\delta F/\delta\phi)$, which ensures that the total passive force sums to zero.

We are still investigating a more rigorous formulation of the passive force that remains consistent with previous derivations while satisfying the zero-sum condition under periodic boundaries. The precise reason why $f_p = -(\delta F/\delta\phi)\nabla\phi$ fails to sum to zero is not yet fully understood, though it may arise from numerical artifacts. In particular, each phase field is resolved only within a finite subdomain of the simulation box, a necessary simplification to keep the model computationally tractable.

Importantly, the analytical form of the passive force is not critical, provided it drives the system toward the minimum of the free energy, which our revised formulation continues to do. The key requirement is that the total force vanishes across the system; otherwise, an unphysical spontaneous flow can emerge at low substrate friction, even in the absence of external forces or activity.

**2. One of the main parameters varied in this paper is the hydrodynamic screening "length" $\chi = \xi_s/\xi_{cell}$. This definition is unfortunate, as it differs from the usual definition adopted in the continuum active matter literature [see, e.g., Thampi et al. Phys Rev. E 90, 062307 (2014) and Refs. 20 and 29], making direct comparison more difficult. In continuum models, the screening length is typically defined as $\xi = \sqrt{\eta/\Gamma}$, where $\Gamma$ is the substrate friction and $\eta$ is the viscosity (or the intercellular friction). Instead, the present definition corresponds more closely to $1/(screeninglength^2)$. [Here, $\xi_{cell}$ carries the same dimensions as $\xi_s$ because the discretized Laplacian omits the lattice spacing factor dx, effectively absorbing a unit $dx^2 = 1$.]**

**I strongly recommend redefining the screening length as $\chi = \sqrt{\xi_{cell}/\xi_s}$. This would align the notation with the existing literature, facilitate comparison across different models, and present more natural numerical values of $\chi$ in Figs. 2 and 3.**

We have extensively revised the parameter choices to ensure consistency with existing active nematic theories. In particular, we have introduced both the hydrodynamic screening length, $l_h$, and an active length scale, $l_a$, to relate the relevant forces in the model. Furthermore, we used the non-dimensional friction number $F = l_h/l_a$ to collapse the results.

To clarify these points, we have added the following text to the Model section:

*One can non-dimensionalise the force balance equation, Eq. (2), by introducing the characteristic length and time scales, L and T respectively. This leads to the following dimensionless variables:*

$$\tilde{\boldsymbol{x}} = \frac{\boldsymbol{x}}{L}, \tilde{t} = \frac{t}{T}, \tilde{\boldsymbol{v}} = \frac{\boldsymbol{v}}{V}, \tag{4}$$

*where $V = L/T$ is the velocity scale. We can introduce these variables in Eq. (2) and reach the following non-dimensionalised equation:*

$$\tilde{\boldsymbol{v}}_i(\tilde{\boldsymbol{x}}) + \frac{l_h^2 L^2}{\Delta\tilde{x}^2} \sum_{\boldsymbol{x}' \in \mathcal{N}(\boldsymbol{x})} \sum_{j \in \boldsymbol{x}'} [\tilde{\boldsymbol{v}}_i(\tilde{\boldsymbol{x}}) - \tilde{\boldsymbol{v}}_j(\tilde{\boldsymbol{x}}')] = \boldsymbol{f}_i^p(\tilde{\boldsymbol{x}})/\xi_s V - A\boldsymbol{Q}_i \cdot \tilde{\nabla}\phi_i(\tilde{\boldsymbol{x}}), \tag{5}$$

*where $l_h = \sqrt{\xi_{cell}/\xi_s}$ and $A = \zeta T/\xi_s L^2$. From this equation we can identify one of the relevant length scales, the hydrodynamic length scale, $l_h$, which sets the typical distance over which hydrodynamic-like effects (coming from cell-cell friction) are relevant. For the passive energy term, we consider, to first order, that the main contribution for the passive restoring force comes from the $\gamma\lambda\nabla^2\phi$ term in the Cahn-Hilliard free energy, representing a penalty for distortions in the shape of the cells. We then introduce an active length scale $l_a = \sqrt{\gamma\lambda/|\zeta|}$, that denotes the characteristic length scale where active stresses overcome passive restoring forces. Combining these two length scales, we define the non-dimensional friction number, $F = l_h/l_a$ [36].*

and the following to the Discussion section:

*We introduce a non-dimensional friction number, $F$, which allows us to collapse the data and reveals the relevant scaling relations between both the characteristic strain for hole nucleation and the typical hole lifetime and the friction number. This scaling highlights the key length scales governing hole formation and dynamics, in particular the hydrodynamic and active length scales.*

**3. In Fig. 2, the fact that $P(hole|strainrate)$ follows an exponential function suggests that there is a critical strain rate required for holes to form. I think the authors can make this message more explicit, rather than just labelling this characteristic rate "the coefficient of the exponential fit" or the "slope" in the inset.**

**The authors suggest that this critical strain rate strongly depends on both friction $\chi$ and activity $\zeta$ by collapsing the fitted coefficients from different parameter sets, showing that they follow a master curve that scales with $\chi^{0.1}$. As it stands, the exponent 0.1 seems purely empirical, and it is difficult to interpret this result when $\zeta$ still has dimensions that are different from those of the strain rate ($1/[T]$). I would suggest the authors conduct a proper dimensional analysis here – e.g., either rescale the strain rate and $\zeta$ so they are dimensionless or construct a quantity from $\chi$, $\zeta$ (and most likely the surface tension $\gamma$) so that it has the same dimensions as the strain rate. Understanding the relation between the critical strain rate and the model parameters properly would be a good prediction that can be tested in experiments and in other simulation models.**

In response to this comment, we have expanded the modelling details in the main text to include a dimensional analysis of the governing equations. In particular, we highlight the ratio between the hydrodynamic screening length and the active length scale, expressed through the non-dimensional friction number $F = l_h/l_a = \sqrt{\xi_{cell}|\zeta|/\gamma\lambda\xi_s}$. In the Results section, we further introduce the parameter $\tau_F = \xi_{cell}^2/\xi_s\gamma\lambda$, which has units of time. This quantity can be constructed, by dimensional analysis, using the relevant parameters of the model ($\xi_s$, $\xi_{cell}$, $\zeta$, $\gamma\lambda$). Using these two quantities, we have replotted the inset of Fig. 2 using fully non-dimensional axes. This procedure collapses the data and emphasizes the central role of the two competing length scales in driving spontaneous hole formation. Moreover, we show that the characteristic strain rate, computed from the slopes in the main plot, scales as $\dot{\varepsilon}\tau_F \sim F^{-3}$.

To further validate this scaling, we conducted additional simulations in which we varied $\xi_{cell}$ while keeping $\zeta$ and $\xi_s$ fixed. This allows us to systematically probe the most relevant parameters governing the collapse, thereby reinforcing the observed scaling behaviour.

This part of the manuscript has been revised as follows:

*The main plot in Fig. 2 shows that $P(hole|\dot{\varepsilon})$, for different activities and substrate frictions, is well fitted by an exponential. This confirms that high strain rates are exponentially more likely to form a hole, as cells need to generate enough force to overcome the passive response. We remark that the coefficient of the exponential decreases with activity and increases with substrate friction. Higher activity or lower substrate friction generate larger strain rates, but the fraction of the large strain rates that nucleate holes decreases. We hypothesise that this is because there are competing regions of high strain rate for higher activities and lower substrate frictions, so multiple regions of the monolayer may experience large strain rates. However, since steric constraints mean that only one hole can form at a time, only one of the high strain rate regions gives rise to a hole. The inset in Fig. 2 plots the coefficient of the different exponentials, the characteristic strain rate for hole nucleation $\dot{\varepsilon}_c$ against the non-dimensional friction number $F$. Through dimensional analysis of the relevant parameters, $(\xi_s, \xi_{cell}, \zeta, \lambda\gamma)$, we introduce the time-scale $\tau_F = \xi_{cell}^2/\xi_s\gamma\lambda$, which we use to non-dimensionalise the strain rate. We observe that this is enough to collapse all the data points onto a single curve, which has slope $-3$ in a log-log scale, suggesting that $\dot{\varepsilon}_c\tau_F \sim F^{-3}$. A broader parameter range will be needed to confirm this scaling.*

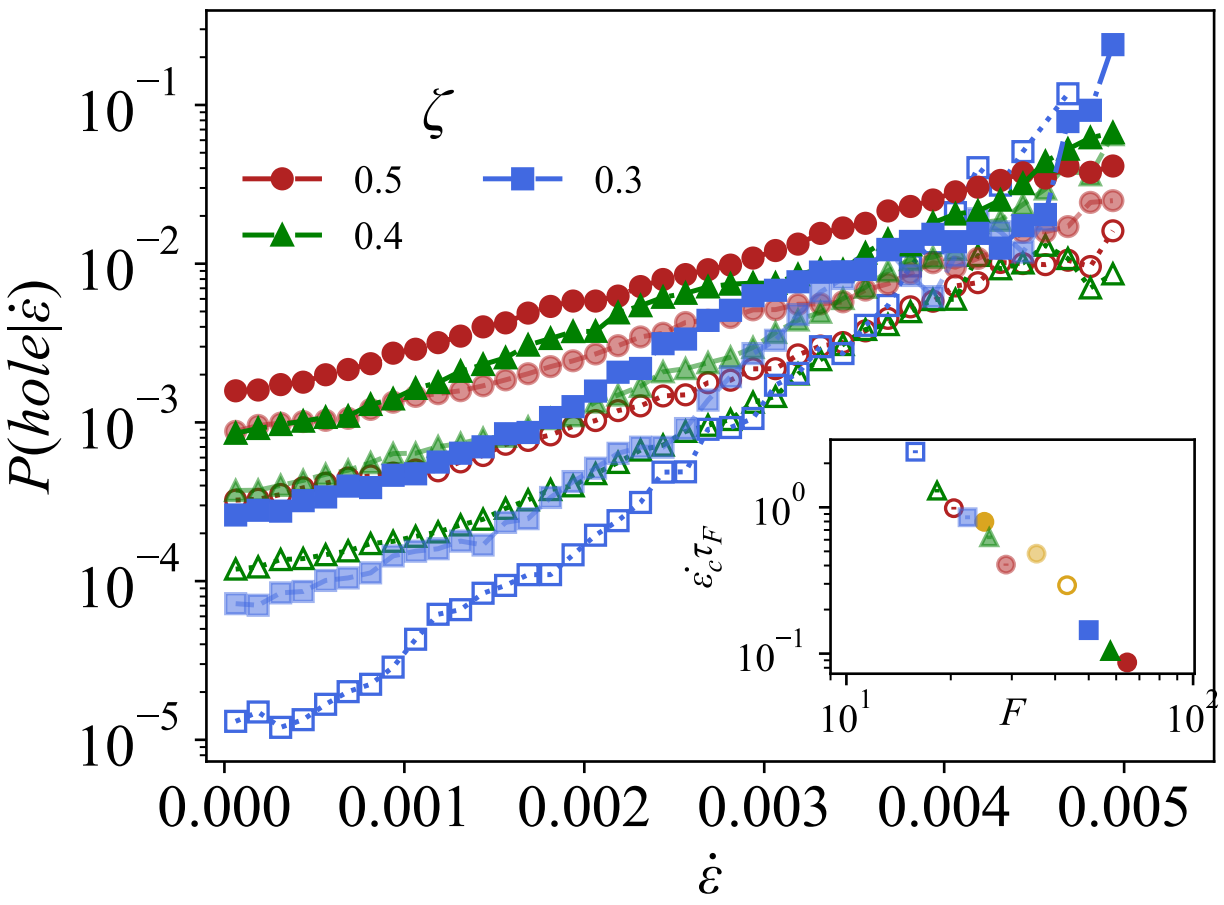


Fig. 2: **Conditional probability of forming a hole for a given strain rate** *as a function of local isotropic strain rate and activity (empty symbols correspond to* $\xi_s = 0.01$*, partially transparent symbols to* $\xi_s = 0.05$*, and full symbols to* $\xi_s = 0.001$*). The main plot shows a log-linear scale to highlight the exponential form. The strain rate field is calculated from the divergence of the velocity field. Each strain rate point corresponds to* $\dot{\varepsilon} + \Delta\dot{\varepsilon}$*, where* $\Delta\dot{\varepsilon} = 0.000125$*. The inset shows the characteristic strain rate for hole nucleation,* $\dot{\varepsilon}_c$*, non-dimensionalised by the timescale* $\tau_F = \xi_{cell}^2/\xi_s\gamma\lambda$*, as a function of* $F$*. The data points collapse onto a single line with slope* $-3$ *on a log-log scale. The golden points represent simulations for* $\zeta = 0.3$*,* $\xi_s = 0.01$*, and* $\xi_{cell} = (0.75, 0.5, 0.25)$*, for empty, partially transparent, and full symbols respectively. Each distributions is averaged over* 100 *different simulations with* 100 *different time-frames.*

**4. The paper's main outcome, whether a hole nucleates or not, is naturally controlled by two competing length scales: the hydrodynamic screening length** $l_h = \sqrt{\xi_{cell}/\xi_s}$ **(flows screened by substrate friction) and the active length scale** $l_a = \sqrt{\gamma/(|\zeta|\lambda)}$ **(activity vs elasticity; nondimensionalized by dividing by** $\lambda$**). It would be informative to test whether the 2D phase diagram in Fig. 3d collapses onto a single curve when plotted against the dimensionless ratio** $l_h/l_a$**. If hole formation is controlled by the competition between screening and activity, one expects a threshold value of** $l_h/l_a$ **separating the no-hole and hole regimes. Collapsing the data in this way would greatly clarify the physics and make the results directly comparable to existing active-matter theory. Also, in Figs. 3a and 3b, do the curves for the hole area and number of holes collapse (or obey some kind of scaling) when plotted against the ratio** $l_h/l_a$**?**

We find that by non-dimensionalising the characteristic hole lifetime, $\tau_{hole}/\tau_F$, we can collapse the results shown in Fig. 3 regarding the typical lifetime of holes, using the non-dimensional friction number $F$. The curves for hole area and number of holes do not seem to collapse or obey any kind of simple scaling with just $F$.

We have added the following to the Introduction section:

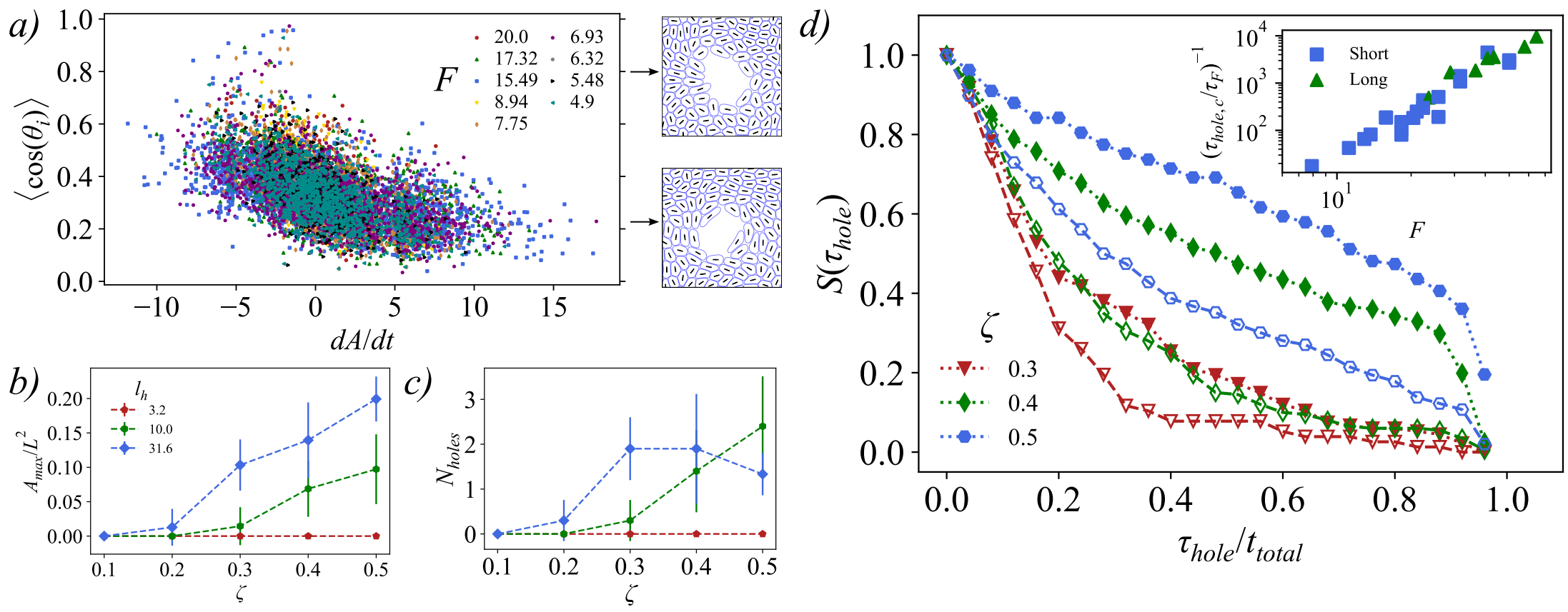

Fig. 3: **Hole lifetime statistics.** *a) Scatter plot where the y-axis reports the average cell alignment along the hole interface, quantified by* $\langle\cos(\theta_i)\rangle$*, where* $\theta_i$ *is the angle between the nematic director of cell* $i$ *and the normal to the hole interface, and the x-axis shows the change in hole area over successive time frames. Two simulation snapshots are shown on the right, one for high* $\langle\cos(\theta_i)\rangle \approx 0.8$ *(top) and another for low* $\langle\cos(\theta_i)\rangle \approx 0.2$ *(bottom). The Pearson correlation coefficient for this data set is approximately* $-0.56$*. b) Normalised maximum hole area and c) total number of holes integrated over the whole simulation as a function of activity for different substrate frictions (*$l_h = [3.2, 10.0, 31.6]$*). d) Survival probability of holes, i.e, the probability that a hole survives a fraction,* $\tau_{hole}/t_{total}$*, of the full simulation time,* $t_{total}$*, for different activities (*$\zeta = [0.3, 0.4, 0.5]$*) and substrate frictions (empty symbols correspond to* $l_h = 14.1$ *and full symbols to* $l_h = 31.6$*). We fit an exponential to each curve to calculate the characteristic hole lifetime,* $\tau_{hole,c}$*. The inset shows how the data is collapsed for* $\tau_{hole,c}/\tau_F$ *as a function of* $F$*, recovering the cubic exponent in a log-log scale. The data points are separated into short and long lived holes, corresponding to* $\tau_{hole}/t_{total} < 0.5$ *(blue squares) and* $\tau_{hole}/t_{total} > 0.5$ *(green triangles), respectively. The results in b) and c) were each averaged over* 10 *simulations with* 100 *time-frames. The results in d) were each averaged over* 100 *simulations with* 100 *time-frames.*

*We confirm that the system's behaviour is governed by two dominant length scales, the active and hydrodynamic length scales, which encode the balance of the relevant forces. Moreover, we demonstrate that a non-dimensional friction number, which links these two length scales, plays a central role: when expressed in terms of this parameter, measurements of the characteristic strain rate for hole nucleation, and of the typical hole lifetimes, collapse onto single curves.*

the following discussion to the Results:

*The survival probabilities in Fig. 3d) are well fitted by an exponential distribution, allowing us to extract the characteristic hole-lifetime timescale,* $\tau_{hole,c}$*. As in Fig. 2, we non-dimensionalise this quantity using the timescale* $\tau_F$*. The inset in Fig. 3d shows that the non-dimensional lifetime,* $\tau_{hole,c}/\tau_F$*, collapses when plotted as a function of the non-dimensional friction number,* $F$*. This collapse further underscores the importance of the two governing length scales,* $l_h$ *and* $l_a$*, in controlling both hole formation and subsequent dynamics. Once again, the collapse suggests a cubic scaling,* $\tau_{hole,c}/\tau_F \sim F^{-3}$*, reinforcing*

*the scaling already observed in Fig. 2. In experiments, holes are categorized as short or long-lived depending on their lifetime [20]. We consider the probability of forming persistent holes to be non-negligible when the lifetime exceeds a threshold $\tau_{hole} > 0.5, t_{total}$, where $t_{total}$ is the full simulation time. In physical units, this threshold corresponds to $\tau_{hole} \sim 7$ h, consistent with the onset of long-lived holes observed experimentally [20].*

and the following to the Discussion section:

*Our results propose further experiments and predictive capabilities for the extracted data. A key experiment would be to modulate tissue viscosity, for example by treatment with cytochalasin D which reduces the relative intensity of E-cadherin at cell–cell junctions [51], and then assess the resulting effects on hole nucleation. Also, we find that, to facilitate local stretching of the monolayer, two $+1/2$ defects oriented antiparallel to each other are needed to create the spiral flows that drive hole nucleation, which suggests investigating defect pairs and velocity spiral positions prior to hole formation in the experiments. We note that defects leading to spiral patterns in the cell velocity field have also been shown to modulate collective flows in bacterial colonies [52] and influence developmental and regenerative processes in Hydra [53]. More quantitatively, measuring the characteristic lifetime of holes would provide a direct test of the cubic scaling and data collapse identified here ($F^3$). In addition, measuring the correlations between cell alignment around the holes and the rate of hole growth will provide a further point of comparison between simulations and experiments.*

*It would also be of interest to compare the results presented here to other modelling approaches. For continuum models, the use of a similar non-dimensional friction number has been proposed before [36], but in the context of incompressible flows, so no holes could form. Introducing compressibility would allow for a confirmation of the scaling relations found here, and of the role of active anchoring in controlling the hole dynamics in an approach able to access longer length scales, though at a loss of cell-scale resolution.*

**5. The manuscript mostly reports whether holes form or not (e.g., Fig. 3d), but it would be valuable to also quantify how they form and evolve. In particular, for individual holes, what is the typical growth or closure law of the hole area? Including such statistics, along with representative simulation movies that show nucleation, growth, and closure (with overlays of velocity or nematic fields) would make the results much clearer and easier to connect with experiments.**

We thank the Referee for this suggestion. We agree that understanding the nucleation process is one of the key goals of this work, as it provides insight into the forces involved in hole formation. Nonetheless, we also investigated some of the statistical properties of holes. In particular, in the previous version, Fig. 3a quantifies the maximum hole area, and Fig. 3c characterizes the lifetime of holes for different parameters. Moreover, together with the data collapse discussed in the previous question, i.e., the collapse of $\tau_{hole}/\tau_F$ as a function of the non-dimensional friction number $F$, we believe that our analysis already provides insight not only into hole nucleation but also into their statistical behaviour.

To further address this point, we have added a new section focusing specifically on hole dynamics. We have included additional details describing the evolution of holes, particularly how cells align tangentially along the hole interface, a mechanism that is crucial for hole growth. We connect this behaviour to active anchoring and highlight how disrupting this anchoring influences hole size. We have also added simulation movies of hole nucleation, growth, and closure, with overlays of the velocity and nematic fields. We hope that these additions clarify our results and stimulate further theoretical and experimental studies. We have added the following section:

*Higher activity generates the forces required to overcome the elastic response of the monolayer. Notably, reduced substrate friction enhances spatial correlations, giving rise to coherent velocity fields that nucleate holes and stabilize them by promoting tangential alignment of cells along the tissue–hole interface (see simulation snapshots in Fig. 3a). This alignment is crucial: when cells at the interface are oriented tangentially, the active forces they generate act predominantly in the radial direction, opposing the passive forces that drive hole closure. For a hole to shrink, this tangential alignment must be disrupted by neighboring cells. Consequently, increasing activity or decreasing substrate friction promotes cell alignment and enhances hole stability.*
*In Fig. 3a, we quantify cell alignment at the hole interface by measuring the angle $\theta_i$ between the nematic director of cell i (extracted from Eq. 5) and the normal vector to the hole interface. This angle is averaged over all lattice sites shared between cell i and the hole, and then averaged over all cells surrounding the hole to obtain $\langle \cos(\theta_i) \rangle$. Low values of $\langle \cos(\theta_i) \rangle$ indicate cells aligned tangentially to the interface, whereas higher values correspond to alignment normal to it.*
*Figure 3a presents a scatter plot with $\langle \cos(\theta_i) \rangle$ on the y-axis and the change in hole area between successive time-frames on the x-axis. We observe a clear anti-correlation between cell elongation at the interface and hole dynamics: holes tend to grow when cells are tangentially aligned, whereas they tend to shrink when the tangential alignment is broken. The Pearson correlation coefficient for this data set is approximately $-0.56$, highlighting the inverse correlation between these two measures. This behavior is reminiscent of active anchoring [47] and provides a mechanism for both the stabilization and growth of holes.*
*Figures 3b) and c) characterise the maximum area and total number of holes as a function of activity and substrate friction, respectively. As either the activity increases or substrate friction decreases, the maximum area of holes increases as the active forces become more able to counteract the passive forces in the monolayer that promote confluency. Conversely, the total number of holes integrated over the whole simulation varies non-monotonically with activity and substrate friction. This occurs because the typical lifetime of holes increases with increasing activity and decreases with increasing substrate friction. For low activities (or high substrate friction) no holes are able to form. For higher values of activity, holes can nucleate and their lifetime increases as activity increases (or substrate friction decreases), eventually reaching a lifetime comparable to the total simulation time. Since only one hole can form at a time, long lifetimes prevent new ones from forming. This is quantified in Fig. 3d) where we plot the survival probability of the holes, i.e., the probability that a hole survives $\tau_{hole}$ units of time after nucleation. Note that, above a given value of activity, there are holes that are able to last the entire duration of the simulation.*

**6. The relatively small system size used by the authors seems to only permit**

**one or two holes to form (if I read Fig. 3b correctly). Given this, can the author provide some evidence that their results are not strongly affected by boundary effects? I understand that multiphase field simulations are computationally expensive, but I believe it is worth validating the results with a larger system size.**

We understand the concern of the Referee. We remark that the simulations were designed intentionally to only nucleate one hole at a time. This simplifies the problem as we do not have to take into account hole-hole interactions, which can be challenging to describe. Thus, we decided to pursue a moderate system size.

To prove that the results are consistent and that boundaries do not play a relevant role, we have performed additional simulations with larger system sizes ($N = 225$ and $N = 400$). We have added a new section to the Supplementary Information showing that the hole nucleation results are consistent with the $N = 100$ case. In particular, the values found for the characteristic strain rate for hole nucleation are similar. Along these lines, we have added the following text:

*F. Finite-size effects*
*To verify that boundary effects are negligible, we simulate larger systems with* $N = 225$ *and* $N = 400$. *Figure S6 shows the characteristic strain rate for hole nucleation for all three sizes on a log–linear plot, demonstrating that the exponential dependence is preserved for the larger system sizes. Moreover, the slopes of the curves, corresponding to the characteristic strain rate, are nearly identical, indicating that the boundary does not produce any noticeable effect.*
*A small vertical offset is present between the different system sizes, which we attribute to differences in the solver used for the matrix inversion required to evaluate Eq. 2. For the large matrices that must be inverted, we employ approximate conjugate-gradient methods to obtain solutions within reasonable time, as exact methods become prohibitively slow at low substrate friction values. This slowdown arises because the diagonal entries of the matrix become small compared to the off-diagonal terms. To compensate, we use a lower precision for the* $N = 400$ *case in order to simulate the same total number of time-steps in feasible computational time. For* $N = (100, 225)$ *the precision used is* $0.00001$*, whereas for* $N = 400$ *it is* $0.0001$*. We consider that the observed discrepancy in the figure is due to this difference in numerical precision.*

**Minor comments: 7. In line 110, Refs. 35-37 are cited for their relevance to the concept of the hydrodynamic screening length $\chi$. Yet, upon looking at these references, I am unable to find Refs. 35 and 37 discussing this metric. I suggest that the authors should cite other papers that explicitly mention this metric (see those in point 2 above).**

We thank the Referee for bringing up this inconsistency. We have changed the references accordingly. It now references the following works:

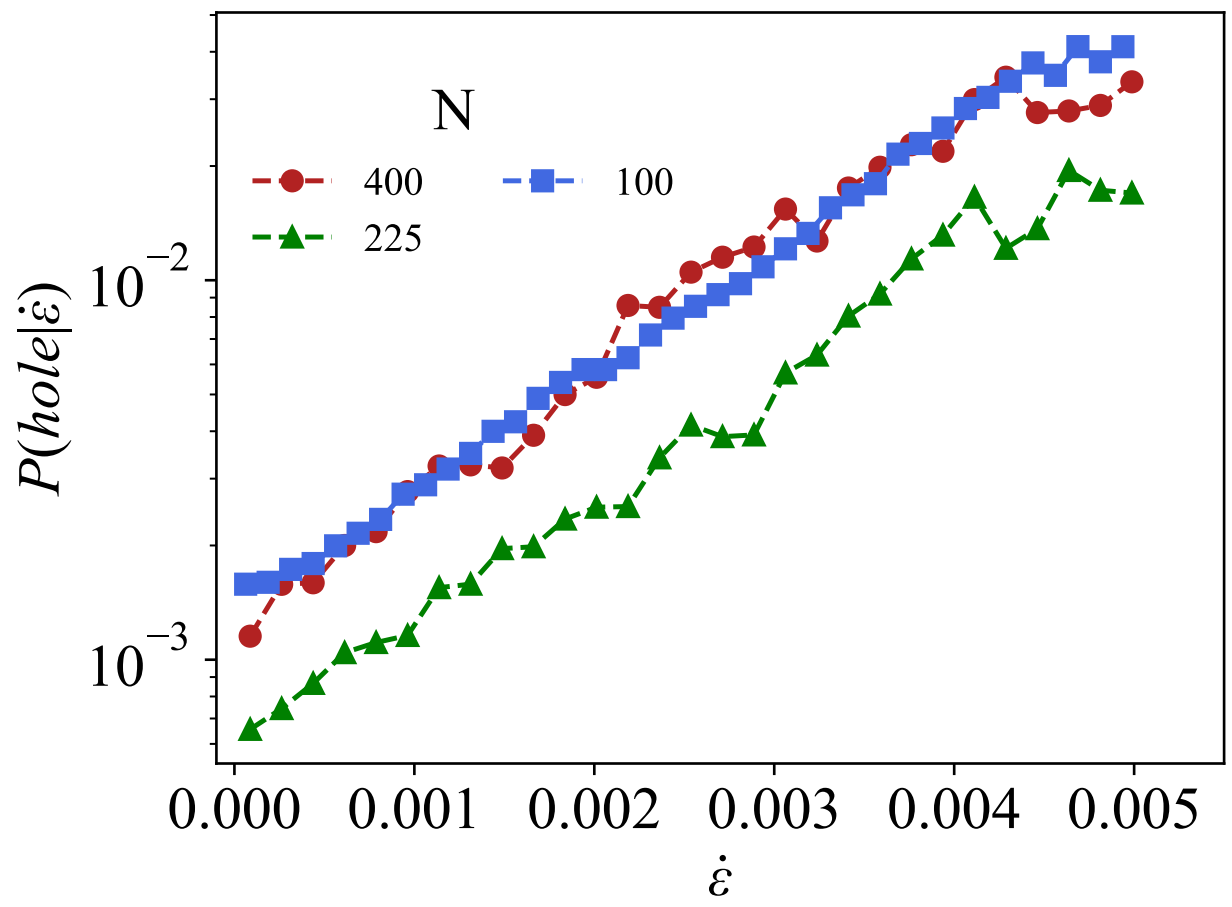


Fig. S5: **Characteristic strain rate for hole nucleation for different system sizes**. *In the plot are shown curves for different system sizes,* $N = (100, 225, 400)$*, for the same set of parameters,* $\zeta = 0.5$ *and* $l_h = 31.6$*. The results for* $N = 100$ *are the same as the ones shown in Fig. 2. For* $N = 225$ *and* $N = 400$*, the distributions are averaged over* 10 *different simulations with* 100 *different time-frames.*

[36] K. Thijssen, M. R. Nejad, and J. M. Yeomans, Role of friction in multidefect ordering, Physical Review Letters 125, 218004 (2020).

[44] S. P. Thampi, R. Golestanian, and J. M. Yeomans, Active nematic materials with substrate friction, Phys. Rev. E 90, 062307 (2014).

We have changed the text as follows:

*To focus on the effects of substrate friction and activity on the collective behaviour of cell monolayers we use the non-dimentional friction number,* $F = l_h/l_a$*, as a measure of the ratio between the hydrodynamic screening length and the active length scale [36, 44].*

**8. The correlation functions for v and Q in the Supplementary Information Section B should be labelled as Eqs. S5 and S6 to avoid reusing the same labels for the model equations given in Section A.**

We have changed the labels to the correct ones.

**The code repository still appears to be in a work-in-progress state, with no clear instructions on how to compile and run the code (the README file just says that the repository is under construction). As such, I was unable to install the program to test it.**

We thank the Referee for raising this important point. We have updated the files in the

GitHub repository to include clear instructions on installing and running the program, and we have added example cases to assist users.

# References

(Doostmohammadi, 2018) A. Doostmohammadi, J. Ignés-Mullol, J. M. Yeomans, and F. Sagués, Active nematics, Nature Communications **9**, 3246 (2018).

(Serrano, 2020) G. S. Nájera and C. J. Weijer, Cellular processes driving gastrulation in the avian embryo, Mechanisms of Development **163**, 103624 (2020).

(Petitjean, 2010) L. Petitjean, M. Reffay, E. Grasland-Mongrain, M. Poujade, B. Ladoux, A. Buguin, and P. Silberzan, Velocity Fields in a Collectively Migrating Epithelium, Biophysical Journal **98**, 1790 (2010).

(McCord, 2025) M. McCord, and J. Notbohm, Measurement of tissue viscosity to relate force and motion in collective cell migration, Soft Matter **22**, 497 (2026).

(Bonn, 2017) D. Bonn, M. M. Denn, L. Berthier, T. Divoux, and S. Manneville, Yield stress materials in soft condensed matter, Review of Modern Physics **89**, 035005 (2017).

(Chiang, 2024) M. Chiang, A. Hopkins, B. Loewe, D. Marenduzzo, and M. C. Marchetti, Multiphase field model of cells on a substrate: From three dimensional to two dimensional, Physical Review E **110**, 044403 (2024).

(Zhang, 2023) G. Zhang and J. M. Yeomans, Active forces in confluent cell monolayers, Physical Review Letters **130**, 038202 (2023).

(Mueller, 2019) R. Mueller, J. M. Yeomans, and A. Doostmohammadi, Emergence of active nematic behavior in monolayers of isotropic cells, Physical Review Letters **122**, 048004 (2019).

(Zhang, 2020) G. Zhang, R. Mueller, A. Doostmohammadi, and J. M. Yeomans, Active inter-cellular forces in collective cell motility, Journal of the Royal Society Interface **17**, 20200312 (2020).

## Response to Referee 1

**Unfortunately, I find that the resubmitted manuscript is still lacking on two major issues:**
**- relevance to experiments**
**- dimensional analysis**
**As a consequence, my opinion is that this work is not fit for publication in Nature Communications.**

We have revised the manuscript to better address and highlight these points. We hope that this updated version more clearly defines the scope of our work and conveys its key message more effectively.

**1. Relevance to experiments:**

**This work was motivated by ref. [20].**
**In [20], I read:**
**"A closer examination revealed two types of cellular event prior to hole initiation: $\sim$10 $\pm$ 3.2% of holes were associated with breakage of junctions following stretching of a cell (Extended Data Fig. 1c and Supplementary Video 4), and $\sim$90 $\pm$ 4.1% were formed at cell-division sites during abscission"**

**Since the model does not include cell division among its ingredients, its predictions pertain to only one in ten hole formation events observed experimentally in [20].**

**I read, l.325-326:**
**"The cell monolayer experiments suggest that cell divisions also play a relevant role in hole formation [20]."**
**Please correct this statement.**
**According to the experiment, cell division plays the dominant role in hole formation.**

**In addition, the experimental average velocity field close to hole formation events is given in Extended Fig. 4b of Ref. [20]. The pattern differs markedly from the spiral shown in Fig. 1b of this work.**

**Finally, there is no mention in Ref. [20] of defect pairing prior to hole formation.**

**I conclude that the Authors' predictions disagree with current experimental observations.**
**This fact should be stated explicitly in the manuscript.**

Regarding Ref. [20], we note that in the same paragraph cited by the Referee, the authors

demonstrate, through mitomycin-C experiments, that hole formation persists even when cell division is fully inhibited. Figure 1 below, extracted from that reference, illustrates this clearly: the left column shows hole formation with active cell division, while the right column shows only a minor change when division is suppressed. This provides direct experimental evidence that cell stretching acts as an independent mechanism.

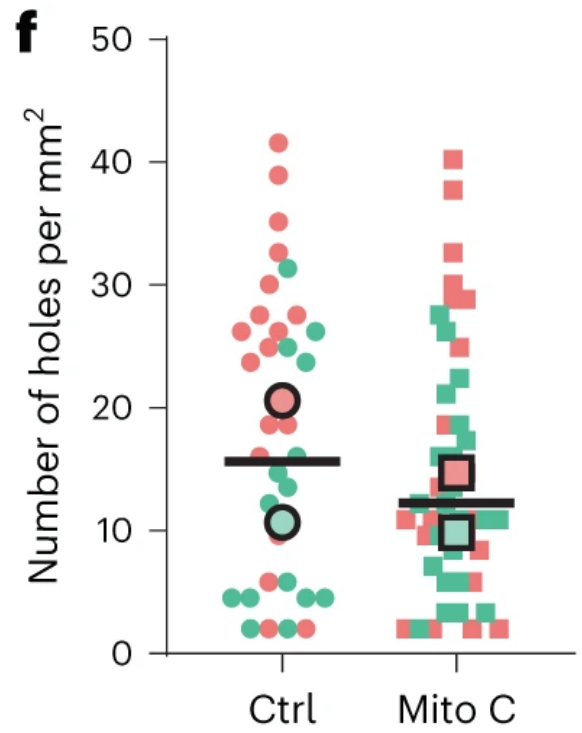


Fig. 1: The total number of holes formed within untreated and mitomycin-C-treated circles on soft (2.3 kPa) gels ($n_{ctr} = 33$, $n_{mito} = 44$). All results were obtained from two independent experiments, with encircled coloured symbols for the mean per experiment and solid bars for the mean over experiments. (adapted from Fig. 1f in Ref. [20])

The defect pairing we predict is indeed not discussed in Ref. [20]. Our goal is not to challenge the experimental findings, but rather to suggest an additional mechanism that may not have been previously considered, namely, the role of defect pairing, as a possible mechanism for hole formation. Recent studies in other active systems have also emphasized the role of multi-defect interactions in hole formation (Chen, 2026), and we propose a similar mechanism may be relevant for cell monolayers. Our intention is therefore to complement, rather than revise, the existing literature.

Regarding the velocity fields in Extended Fig. 4b of Ref. [20] mentioned by the Referee, those correspond to averaged velocity fields in the vicinity of a $+1/2$ defect. In contrast, Fig. 1b of our paper shows the velocity field associated with hole nucleation. Figure 2 below compares the average velocity fields centred on these defects, obtained from experiments (a) and simulations (b). The comparison highlights that the averaged flow field around $+1/2$ defects is consistent between experiments and our simulations, while remaining clearly distinct from the velocity field observed during hole formation (c). This velocity profile around $+1/2$ defects is also in good agreement with what is predicted from active nematic theories (Doostmohammadi, 2018).

We have added the following text to the Discussion section:

*In contrast to the experiments we do not see hole formation at $-1/2$ defects in the simulations. This may be due to not taking anisotropic effects into account. Hole formation at $-1/2$ defects in bacterial colonies [54] has been connected to anisotropic friction effects and introducing this into the multi-phase field model will be an interesting topic for future*

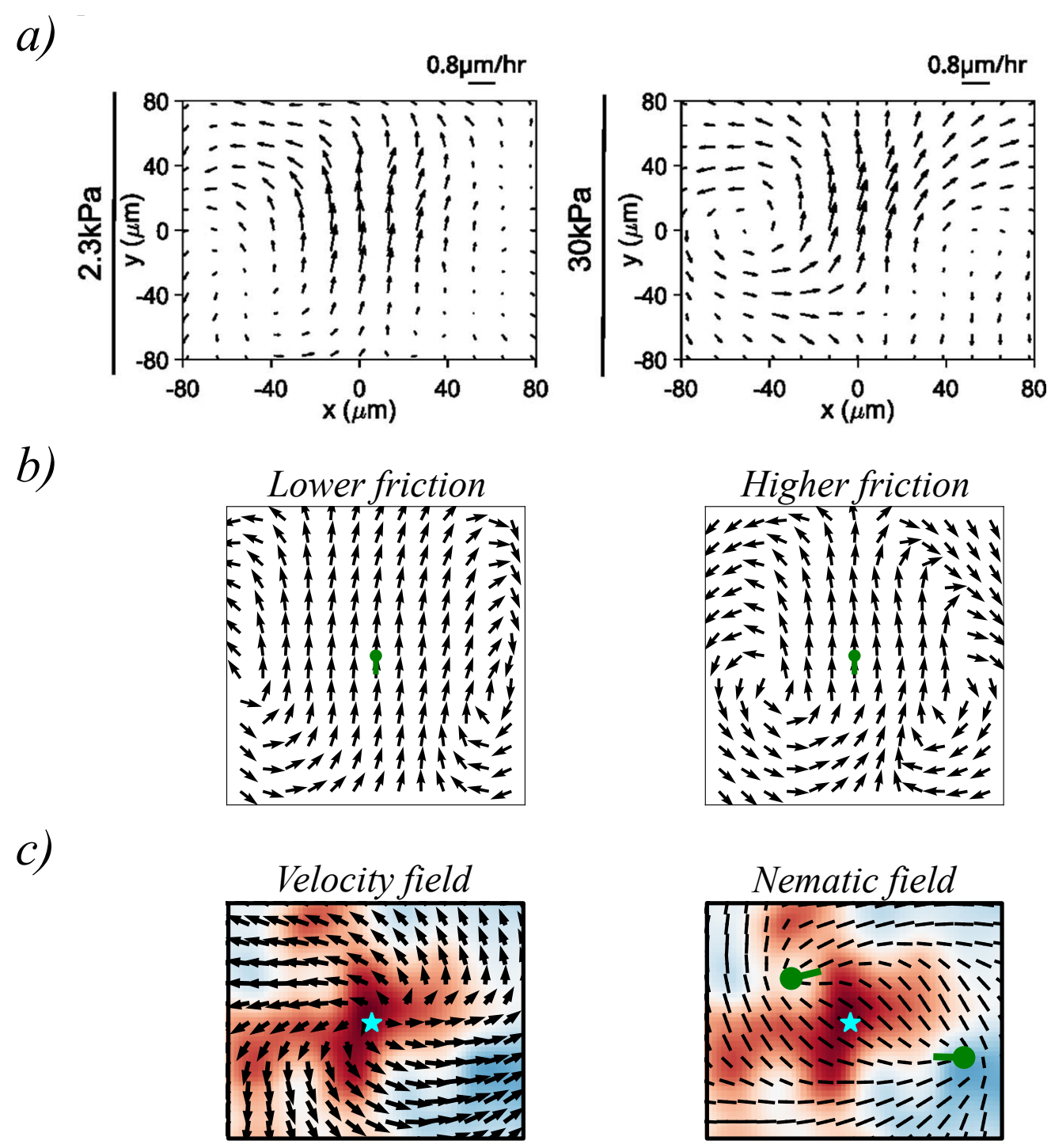


Fig. 2: a) Average velocity flow field around +1/2 defects demonstrating the extensile nature of MDCK monolayers on soft (2.3 kPa) gels ($n = 2646$ defects) and stiff (30 kPa) gels ($n = 2765$ defects) obtained from experiments (adapted from Extended Data Fig. 4b in Ref. [20]). b) Average velocity flow field around +1/2 defects ($n = 1000$ defects) obtained from simulations. Each box is $60 \times 60$ lattice points. The "Lower friction" frame corresponds to $l_h = 31.6$, while the "Higher friction" frame to $l_h = 3.2$. Both are for $\zeta = 0.3$. c) Velocity and nematic fields close to a hole nucleation site as represented in Fig. 1b of the main text.

*research. Although cell division may also contribute to anisotropic effects, experiments on mitomycin-C treated monolayers [20] indicate that division is not a necessary condition for hole formation. The persistence of this behavior in the absence of cell divisions suggests that cell stretching constitutes a primary driving mechanism.*

**2. Elementary dimensional analysis:**

**It remains unclear throughout the manuscript whether parameters and fields are dimensionful or dimensionless.**

**- According to Eq. (2), $\xi_{cell}$ and $\xi_s$ do not have the same unit. Indeed their ratio allows to define the square of the hydrodynamic length.**
**I read p. 13, l. 288:**
**"In the friction dominated regime $\xi_{cell} \leq \xi_s$..." Since units differ, this inequality does not make sense.**

**- Since $\xi_{cell}$ and $\xi_s$ do not have the same unit, I would use different symbols**

**for each. Since $\xi_cell$ is, in fact, a viscosity, not a friction coefficient, why not simply call it $\eta$ instead of $\xi_{cell}$? This is the standard practice in the field.**

**- In Eq. (7), the factor $l_h^2 L^2/\Delta\bar{x}^2$ cannot be dimensionless.**

**- l. 139 and 143, inequalities such as $l_h \gg 1$ do not make sense, since $l_h$ (without a $\bar{}$) is a length.**

**- The reference timescale T has not been defined.**
**For consistency, I would suggest to use $\tau_F$ as a reference timescale.**

**I strongly recommend that the Authors proofread their manuscript and check that their results are everywhere consistent with dimensional analysis. For clarity, dimensionless quantities should always carry a $\bar{}$.**

We thank the Referee for bringing these typos to our attention. We have corrected the typo in Eq. (10) to properly provide the non-dimensional quantity. We have also reviewed the inequalities along the text to make sure they provide the correct non-dimensional information. We now identify dimensionless variables with an overbar.

Regarding the symbol for $\xi_{cell}$, we do agree that this is a viscosity-like parameter, but as there is no explicit background fluid flow in the multi-phase field modelling approach we use, this is arguably an overstatement. In fact, many recent works have been careful to call it a cell-cell friction term (Rozman, 2025; Chiang, 2024; Nguyen, 2026), to better highlight the underlying mechanics.

In the Model Section we have added the following:

*We adopt the characteristic length scale $L_0 = \lambda$, which sets the interface width where the relevant dynamics occur, and, based on dimensional analysis of $(\xi_s, \xi_{cell}, \zeta, \lambda\gamma)$, the characteristic time scale $T_0 = \xi_{cell}^2/(\xi_s\gamma\lambda) \equiv \tau_F$.*

**1. The new title emphasizes the role plaid by internal dissipation in hole formation in this model. This point should also be mentionned in the abstract. Since this is the main result of this work, this result should be illustrated explicitly, and perhaps Section B of the supplementary Material should be moved to the main text. I would also suggest adding a figure similar to Figs. S3b and S4b, where the hole density would be plotted as a function of the viscosity.**

We have changed the abstract to highlight the effects of internal dissipation. As the relevant limits are already thoroughly described in the first paragraph of the Results section, we have chosen to keep the figures in the Supplementary Material, thus keeping the Results section of the main manuscript focused on hole formation.

We have rewritten the abstract in the following way:

*Although cell monolayers typically remain confluent, they can spontaneously develop persistent holes as a result of collective cellular motion. Recent studies on MDCK monolayers cultured on soft substrates have revealed that cells can align to create regions of local nematic order, and topological defects that generate localised mechanical stresses which can spontaneously trigger hole formation. To investigate this process, we develop a continuum multi-phase field model that incorporates internal dissipation and active dipolar forces that drive cell shape anisotropy. Our simulations show that reducing substrate friction enhances cell-cell velocity correlations. In the low-friction regime, topological defects generate spiral flow patterns that concentrate stress and can trigger hole formation. By contrast, in the high-friction regime, holes do not nucleate. We further demonstrate that the number and stability of the holes—whether they close or persist—depends on both substrate friction and cellular activity, through a non-dimensional friction number. These findings highlight the importance of internal dissipation in modelling collective cell motion and the critical role of collective dynamics in maintaining tissue integrity.*

**2. I would move Sec. A from SI to main text. In the present manuscript, important parameters such as $\gamma$ (l. 110), or the surface tension (l.175) appear in the text without having been defined first.**

We have integrated parts of the Supplementary Material information into the main text to make the relevant parameters clearer:

*The free energy term defines the dynamics of the individual interfaces and is written as* $\mathscr{F} = \mathscr{F}_{CH} + \mathscr{F}_{area} + \mathscr{F}_{rep}$, *where*

$$\mathscr{F}_{CH} = \sum_i \frac{\gamma}{\lambda} \int d\boldsymbol{x} \big[4\phi_i^2(\boldsymbol{x})(1-\phi_i(\boldsymbol{x}))^2 + \lambda^2(\nabla\phi_i(\boldsymbol{x}))^2\big], \tag{1}$$

$$\mathscr{F}_{area} = \sum_i \mu \left[1 - \frac{1}{\pi R^2} \int d\boldsymbol{x} \phi_i^2(\boldsymbol{x})\right], \tag{2}$$

$$\mathscr{F}_{rep} = \sum_i \sum_{j\neq i} \frac{\kappa}{\lambda} \int d\boldsymbol{x} \phi_i^2(\boldsymbol{x})\phi_j^2(\boldsymbol{x}). \tag{3}$$

*Equation* (1) *is a Cahn-Hilliard free energy that encourages* $\phi_i(\boldsymbol{x})$ *to be equal to* 1 *or* 0 *inside or outside cell* $i$, *respectively. The cell boundary is located at* $\phi_i(\boldsymbol{x}) = 1/2$ *and has width* $\sim 2\lambda$, *set by the gradient term.* $\gamma\lambda$ *sets an energy scale. This term controls the deformability of the individual cells. Cell compressibility is described by Eq. (2), which imposes a soft constraint, of strength* $\mu$, *restricting the area of each cell to* $\pi R^2$. *Equation (3) penalizes overlap between cells with an energy scale* $\kappa\lambda$.

**3. Wherever possible, "strain rate" should be replaced by "isotropic strain rate" for clarity. One naturally wonders whether large values of the shear strain rate also lead to hole formation in this model.**

We thank the referee for this suggestion. We agree that "isotropic strain rate" is the more precise terminology and have adopted it throughout the manuscript for clarity.

Regarding the role of shear, we have now explicitly quantified the shear (deviatoric) strain rate and included the results in the Supplementary Material (Fig. S6 and Video S7). We find that the shear strain rate also exhibits a peak near the time and location of hole nucleation, indicating that this event coincides with a localization of total deformation in the tissue. However, its statistical behaviour differs qualitatively from that of the isotropic component: while the isotropic strain rate shows a clear exponential dependence, the shear strain rate displays a broader, and potentially bimodal, distribution, reflecting the heterogeneous nature of local cell deformations.

These observations suggest that the two quantities play distinct but complementary roles. The isotropic strain rate captures the local dilation required to overcome the forces maintaining confluency and is therefore directly linked to hole nucleation. At the same time, the shear strain rate quantifies the local distortions of the tissue, which contribute both to the formation of the spiral flow patterns observed in the velocity field and to the subsequent growth of the hole, where significant deformation of cells along the interface is required.

To highlight this new analysis, we have added a new section to the Supplementary Material:

*G. Shear strain rate*

*In the main text, we focus on the role of the isotropic strain rate in hole formation. Here, we perform an analogous analysis for the shear strain rate, defined as*

$$\dot{\varepsilon}_s = \sqrt{(\partial_x v_x - \partial_y v_y)^2 + (\partial_x v_y + \partial_y v_x)^2}. \tag{4}$$

*Figure S6a shows that the maximum shear strain rate also peaks near the location and time of hole nucleation (see Supplementary Video S5), indicating that this event coincides with a localization of the total deformation within the tissue. Thus, as expected, the nucleation site corresponds to the region where the tissue undergoes the strongest overall distortion. In Fig. S6b we present the statistics of the shear strain rate at the onset of hole formation. In contrast to the isotropic component, which follows an exponential distribution, the shear strain rate exhibits a more complex, possibly bimodal, distribution. This behaviour likely reflects the heterogeneous nature of cell deformations in the vicinity of the nucleation site, where regions of strong distortion coexist with less deformed areas.*
*Taken together, these observations suggest that the isotropic and shear strain rates play distinct but complementary roles in hole creation. The isotropic strain rate captures the local dilation required to overcome the forces maintaining confluency and is therefore directly linked to hole nucleation. In contrast, the shear strain rate quantifies local distortions of the tissue, which contribute to the formation of the spiral flow patterns observed in the velocity field and play an important role in the subsequent growth of the hole, where significant deformation of cells along the interface is required.*

We have also added the following to the main text:

*In Supplementary Material Sec. G, we show that the deviatoric component of the strain*

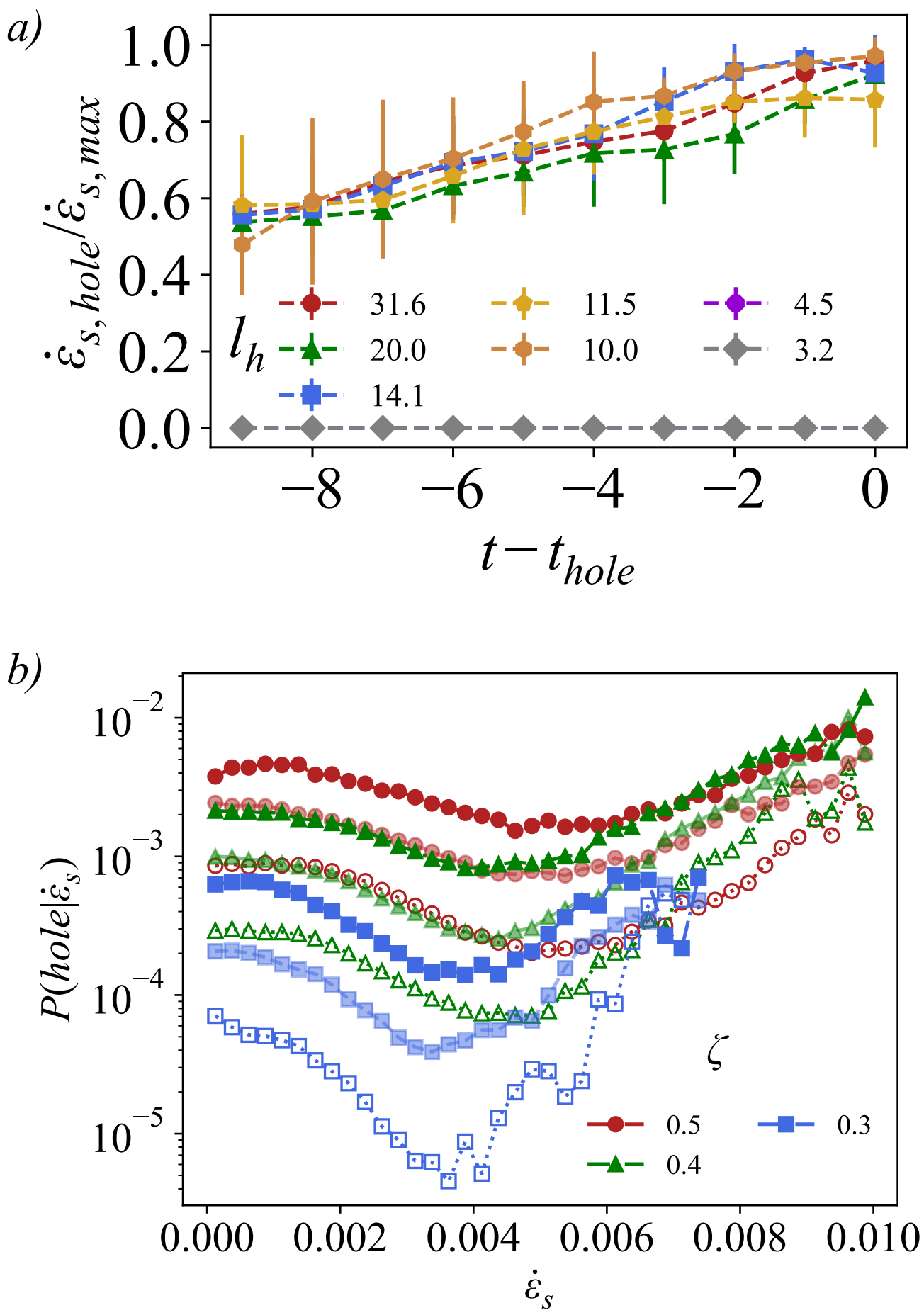


Fig. S6: **Statistics of the shear strain rate during hole formation**. *a) Normalised maximum shear strain rate in an area of radius* $R/2$ *around the location of hole nucleation as a function of the number of time-frames before nucleation. The normalization is per time-frame, where each time-frame spans* 1000 *time-steps. These results were averaged over* 10 *simulations with* 100 *time-frames, and with constant* $\xi_{cell} = 1$. *b) Conditional probability of forming a hole for a given shear strain rate as a function of local shear strain rate and activity (empty symbols correspond to* $\xi_s = 0.01$, *partially transparent symbols to* $\xi_s = 0.05$, *and full symbols to* $\xi_s = 0.001$*). Each shear strain rate point corresponds to* $\dot{\varepsilon} + \Delta\dot{\varepsilon}$, *where* $\Delta\dot{\varepsilon} = 0.000125$. *Each distributions is averaged over* 100 *different simulations with* 100 *different time-frames.*

*rate tensor also peaks near hole nucleation (see Supplementary Video S5), indicating strong local distortions of the tissue that contribute to the formation of the spiral flow patterns observed in the velocity field.*

**4. It would be interesting to plot the strain and (isotropic) strain rate fields close to hole formation events. Since the model comes with a well-defined reference area (Eq. S2), the strain can also be evaluated without ambiguity.**

We thank the referee for the suggestion. However, we respectfully disagree that the model provides an unambiguous definition of strain. The area term sets a preferred cell area, but it does not define a material reference configuration for the tissue. In a confluent, actively rearranging monolayer, any finite strain field necessarily depends on an arbitrary choice of reference time and on the treatment of cell-scale remodeling (neighbor exchanges, shape changes, etc.), and is therefore not uniquely defined. By contrast, the isotropic strain rate is a local, instantaneous quantity directly obtained from the velocity field and does not suffer from these ambiguities. For this reason, we focus on the strain rate, which is the physically well-defined and relevant observable for the dynamics leading to hole formation.

**5. l. 281: strain → strain rate (unless a characteristic strain can be defined)**

We have corrected this typo.

**6. Since the function S is fitted by an exponential, Fig 4d should be plotted in lin-log scale, instead of lin-lin scale.**

We have replaced Fig. 4d with the log-lin scale, to highlight the exponential dependency (see Fig. 3 below).

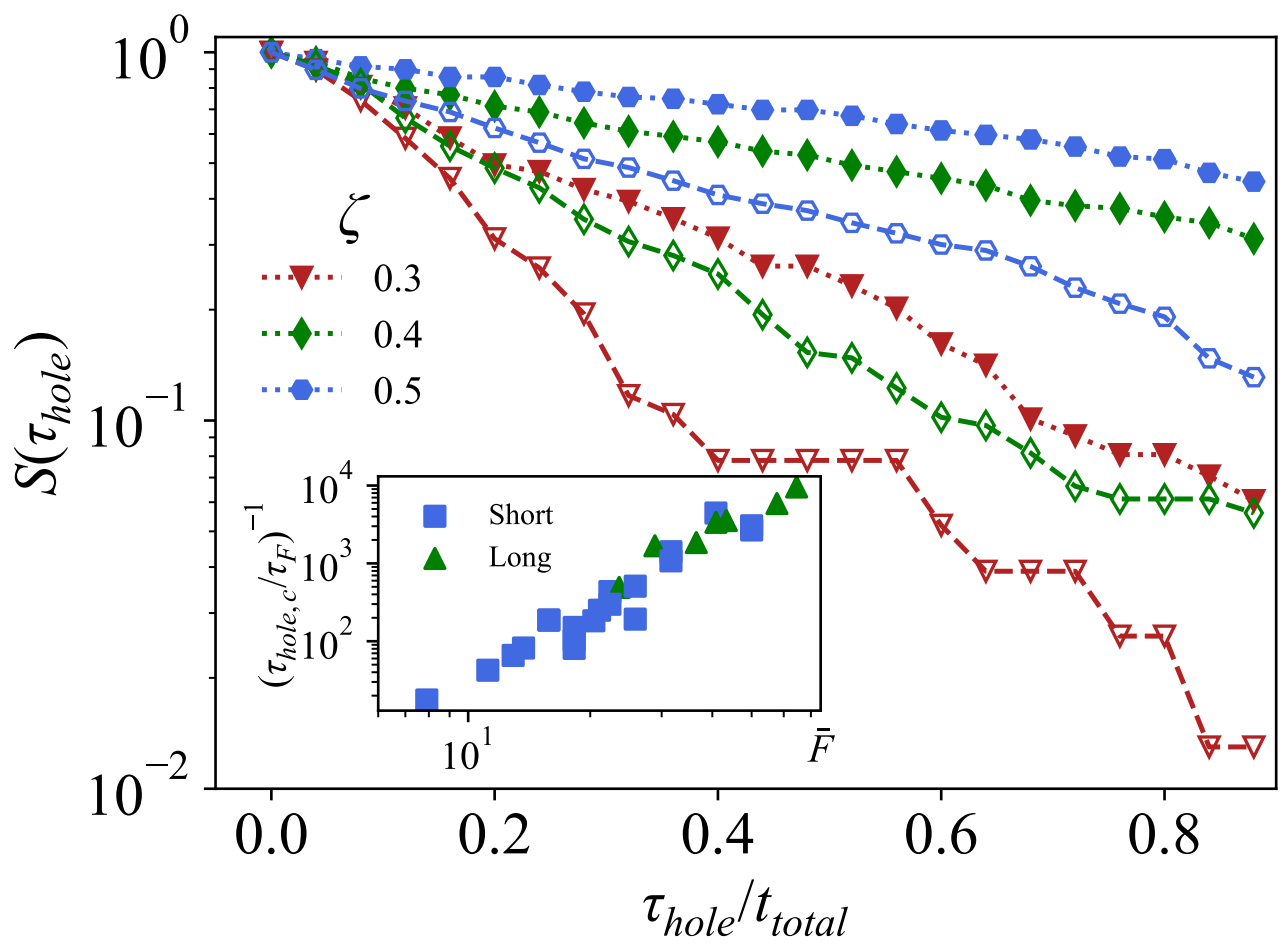


Fig. 3: Survival probability of holes, i.e, the probability that a hole survives a fraction, $\tau_{hole}/t_{total}$, of the full simulation time, $t_{total}$, for different activities ($\zeta = [0.3, 0.4, 0.5]$) and substrate frictions (empty symbols correspond to $l_h = 14.1$ and full symbols to $l_h = 31.6$), on a log-lin scale. We fit an exponential to each curve to calculate the characteristic hole lifetime, $\tau_{hole,c}$. The inset shows how the data is collapsed for $\tau_{hole,c}/\tau_F$ as a function of $F$, recovering the cubic exponent in a log-log scale. The data points are separated into short and long lived holes, corresponding to $\tau_{hole}/t_{total} < 0.5$ (blue squares) and $\tau_{hole}/t_{total} > 0.5$ (green triangles), respectively.

**Minor comments:**

**- in Eq, (5) the integration variable is d $\vec{x}$ not dx**

**- l. 297: epithiloid → epithelioid**

We have corrected these typos.

## Response to Referee 2

**The authors have done substantial revisions to the manuscript, and I appreciate that they have taken the time to address the concerns I had raised. Overall, I find the revised manuscript much strengthened scientifically: the new presentation of the non-dimensional friction parameter $F = l_h/l_a$, as suggested in the first round of review, captures well the physics of hole formation in this system. It yields new scaling relations and exponents (e.g., the characteristic strain rate scales with $F^{-3}$) that can be tested in other active soft matter systems. The new result that cell alignment along the hole interface depends on hole growth or shrinkage provides additional tangible predictions that can be validated in experiments.**

We thank the Referee for the positive review and for the suggestions that helped us to improve our work.

**I still have a few minor comments that I would like the authors to address before recommending publication:**

**1. Regarding my original concern about the passive force term (major point 1), I would recommend the authors stating clearly in the manuscript (either in the main text or in the SI) the reason for choosing to simulate this force term as $f^p = -\nabla(\delta F/\delta\phi)$, as it differs from what they have done previously and from variational derivation.**

We agree with the Referee's suggestion and have added the following to the manuscript:

*This formulation of the passive forces differs from previous approaches [24, 40], which can produce non-zero net forces and induce unidirectional flows under periodic boundary conditions, particularly at low substrate friction. In contrast, our formulation derives passive forces from a gradient term, ensuring a zero net force in periodic domains. Since the role of these forces is to drive the system toward free-energy minima while remaining globally force-balanced, we do not expect these differences to qualitatively affect the results.*

**2. In the non-dimensionalized force balance equation (Eq. 7), I believe there is a typo: $L^2$ should be in the denominator in the intercellular friction term.**

We have corrected this typo.

**3. The authors should state the p-value for the Pearson correlation between the cell alignment along the hole interface and the rate of change of the hole area, as shown in Fig. 3a.**

We thank the referee for this suggestion. The p-value is approximately 0 as the sample size is of the order of $10^4$. We have added the following to the text:

*The Pearson correlation coefficient for this dataset is* $-0.56$*, with a p-value* $P < 0.001$ *(*$n_{samples} = 10^4$*,* $t(9998) = 67.59$*), indicating a statistically significant inverse relationship between the two variables.*

**4. Please also state the parameter values used for the simulation shown in the supplemental videos. In Video S4, which shows the active force density field, it seems that horizontal and vertical bands appear throughout the video. Can the authors confirm that these are not artifacts due to simulating the phase fields in subdomains?**

We have added the relevant parameters to the video captions. Regarding the active force density field, the active force is highly anisotropic (being strongest at the cell interface). As a result, applying a smoothing window (of approximately one cell diameter) introduces some visual distortions as observed in the video. To clarify this point, we have included a new supplementary video (Supplementary Video S6) that directly visualizes the active forces without smoothing, thereby highlighting their anisotropic nature.

**5. There are some typos in the SI: - In the caption of Fig. S3, the old parameter $\chi$ is still reported; the author should convert that to the new parameter $l_h$. - In Section F, Figure S6 should be Figure S5.**

We have corrected these typos.

**While I was able to compile the code following the instructions in the README, I was unable to run the program, getting errors saying that the configuration file cannot be read. The authors should provide more instructions on how the configuration and parameter files should be specified, as well as a minimal working example.**

For the configuration file, one needs to run the *confGenerator* executable first to generate the initial conditions. We have revised the README file and added a point-by-point explanation on how to run a specific example simulation for our code package (Examples section).

**References**:

(Chen, 2026) S. Chen, M. Ricci, A. P. Tabatabai, Z. G. Sun, S. Witthaus, S. Shankar, M. Nitzan, and M. P. Murrell, Topological control of spontaneous failure in active nematic solids, Nature Materials **25**, 659 (2026).

(Doostmohammadi, 2018) A. Doostmohammadi, J. Ignés-Mullol, J. M. Yeomans, and F. Sagués, Active nematics, Nature Communications **9**, 3246 (2018).

(Rozman, 2025) J. Rozman, K. V. S. Chaithanya, J. M. Yeomans, and R. Sknepnek, Vertex model with internal dissipation enables sustained flow, Nature Communications **16**, 530 (2025).

(Chiang, 2024) M. Chiang, A. Hopkins, B. Loewe, M. C. Marchetti, and D. Marenduzzo, Intercellular friction and motility drive orientational order in cell monolayers, Proceedings of the National Academy of Sciences **121**, e2319310121 (2024).

(Nguyen, 2026) A. Q. Nguyen, P. K. Bera, J. Notbohm, and D. Bi, Cell-Cell Adhesion as a Double-Edged Sword in Tissue Fluidity, arXiv preprint arXiv:2603.04170 (2026).
