# Supplementary material for: Multi-phase field model reveals internal dissipation is crucial for spontaneous hole formation in cell monolayers
Source: Nat Commun. 2026 Jul 3;17:8288. doi: 10.1038/s41467-026-74977-y (PMC13469578; doi:10.1038/s41467-026-74977-y)
Supplement: Supplementary file 3 [file 41467_2026_74977_MOESM8_ESM.pdf]

Supplementary Information: Multi-phase field model reveals internal dissipation is crucial for spontaneous hole formation in cell monolayers

Diogo E. P. Pinto
The Rudolf Peierls Centre for Theoretical Physics,
Clarendon Laboratory, Parks Road, Oxford, OX1 3PU, UK
Centro de Física Teórica e Computacional, Faculdade de Ciências,
Universidade de Lisboa, P-1749-016 Lisboa, Portugal and
Departamento de Física, Faculdade de Ciências, Universidade de Lisboa, P-1749-016 Lisboa, Portugal

Jan Rozman
The Rudolf Peierls Centre for Theoretical Physics,
Clarendon Laboratory, Parks Road, Oxford, OX1 3PU, UK
Jožef Stefan Institute, Jamova 39, SI-1000 Ljubljana, Slovenia and
University of Ljubljana, Faculty of Mathematics and Physics, Jadranska 19, SI-1000 Ljubljana, Slovenia

Julia M. Yeomans*
The Rudolf Peierls Centre for Theoretical Physics,
Clarendon Laboratory, Parks Road, Oxford, OX1 3PU, UK

A. Simulation details

We simulate Eqs. (1)-(8) using a finite-difference scheme on a square lattice with a predictor-corrector step. We also use a space decomposition method, as in [1], where each phase field is only solved inside a subdomain that covers an area of 30×30 lattice sites, centred on the centre of mass of the phase field. This subdomain is variable after the equilibration protocol. We set a threshold value of $\phi_i(\mathbf{x}) = 0.01$, and if the distance from $\mathbf{x}$ to the sides of the subdomain is smaller than 4 lattice sites, then we increase the subdomain size. On the other hand, if it is larger, we decrease it. We also do not enforce a square subdomain. This optimization helps deal with highly elongated cells without fixing a large subdomain from the start.

We simulate the dynamics of cells of radius $R = 8$ in a periodic domain. Initially, cells with radius $R/2$ are placed randomly. They are then relaxed for 10000 time-steps under passive dynamics without cell-cell friction to reach confluence. The simulations are then run for 100000 time-steps and the state of the simulation is printed every 1000 time-steps. The lattice spacing is set to $\Delta x = 1$ and the interval between time-steps is set to $\Delta t = 0.1$. Similar to previous work [2] and to keep the physical values within acceptable ranges (see Table S1), we chose the following parameter values: $\lambda = 2.0$, $\gamma = 0.06$, $\kappa = 0.5$, $\mu = 20$, and $J_0 = 1$.

Based on typical values for MDCK cells, such as an average radius of approximately 10μm, velocity around 20μm/h, and pressure on the order of 100 Pa, as measured via Particle Image Velocimetry and Traction Force Microscopy [3], we estimate that the simulation units correspond to physical units of $\Delta x \sim 1$ μm, $\Delta t \sim 5$ s, and $\Delta F \sim 1.5$ nN for length, time, and force, respectively [2]. The rest of the parameters are summarized in Table S1.

* julia.yeomans@physics.ox.ac.uk

Parameter	Interpretation	Dimensions	Value(s) in simulation units	Value(s) in physical units
Δx	Size of each lattice site	$[L]$	1	1 μm
Δt	Size of each timestep	$[T]$	1	5 s
ΔF	Force scale	$[F]$	1	1.5 nN
γ	Surface tension	$[F]$	0.06	0.09 nN
λ	Interfacial thickness	$[L]$	2	2 μm
μ	Area constraint	$[F][L]$	20	30 nN$\cdot\mu$m
κ	Cell–cell repulsion	$[F]$	0.5	0.75 nN
R	Ideal cell radius	$[L]$	8	8 μm
ξ_s	Cell–substrate friction	$[F][T][L]^{-3}$	0.001 - 1.0	0.0075 - 7.5 kPa$\cdot$s/μm
ξ_{cell}	Cell–cell friction	$[F][T][L]^{-1}$	0.1 - 1.0	0.75 - 7.5 MPa$\cdot$s$\cdot\mu$m
ζ	Active stress	$[F][L]^{-1}$	0.1 - 0.5	0.15 - 0.75 nN/μm

Supplementary Table 1. A full list of the model parameters, their dimensions, and their values both in simulation and physical units.

The monolayers' coarse-grained fields can be calculated at each lattice point using

$$A(\mathbf{x}) = \sum_i a_i(\mathbf{x})\phi_i(\mathbf{x}), \tag{1}$$

where $a_i(\mathbf{x})$ corresponds to a given measure at the lattice site $\mathbf{x}$, such as velocity, active force or nematic field. Since the nematic director is given by the elongation of the cell, we consider that $Q_i(x) \equiv Q_i$. We then smooth each individual field to reduce noise using a sliding window of size $2R \times 2R$. A defect is identified at a point $\mathbf{x}$ of a smoothed field by examining the value of the field at the 8 neighbouring points of $\mathbf{x}$. The orientation of the defects is calculated by the method in Ref. [4]. We calculate the isotropic strain rate using the smoothed velocity field, $\dot{\varepsilon}(\mathbf{x}) = \nabla \cdot \mathbf{v}(\mathbf{x})/2$. We consider a hole has nucleated when the void area is above $\pi R^2/4$, and we set a lattice site to be void when the sum of all phase fields on it is below a threshold, i.e., $\sum_i \phi_i(\mathbf{x}) < 0.01$.

B. Velocity and nematic director correlations with internal dissipation

Previous modelling approaches with the multi-phase field model have mostly focused on using substrate friction as the main dissipation contribution [2, 5]. Recently, cell-cell friction has been introduced into this framework, in the context of polar active cells [6, 7]. The main difference to our implementation is that here we calculate the velocity field at every lattice point, instead of only at the centroid of the cell. This makes the description proposed in Eq. (5) of the main text closer to a Laplacian in the local velocity field, similar to continuum theories [8].

Here, we highlight the main differences between a *dry* modelling approach ($\xi_{cell} = 0$) and a *wet* modelling approach ($\xi_{cell} > 0$ and $\xi_s \to 0$), as commonly referred to in the literature (see Ref. [9]). Figure 1 shows that in the *dry* model correlations in the velocity and nematic director decay quickly, with a correlation length of the order of the single cell radius ($r < 8$). As we approach the *wet* model regime, these correlations increase as the substrate friction ξ_s is decreased. Similarly to active nematic theories, substrate friction acts as a screening for hydrodynamic effects [10].

The correlations are calculated through the non-smoothed velocity and nematic fields. We create the respective fields at every lattice point using Eq. (1) and then calculate the correlations for a given snapshot after 100000 time-steps,

$$C_{\mathbf{v}}(r) = \frac{\langle \mathbf{v}(\mathbf{x}) \cdot \mathbf{v}(\mathbf{x}') \rangle}{\langle \mathbf{v}(\mathbf{x}) \cdot \mathbf{v}(\mathbf{x}) \rangle}, \tag{5}$$

$$C_{\mathbf{Q}}(r) = \frac{\langle \mathbf{Q}(\mathbf{x}) : \mathbf{Q}(\mathbf{x}') \rangle}{\langle \mathbf{Q}(\mathbf{x}) : \mathbf{Q}(\mathbf{x}) \rangle}, \tag{6}$$

where $r = |\mathbf{x} - \mathbf{x}'|$, and $\mathbf{x}$ and $\mathbf{x}'$ represent two lattice sites at distance r. To make sure that no holes are formed, we decrease the size of the simulation box to 120×120.

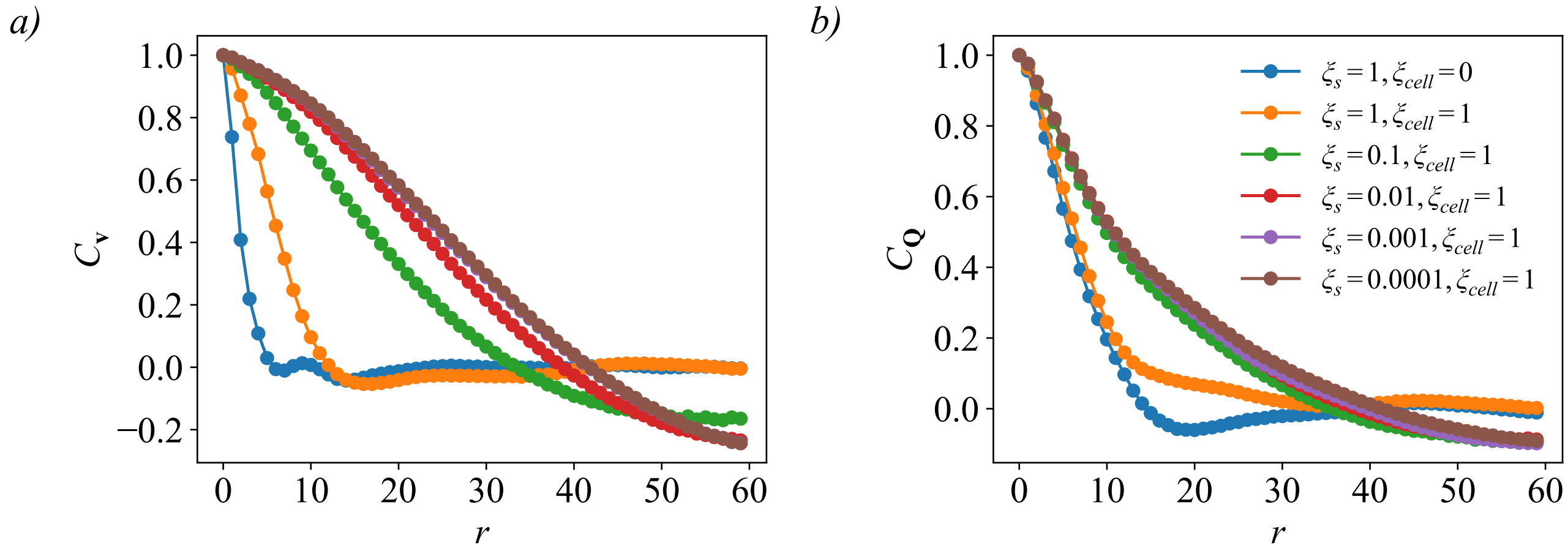

Supplementary Figure 1. a) Velocity and b) nematic director correlations as defined by Eqs. (5) and (6) for different values of substrate and cell-cell friction. The results for a) and b) were averaged over 3 samples with a simulation box of size 120×120.

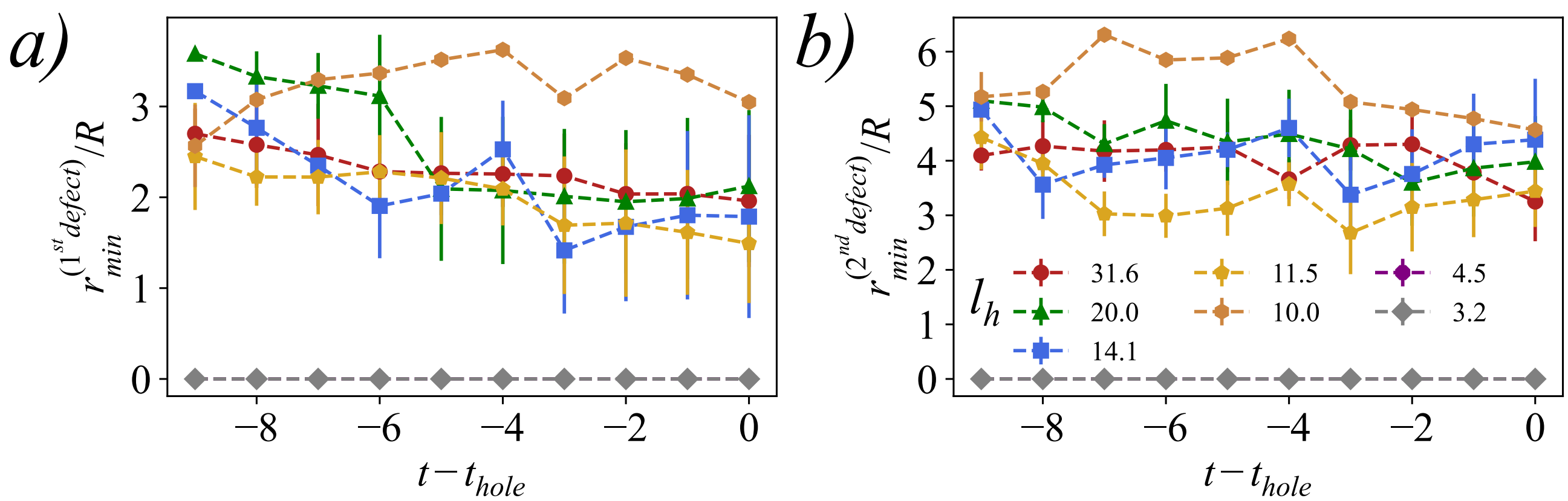

Supplementary Figure 2. a) and b) represent the distance from the hole's centroid to the closest and second closest $+1/2$ topological defects respectively, normalised by the cell radius R, as a function of the number of time-frames before hole nucleation, where 0 corresponds to a hole nucleation event. The results for a) and b) were averaged over 10 simulations with 100 time-frames each. The points at zero distance represent simulations which do not nucleate holes ($l_h = [3.2, 4.5]$).

C. Distance from hole nucleation to nematic topological defects

As discussed in the main text, hole formation arises from the collective flow of cells near spiral cores. In particular, spiral patterns are formed when two $+1/2$ topological defects in the nematic field move in the vicinity of each other. To investigate this, Fig. 2 shows the minimum distance between the hole nucleation centroid and the two nearest $+1/2$ defects during the time leading up to nucleation. While the hole does not always nucleate precisely between the defects, it remains consistently closer to two defects, with both defects typically located within approximately two cell diameters. These results support the role of $+1/2$ topological defect pairs in driving hole formation.

D. Surface tension decreases hole nucleation probability

In this section, we highlight that hole formation can be viewed as an activity-driven nucleation process, i.e. the active forces of the cells need to overcome the energy barrier imposed by the elasticity of the monolayer. To support this statement, we increase the elasticity of the monolayer through the surface tension of the cells (γ in the Cahn-Hilliard free energy term in Eq. (2) in the main text). This parameter decreases the deformability of the cells, making the elastic response stronger. Figure 3 shows that as γ increases, both the average number and the average maximum area of holes decrease.

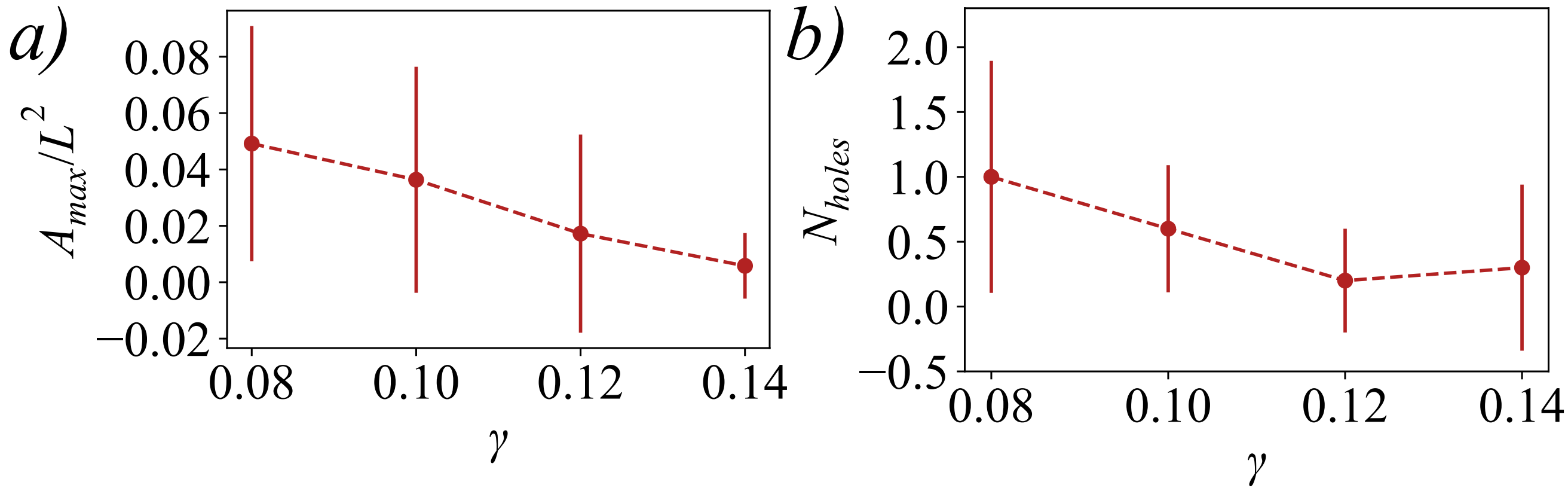

Supplementary Figure 3. a) Normalised maximum hole area and b) total number of holes integrated over the whole simulation as a function of the surface tension of the cells, for $l_h = 31.6$ and $\zeta = 0.3$. The results for a) and b) were averaged over 10 simulations with 100 time-frames each.

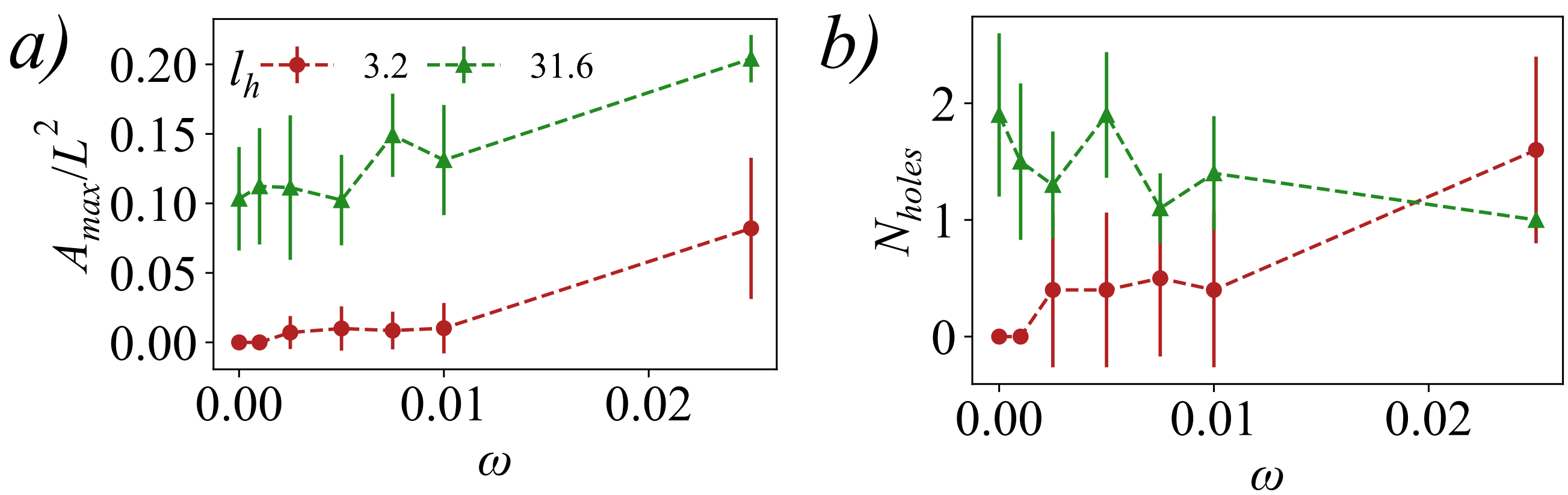

Supplementary Figure 4. Maximum area and total number of holes (a and b respectively), as a function of adhesion, for $l_h = [3.2, 31.6]$ and $\zeta = 0.3$. The results for a) and b) were averaged over 10 trajectories with 100 time-frames each.

E. Effects of an adhesion term in the free energy.

As in previous work [2], we introduce a free-energy term that mimics cell–cell adhesion:

$$\mathscr{F}_{adh} = \sum_i \sum_{j \neq i} \omega \lambda \int d\mathbf{x} \nabla \phi_i^2(\mathbf{x}) \nabla \phi_j^2(\mathbf{x}). \tag{7}$$

In Fig. 4, we show the average maximum area and average number of holes as a function of cell–cell adhesion. We observe that the results mirror the ones shown in Fig. 3b and c of the main text. This occurs because cells adhere more strongly with increasing ω, thereby increasing correlations [6]. In this sense, adhesion has an effect similar to reducing substrate friction (from the perspective of correlation length), which enhances the likelihood of hole formation. If adhesion becomes sufficiently large, the monolayer enters a jammed state. In this regime, small gaps may appear, as reported in previous work [11], but these arise from packing constraints rather than from cell dynamics. Moreover, the typical size of these gaps is on the order of, or smaller than, the cell size, suggesting that no nucleation process underlies these events.

F. Finite-size effects

To verify that boundary effects are negligible, we simulate larger systems with $N = 225$ and $N = 400$. Figure S5 shows the characteristic isotropic strain rate for hole nucleation for all three sizes on a log–linear plot, demonstrat-

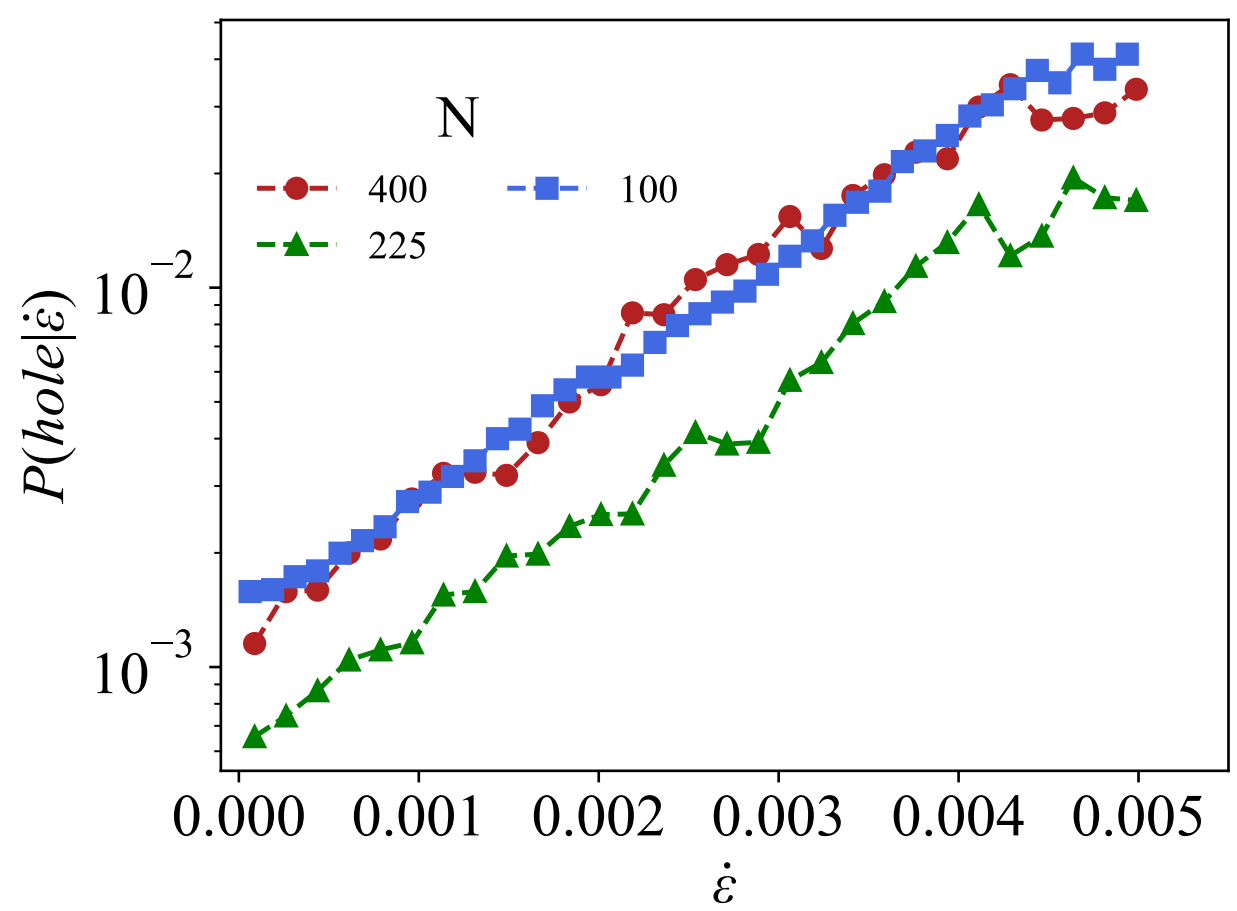

Supplementary Figure 5. **Characteristic isotropic strain rate for hole nucleation for different system sizes**. In the plot are shown curves for different system sizes, $N = (100, 225, 400)$, for the same set of parameters, $\zeta = 0.5$ and $l_h = 31.6$. The results for $N = 100$ are the same as the ones shown in Fig. 2. For $N = 225$ and $N = 400$, the distributions are averaged over 10 different simulations with 100 different time-frames.

ing that the exponential dependence is preserved for the larger system sizes. Moreover, the slopes of the curves, corresponding to the characteristic isotropic strain rate, are nearly identical, indicating that the boundary does not produce any noticeable effect.

A small vertical offset is present between the different system sizes, which we attribute to differences in the solver used for the matrix inversion required to evaluate Eq. (5). For the large matrices that must be inverted, we employ approximate conjugate-gradient methods to obtain solutions within reasonable time, as exact methods become prohibitively slow at low substrate friction values. This slowdown arises because the diagonal entries of the matrix become small compared to the off-diagonal terms. To compensate, we use a lower precision for the $N = 400$ case in order to simulate the same total number of time-steps in feasible computational time. For $N = (100, 225)$ the precision used is 0.00001, whereas for $N = 400$ it is 0.0001. We consider that the observed discrepancy in the figure is due to this difference in numerical precision.

G. Shear strain rate

In the main text, we focus on the role of the isotropic strain rate in hole formation. Here, we perform an analogous analysis for the shear strain rate, defined as

$$\dot{\varepsilon}_s = \sqrt{(\partial_x v_x - \partial_y v_y)^2 + (\partial_x v_y + \partial_y v_x)^2}. \tag{8}$$

Figure 6a shows that the maximum shear strain rate also peaks near the location and time of hole nucleation (see Supplementary Video S5), indicating that this event coincides with a localization of the total deformation within the tissue. Thus, as expected, the nucleation site corresponds to the region where the tissue undergoes the strongest overall distortion. In Fig. 6b we present the statistics of the shear strain rate at the onset of hole formation. In contrast to the isotropic component, which follows an exponential distribution, the shear strain rate exhibits a more complex, possibly bimodal, distribution. This behaviour likely reflects the heterogeneous nature of cell deformations in the vicinity of the nucleation site, where regions of strong distortion coexist with less deformed areas.

Taken together, these observations suggest that the isotropic and shear strain rates play distinct but complementary roles in hole creation. The isotropic strain rate captures the local dilation required to overcome the forces maintaining confluency and is therefore directly linked to hole nucleation. In contrast, the shear strain rate quantifies local distortions of the tissue, which contribute to the formation of the spiral flow patterns observed in the velocity field and play an important role in the subsequent growth of the hole, where significant deformation of cells along the interface is required.

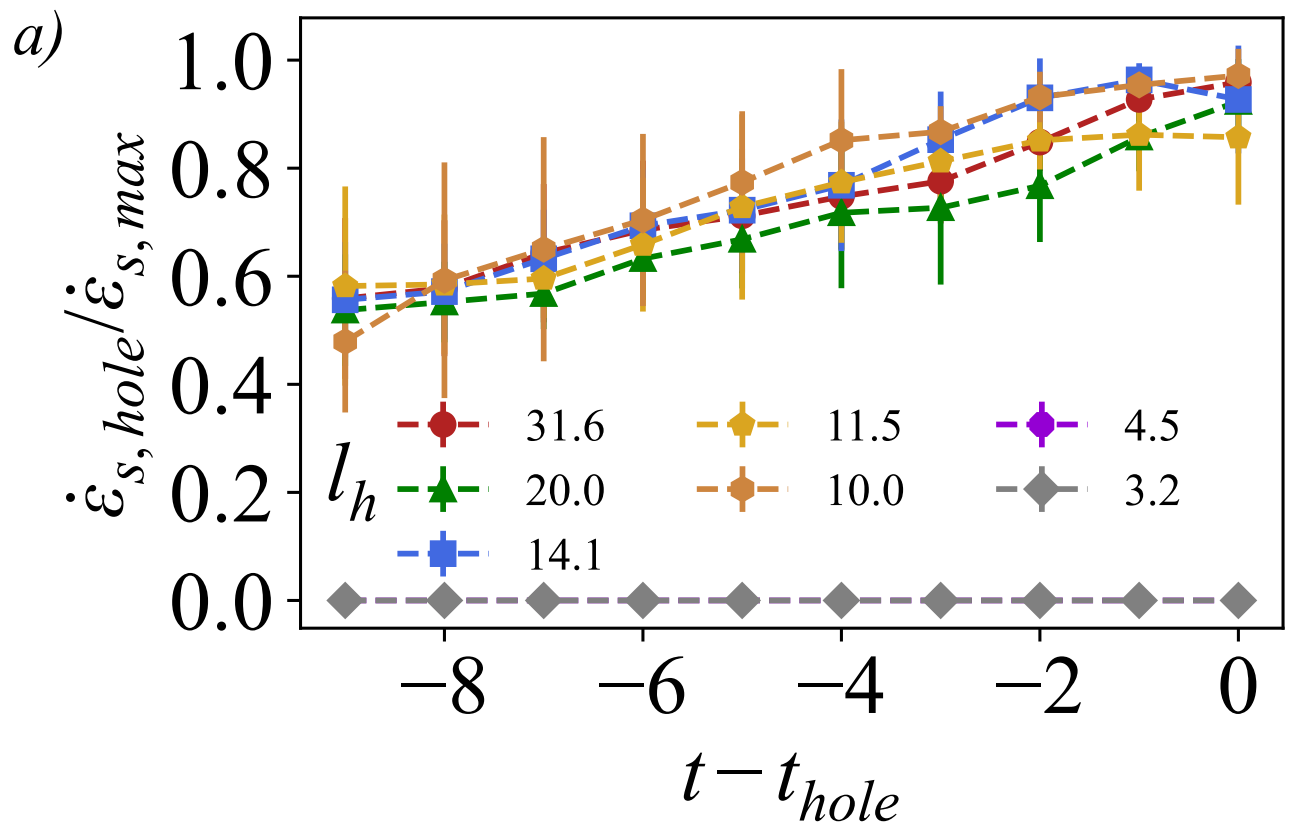

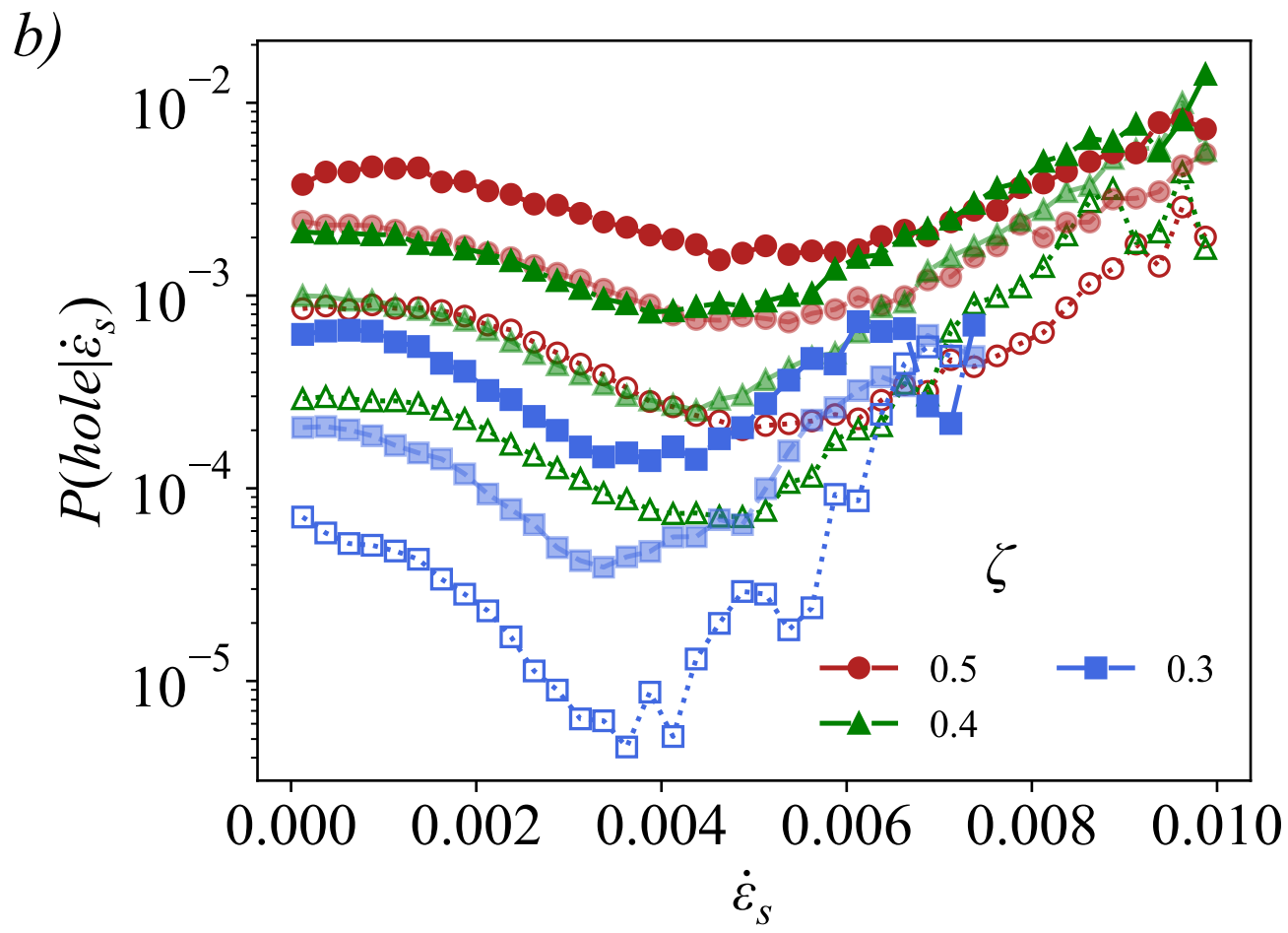

Supplementary Figure 6. **Statistics of the shear strain rate during hole formation.** a) Normalised maximum shear strain rate in an area of radius $R/2$ around the location of hole nucleation as a function of the number of time-frames before nucleation. The normalization is per time-frame, where each time-frame spans 1000 time-steps. These results were averaged over 10 simulations with 100 time-frames, and with constant $\xi_{cell} = 1$. b) Conditional probability of forming a hole for a given shear strain rate as a function of local shear strain rate and activity (empty symbols correspond to $\xi_s = 0.01$, partially transparent symbols to $\xi_s = 0.05$, and full symbols to $\xi_s = 0.001$). Each shear strain rate point corresponds to $\dot{\varepsilon}_s + \Delta\dot{\varepsilon}_s$, where $\Delta\dot{\varepsilon}_s = 0.000125$. Each distributions is averaged over 100 different simulations with 100 different time-frames.

[1] R. Mueller, J. M. Yeomans, and A. Doostmohammadi, Emergence of active nematic behavior in monolayers of isotropic cells, Physical Review Letters **122**, 048004 (2019).

[2] G. Zhang and J. M. Yeomans, Active forces in confluent cell monolayers, Physical Review Letters **130**, 038202 (2023).

[3] T. B. Saw, A. Doostmohammadi, V. Nier, L. Kocgozlu, S. Thampi, Y. Toyama, P. Marcq, C. T. Lim, J. M. Yeomans, and B. Ladoux, Topological defects in epithelia govern cell death and extrusion, Nature **544**, 212 (2017).

[4] A. J. Vromans and L. Giomi, Orientational properties of nematic disclinations, Soft Matter **12**, 6490 (2016).

[5] G. Zhang, R. Mueller, A. Doostmohammadi, and J. M. Yeomans, Active inter-cellular forces in collective cell motility, Journal of the Royal Society Interface **17**, 20200312 (2020).

[6] M. Chiang, A. Hopkins, B. Loewe, D. Marenduzzo, and M. C. Marchetti, Multiphase field model of cells on a substrate: From three dimensional to two dimensional, Physical Review E **110**, 044403 (2024).

[7] M. Chiang, A. Hopkins, B. Loewe, M. C. Marchetti, and D. Marenduzzo, Intercellular friction and motility drive orientational order in cell monolayers, Proceedings of the National Academy of Sciences **121**, e2319310121 (2024).

[8] A. Doostmohammadi, J. Ignés-Mullol, J. M. Yeomans, and F. Sagués, Active nematics, Nature Communications **9**, 3246 (2018).

[9] M. C. Marchetti, J. F. Joanny, S. Ramaswamy, T. B. Liverpool, J. Prost, M. Rao, and R. A. Simha, Hydrodynamics of soft active matter, Reviews of Modern Physics **85**, 1143 (2013).

[10] K. Thijssen, M. R. Nejad, and J. M. Yeomans, Role of friction in multidefect ordering, Physical Review Letters **125**, 218004 (2020).

[11] A. Ardaševa, R. Mueller, and A. Doostmohammadi, Bridging microscopic cell dynamics to nematohydrodynamics of cell monolayers, Soft Matter **18**, 4737 (2022).
