# Supplementary material for: Multi-phase field model reveals internal dissipation is crucial for spontaneous hole formation in cell monolayers
Source: Nat Commun. 2026 Jul 3;17:8288. doi: 10.1038/s41467-026-74977-y (PMC13469578; doi:10.1038/s41467-026-74977-y)
Supplement: Supplementary file 4 [file 41467_2026_74977_MOESM9_ESM.pdf]

Title: Supplementary Movie 1

Description: Animation of a simulation where multiple holes are able to nucleate. The line over each cell represents the (normalized) nematic director. These simulations are for $l_h=31.6$ and $\zeta=0.3$.

Title: Supplementary Movie 2

Description: Velocity field for the same simulation shown in Supplementary Video S1. The color scheme highlights the isotropic strain rate as in Fig.~1b of the main text. The spiral cores are shown in cyan. These simulations are for $l_h=31.6$ and $\zeta=0.3$.

Title: Supplementary Movie 3

Description: Active force field for the same simulation shown in Supplementary Video S1. The color scheme highlights the divergence of the local force field as in Fig.~1b of the main text. These simulations are for $l_h=31.6$ and $\zeta=0.3$.

Title: Supplementary Movie 4

Description: Nematic field for the same simulation shown in Supplementary Video S1. The color scheme highlights the isotropic strain rate as in Fig.~1b of the main text. The $+1/2$ defects are shown in green, and the $-1/2$ defects in blue. These simulations are for $l_h=31.6$ and $\zeta=0.3$.

Title: Supplementary Movie 5

Description: Velocity field for the same simulation shown in Supplementary Video S1. The color scheme highlights the shear strain rate. The spiral cores are shown in cyan. These simulations are for $l_h=31.6$ and $\zeta=0.3$.

Title: Supplementary Movie 6

Description: Active force field for the same simulation shown in Supplementary Video S1 without performing the smoothing window averaging. The color scheme highlights the divergence of the local force field as in Fig.~1b of the main text. These simulations are for $l_h=31.6$ and $\zeta=0.3$.

Title: Supplementary Movie 7

Description: Animation of a simulation where a long-lived hole nucleates. The line over each cell represents the (normalized) nematic director. These simulations are for $l_h=31.6$ and $\zeta=0.5$.
